# Synthesis of Heterometallic Zirconium Alkoxide Single-Source Precursors for Bimetallic Oxide Deposition

**DOI:** 10.1021/acs.inorgchem.2c02852

**Published:** 2022-11-16

**Authors:** Jonathan Slaughter, Chloe Coates, George Phillips, Dipanjana Choudhury, Andrew D. Bond, Clare P. Grey, Dominic S. Wright

**Affiliations:** † Yusuf Hamied Department of Chemistry, University of Cambridge, Lensfield Road, Cambridge CB2 1EW, United Kingdom; ‡ The Faraday Institution, Quad One, Harwell Science and Innovation Campus, Didcot OX11 0RA, United Kingdom

## Abstract

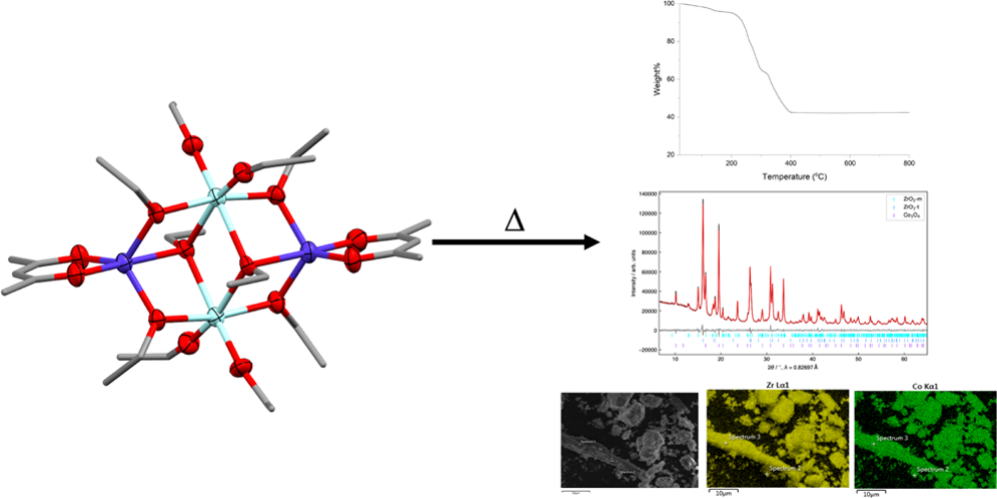

Single-source precursors are ubiquitous in a number of areas of chemistry and material science due to their ease of use and wide range of potential applications. The development of new single-source precursors is essential in providing entries to new areas of chemistry. In this work, we synthesize nine new structurally related bimetallic metal-zirconium alkoxides, which can be used as single-source precursors to zirconia-based materials. Detailed analysis of the structures of these complexes provides important insights into the main factors influencing their aggregation. Investigation of the thermal decomposition of these species by TGA, PXRD, SEM, and EDS reveals that they can be used to produce bimetal oxides, such as Li_2_ZrO_3_, or a mixture of metal oxides, such as CuO and ZrO_2_. Significantly, these studies show that thermodynamically unstable forms of zirconia, such as the tetragonal phase, can be stabilized by metal doping, providing the promise for targeted deposition of zirconia materials for specific applications.

## Abstract 2

The synthesis and structural characterization of nine new metal-zirconium alkoxides have been investigated with a range of s-, d-, and p-block metals. The thermal decomposition of these species was studied and revealed a range of products, including different polymorphs of zirconia, which are influenced by the accompanying metal through doping.

## Introduction

Metal oxides are used extensively in a very broad range of applications in the modern world, such as in batteries,^1^ solar cells,^2^ catalysts,^3^ and sensors.^4^ The varied properties of metal oxides stem from complex factors associated with the bonding and electronic properties of the metals involved. For example, zirconium oxide is a hard ceramic used as an enamel,^5^ whereas zinc oxide can act as a wide-band-gap semiconductor, which can be used in solar cells.^6^ The combination of two different metals within the oxide to produce bimetallic oxides can lead to a large number of possible materials, which can combine the features of both metal components. Through the choice of specific metals, even greater specialization of applications can be achieved. This has recently been investigated in the use of protective coatings for battery electrodes, such as the use of LiAlO_2_ coatings on cathodes. This bimetallic oxide coating can prevent degradation of the electrode due to its inert nature, similar to Al_2_O_3_, and also allows lithium diffusion through the coating, resulting in enhanced electrochemical performance compared to uncoated and Al_2_O_3_-coated cathodes.^7^ Bimetallic oxides are also of interest in water splitting. For example, the bimetallic oxide Fe_1.89_Mo_4.11_O_7_ was shown to be a highly efficient electrocatalyst for the hydrogen evolution reaction.^8^ The other component of water splitting, the oxygen evolution reaction, has been shown to be catalyzed by MFe_2_O_4_ (M = Co, Ni, Cu, Mn), with all of these bimetallic oxides showing better catalytic performance than Fe_2_O_3_.^9^ Even small amounts of another metal can strongly affect a monometallic oxide. For example, the doping of TiO_2_ with transition metals has been widely investigated due to the reduction in the band gap upon doping, allowing the band gap to fall within the visible region and producing a high-performance photocatalyst.^10,11^

The area of single-source precursors (SSPs) is a well-established field in chemistry, with the ability to produce complex materials by thermal or chemically activated decomposition of a single-molecular species. Not surprisingly, this strategy has been used extensively for the deposition of functional films of metal oxides and mixed-metal oxides for a range of applications. Advantages of using SSPs in this setting include the possibility for lower temperature oxide deposition, greater atomic, compositional and morphological control of the deposited material, and the possibility of using high-throughput techniques (such as spray coating) for large-scale and large-area device manufacture.^12,13^ Conventional routes to ceramic materials often require the use of oxides, nitrates, or carbonates, which require very high temperatures, especially if a product needs to be highly crystalline.^14^ Developing areas of research are the use of SSPs in the fabrication of multijunction water-splitting cells and in the synthesis of battery cathodes. A recent example of the use of an SSP is the production of nanostructured BiVO_4_ photoanode films for water oxidation from the low-temperature decomposition of the vanadate cage compound Bi_4_(DMSO)_12_V_13_O_40_H_3_. This method was shown to produce an even distribution of elements and, significantly, can be used to deposit large-area water-splitting devices.^15^ The decomposition of Ni_2_Ti_2_(OEt)_8_(acac)_4_ (acac = actetylacetonate) was also shown to produce NiTiO_3_, another valuable component for photooxidation of water.^16^ In the battery field, the thermal decomposition of LiCo(acac)_3_ was shown to produce crystalline LiCoO_2_, a common lithium-ion battery cathode.^17^

Pertinent to the current work, zirconium alkoxides have been used extensively as SSPs for the production of zirconia films by solution hydrolysis or thermal decomposition. A particular area of interest has been the coating of the cathode material NMC811 (LiNi_0.8_Mn_0.1_Co_0.1_O_2_) for the next generation of high-energy-density lithium-ion batteries. Zirconia-coated NMC811 has shown enhanced electrochemical behavior due to the chemical inertness of the zirconia, which slows the decomposition of the cathode.^18^ Additionally, zirconium alkoxides have been used to produce zirconia coatings on stainless steel sheets, improving their heat resistance against oxidation.^19^

The combination of zirconium alkoxides with other organometallic compounds has produced a number of heterometallic zirconium alkoxides, which can be used as SSPs for the production of metal-zirconium bimetallic oxides. For example, decomposition of the cobalt-zirconium species CoZr_2_(acac)_2_(O^i^Pr)_8_ under autogenic pressure resulted in the formation of spherical ZrO_2_ and Co particles, covered in carbon.^20^ The thermal decomposition of the zinc-zirconium alkoxide Zr_3_Zn_7_O(OH)_3_(OR)_15_Cl_6_ has also been shown to result in the formation of ZrO_2_ crystallites on the surface of ZnO nanowires.^21^ A further example is the SSP [Cu_4_Zr_2_O_2_(dmae)_4_(OAc)_8_]·2H_2_O (dmae = *N*,*N*-dimethylaminoethanolato), which produced a CuZrO_3_-CuO composite using aerosol-assisted chemical vapor deposition.^22^ There are potentially a number of advantages of including transition metals into the zirconia coatings for battery applications, including modifying the ionic conductor behavior of the film and increasing the stability of the protective layer, potentially via substitution of the transition metals into the surface and subsurface layers of the (active) electrode material. However, so far, this area has not been explored in the literature.

A prerequisite for the systematic study of the effects of inclusion of other metal dopants into zirconia for battery applications is an array of available SSPs, which include different metals. While a number of heterometallic zirconium alkoxides have been synthesized previously,^23−25^ in this work, we have prepared a wide range of new organically soluble heterometallic zirconium alkoxides, whose alkoxide peripheries should be readily hydrolyzed or thermolyzed to give metal-doped zirconia, bimetallic oxides, or a mixture of monometallic oxides. We explore their differing molecular structures systematically, which are highly dependent on the additional metal used, and investigate the decomposition of these materials into oxide phases. Overall, this study has generated a number of new zirconium-based SSPs which have the potential to be used in multiple areas of chemical deposition, and provides a first step to their applications in the battery area.

## Experimental Section

### General Procedures

All reactions were carried out under dry nitrogen, using a double-manifold vacuum line and a glovebox. Solvents were distilled over sodium (toluene) or sodium-potassium amalgam (THF and *n*-hexane) immediately before use. Anhydrous ethanol and *n*-propanol were purchased from Fisher Scientific and Alfa Aesar, respectively, and used as provided. Reagents were purchased from commercial suppliers (Sigma-Aldrich, Alfa Aesar, or Fisher Scientific) and used as provided. Reactions at −78 °C were achieved using a dry ice/acetone bath.

Solution NMR spectroscopic data were collected on either a Bruker Avance III HD 500 MHz Smart Probe NMR spectrometer or a Bruker Avance III HD 400 MHz NMR spectrometer. Spectra were obtained at 25 °C (unless otherwise stated) using deuterated solvents which were dried over molecular sieves (4 Å). For ^1^H and ^13^C NMR, chemical shifts are internally referenced to the deuterated solvent used and calculated relative to TMS.^26^ For ^7^Li and ^27^Al NMR, the chemical shifts are referenced to 9.7 M LiCl in D_2_O and 1.1 M Al(NO_3_)_3_ in D_2_O, respectively. Chemical shifts are expressed in δ ppm. The following abbreviations are used: br = broad, m = multiplet, q = quartet, s = singlet, sext = sextet, t = triplet.

^27^Al MAS NMR spectra were collected using a Hahn-echo pulse sequence (π–τ–2π–τ acquisition with a recycle delay of 0.73 μs). The sample was loaded into a 2.5 mm rotor and the experiments were conducted at a magic-angle spinning (MAS) frequency of 20 kHz using a 500 MHz (11.8 T) spectrometer (Bruker Avance III). The spectrum was externally referenced against AlF_3_ powder (−17 ppm) for ^27^Al.

Elemental analysis of carbon and hydrogen was performed using a PerkinElmer 240 Elemental Analyser or an Exeter Analytical CE-440 Elemental Analyser. Inductively coupled plasma optical emission spectrometry (ICP-OES) was run and analyzed on a Thermo Fisher Scientific iCAP7400 Duo ICP-OES spectrometer using Qtegra software. ICP standards were purchased from Sigma-Aldrich and nitric acid (trace-metal grade) from Fisher. Samples were dissolved in nitric acid (5 mL) at room temperature, then diluted with water (5 mL). A 0.5 mL aliquot was diluted to 10 mL with water and then analyzed.

X-ray crystallographic data were collected using either a Nonius KappaCCD (Mo Kα) or Bruker D8-QUEST diffractometer equipped with an Incoatec IμS microsource (Cu Kα). The temperature was held at 180(2) K using an Oxford Cryosystems N_2_ cryostat. Data integration and reduction were undertaken with HKL Denzo/Scalepack (Nonius) or with SAINT in the APEX3 software suite (Bruker). Multiscan corrections were applied using SORTAV (Nonius) or SADABS (Bruker). Structures were solved using SHELXT and refined using SHELXL.

Thermogravimetric analysis (TGA) of samples was performed on a Mettler Toledo TGA/DSC 2 STAR^e^ System. Samples of 10–20 mg were heated to 800 °C at a rate of 10 °C min^–1^. Measurements on samples were performed under a constant flow (80 mL min^–1^) of air (19–22% O_2_ in N_2_, <10 ppm H_2_O), provided by Air Liquide UK Limited.

Infrared spectroscopy was carried out on a PerkinElmer Spectrum One FTIR Spectrometer fitted with a PerkinElmer ATR sampling accessory.

The UV–visible absorbance data were acquired on a VARIAN Cary 50 Bio UV–visible Spectrophotometer, using 0.01 M solutions of the complexes in *n*-hexane.

Synchrotron powder X-ray diffraction (PXRD) patterns of the decomposition products were collected on the I11 beamline at Diamond Light Source using an energy of approximately 15 keV (0.826 Å). The data were collected over multiple beamtimes and each time the wavelengths and instrumental parameters were refined against a Si standard; the wavelength used is indicated for each refinement. The PXRD data for complex **9** which had been heated to 1000 °C were collected on a Malvern Panalytical Empyrean instrument, equipped with an X’celerator Scientific detector using non-monochromated Cu Kα radiation (1.5418 Å). Data were refined using Topas Academic v.6. For mixed-metal phases, the atomic displacement parameters were constrained to be the same. Since there is a strong correlation between the atomic displacement parameters and occupancies of different atoms, the degree of cation mixing in the cubic and tetragonal phases is considered not to be fully quantitative and is simply taken as an indication of doping stabilizing the higher-symmetry phases. For this reason, some of the elemental compositions do not follow the expected M/Zr 1:1 ratio (e.g., Fe, Co, Mg, and Ni), but the accuracy is sufficient to infer the phases present, so the refinements were not constrained further.

To investigate the size and structure of the powder particles, elemental distribution, scanning electron microscopy (SEM), and energy-dispersive X-ray spectroscopy (EDS) were used, employing a “TESCAN MIRA3” microscope. To fix the samples on the sample holders, a small amount of the powders were sprinkled onto graphite tapes, which were then sputtered with 10 nm thick chromium to improve conductivity and allow images to be taken. EDS analysis was performed with an X-MAX 150 mm^2^ detector (from Oxford Instruments) and processed with AZTEC software to produce maps of the elemental distributions. The accelerating voltage for these measurements was set to 20 kV.

### Synthesis of Li_2_Zr_2_(O^n^Pr)_10_(THF)_2_ (**1**)

Zr(O^n^Pr)_4_ (70 wt % in *n*-propanol, 0.90 mL, 2 mmol) and *n*-hexane (5 mL) were combined and cooled to −78 °C. *^n^*BuLi (1.6 M in *n*-hexanes, 1.25 mL, 2 mmol) was added dropwise, and the resulting solution was left to warm up to room temperature over the course of 2 h, producing a white suspension. The solvent was removed under vacuum, and the resulting white solid residue was dissolved in THF (0.5 mL). Storage of the solution at −20 °C for 16 h resulted in the formation of transparent crystals of **1** (290 mg, 31%). Elemental analysis calculated (%) for C_38_H_86_Li_2_O_12_Zr_2_: C 49.0, H 9.3. Found: C 46.2, H 9.1. ^1^H NMR spectroscopy (400 MHz, C_6_D_6_): δ 4.38–4.11 (20 H, m, OCH_2_), 3.69 (7.5 H, m, THF), 2.12–1.69 (20 H, m, CH_2_), 1.40 (7.5 H, m, THF), 1.22–0.98 (30 H, m, Me). ^13^C NMR spectroscopy (101 MHz, C_6_D_6_): δ 71.3, 70.0, 69.9 (OCH_2_), 68.3 (THF), 29.0, 28.8, 26.7 (CH_2_), 25.5 (THF), 11.2, 11.0, 10.4 (Me). ^7^Li NMR spectroscopy (155 MHz, C_6_D_6_): δ 0.6, 0.3.

### Synthesis of Mg_2_Zr_2_(O^n^Pr)_12_(^n^PrOH)_4_ (**2**)

Zr(O^n^Pr)_4_ (70 wt % in *n*-propanol, 3.6 mL, 8 mmol), *n*-propanol (2 mL), and *n*-hexane (20 mL) were combined and cooled to −78 °C. *^n^*Bu_2_Mg (1.0 M in *n*-heptane, 8 mL, 8 mmol) was added dropwise, and the resulting solution was left to warm up to room temperature overnight, producing a white suspension. The suspension was dissolved by gently heating the solvent. Storage of the solution at −20 °C for 16 h resulted in the formation of transparent crystals of **2** (3.30 g, 70%). Elemental analysis calculated (%) for C_48_H_116_Mg_2_O_16_Zr_2_: C 48.8, H 9.9. Found: C 49.4, H 10.1. ^1^H NMR spectroscopy (400 MHz, C_6_D_6_): δ 4.68 (4 H, s br, OH), 4.32–4.02 (24 H, m, OCH_2_), 3.53 (8 H, t, *J* = 7.0 Hz, OCH_2_), 1.97 (24 H, m, CH_2_), 1.55 (8 H, sext, *J* = 7.0 Hz, CH_2_), 1.10 (36 H, m, Me), 0.85 (12 H, t, *J* = 7.0 Hz, Me). ^13^C NMR spectroscopy (101 MHz, C_6_D_6_): δ 69.9, 64.9 (OCH_2_), 28.6, 26.8 (CH_2_), 11.0, 10.8, 10.5, 10.4, 10.3 (Me).

### Synthesis of Co_2_Zr_2_(OEt)_10_(acac)_2_ (**3**)

Zr(OEt)_4_ (1.09 g, 4 mmol), Co(acac)_2_ (514 mg, 2 mmol), and toluene (5 mL) were heated to reflux for 1 h, producing a purple solution. The toluene was removed under vacuum, and the resulting purple solid was redissolved in *n*-hexane (5 mL) with heating. Slow cooling of the solution to room temperature for 16 h resulted in the formation of purple crystals of **3** (540 mg, 57% wrt Co(acac)_2_). Elemental analysis calculated (%) for C_30_H_64_Co_2_O_14_Zr_2_: C 38.0, H 6.8. Found: C 37.6, H 6.8.

### Synthesis of Ni_2_Zr_2_(OEt)_8_(acac)_4_ (**4**)

Zr(OEt)_4_ (1.09 g, 4 mmol), Ni(acac)_2_ (514 mg, 2 mmol), and toluene (5 mL) were heated to reflux for 1 h, producing a green solution and a small amount of green solid. The solid was removed by filtration. The solution was concentrated to about 3 mL under vacuum. Storage of the material at −30 °C for 16 h resulted in the formation of green crystals of **4** (430 mg, 41% wrt Ni(acac)_2_). Elemental analysis calculated (%) for C_36_H_68_Ni_2_O_16_Zr_2_: C 40.9, H 6.5. Found: C 40.7, H 6.6.

### Synthesis of Fe_2_Zr_2_(OEt)_10_(acac)_2_ (**5**)

Zr(OEt)_4_ (1.09 g, 4 mmol), FeCl_2_ (507 mg, 4 mmol), KOEt (673 mg, 8 mmol), ethanol (2 mL), and toluene (10 mL) were heated to reflux for 2 h, producing a dark green solution and a white solid. Acetylacetone (0.41 mL, 4 mmol) was added, which turned the solution red, and the suspension was stirred for 1 h. The solvent was removed under vacuum and *n*-hexane (10 mL) was added. The white solid was removed by filtration, and the solution was concentrated to about 3 mL under vacuum. Storage of the solution at −20 °C for 16 h resulted in the formation of red crystals of **5** (365 mg, 19%). Elemental analysis calculated (%) for C_30_H_64_Fe_2_O_14_Zr_2_: C 38.2, H 6.8. Found: C 35.9, H 6.6.

### Synthesis of Cu_2_Zr_2_(OEt)_10_(acac)_2_ (**6**)

Zr(OEt)_4_ (1.09 g, 4 mmol), Cu(acac)_2_ (524 mg, 2 mmol), CuCl_2_ (269 mg, 2 mmol), KOEt (337 mg, 4 mmol), ethanol (2 mL), and toluene (10 mL) were heated to reflux for 2 h, producing a dark blue solution and a white solid. The white solid was removed by filtration, and the solvent was then removed under vacuum. The resulting blue residue was dissolved in *n*-hexane (2 mL). Storage of the solution at −30 °C for 16 h resulted in the formation of blue crystals of **6** (535 mg, 28%). Elemental analysis calculated (%) for C_30_H_64_Cu_2_O_14_Zr_2_: C 37.6, H 6.7. Found: C 37.6, H 6.7.

### Synthesis of Mn_1.67_Zr_2.33_(OEt)_10.66_(acac)_2_(EtOH)_1.34_ (**7**)

Zr(OEt)_4_ (1.09 g, 4 mmol), MnCl_2_ (503 mg, 4 mmol), KOEt (673 mg, 8 mmol), ethanol (2 mL), and *n*-hexane (10 mL) were heated to reflux for 2 h, producing an orange solution and a white solid. Acetylacetone (0.41 mL, 4 mmol) was added, and the suspension was stirred for 1 h. The white solid was removed by filtration and storage of the solution at −20 °C for 16 h, resulting in the formation of brown crystals of **7** (365 mg, 20% wrt Zr(OEt)_4_). Elemental analysis calculated (%) for C_34_H_75.34_Mn_1.67_O_16_Zr_2.33_: C 39.1, H 7.3, Mn 8.8, Zr 20.4. Found: C 39.0, H 7.6, Mn 10.4, Zr 20.0.

### Synthesis of Zn_2_Zr_2_(OEt)_10_(acac)_2_ (**8**)

Zr(OEt)_4_ (1.09 g, 4 mmol), ZnCl_2_ (545 mg, 4 mmol), KOEt (673 mg, 8 mmol), ethanol (2 mL), and toluene (10 mL) were heated to reflux for 2 h, producing a yellow solution and a white solid. Acetylacetone (0.41 mL, 4 mmol) was added, and the suspension was stirred for 1 h. The solvent was removed under vacuum, and *n*-hexane (10 mL) was added. The white solid was removed by filtration. Storage of the solution at −20 °C for 16 h resulted in the formation of colorless crystals of **8** (1.00 g, 52%). Elemental analysis calculated (%) for C_30_H_64_O_14_Zn_2_Zr_2_: C 37.4, H 6.7. Found: C 37.4, H 6.7. ^1^H NMR spectroscopy (400 MHz, C_6_D_6_): δ 5.19 (2 H, s, CH), 4.69 (4 H, q, *J* = 7.0 Hz, CH_2_), 4.33 (8 H, q, *J* = 6.9 Hz, CH_2_), 4.24 (8 H, q, *J* = 6.9 Hz, CH_2_), 1.82 (12 H, s, Me_acac_), 1.73 (6 H, t, *J* = 7.0 Hz, Me), 1.46 (12 H, t, *J* = 6.9 Hz, Me), 1.33 (3 H, t, *J* = 6.9 Hz, Me). ^13^C NMR spectroscopy (101 MHz, C_6_D_6_): δ 193.5 (C=O), 99.7 (CH), 65.5, 65.0, 64.1 (CH_2_), 28.1 (Me_acac_), 20.4, 19.4, 19.2 (Me).

### Synthesis of Al_2_Zr_2_(OEt)_10_(acac)_4_ (**9**)

Zr(OEt)_4_ (1.09 g, 4 mmol), Al(OEt)_3_ (649 mg, 4 mmol), acetylacetone (0.82 mL, 8 mmol), and toluene (5 mL) were heated to reflux for 30 min, producing a gray suspension. The suspension was filtered through Celite to give a yellow solution. The solvent was removed under vacuum, and the resulting yellow solid was redissolved in *n*-hexane (5 mL). Storage of the solution at −30 °C for 16 h resulted in the formation of colorless crystals of **9** (818 mg, 38%). Elemental analysis calculated (%) for C_40_H_78_Al_2_O_18_Zr_2_: C 44.3, H 7.2 Found: C 43.8, H 7.4. ^1^H NMR spectroscopy (400 MHz, C_6_D_6_): δ 5.44–5.15 (4 H, m, CH), 4.94–3.62 (20 H, m, CH_2_), 2.00–0.86 (54 H, m, Me). ^13^C NMR spectroscopy (101 MHz, C_6_D_6_): δ 191.9, 190.2, 189.8 (C=O), 100.5 (CH), 65.6, 61.5, 61.1, 60.3 (CH_2_), 32.0, 26.9, 26.4, 26.3, 26.2 (Me_acac_), 23.1, 21.1, 20.4, 18.9, 18.8, 14.3 (Me). ^27^Al NMR spectroscopy (104 MHz, C_6_D_6_): δ 34.3, 5.0.

## Results and Discussion

### Synthesis and Structural Analysis

**Figure 1:**
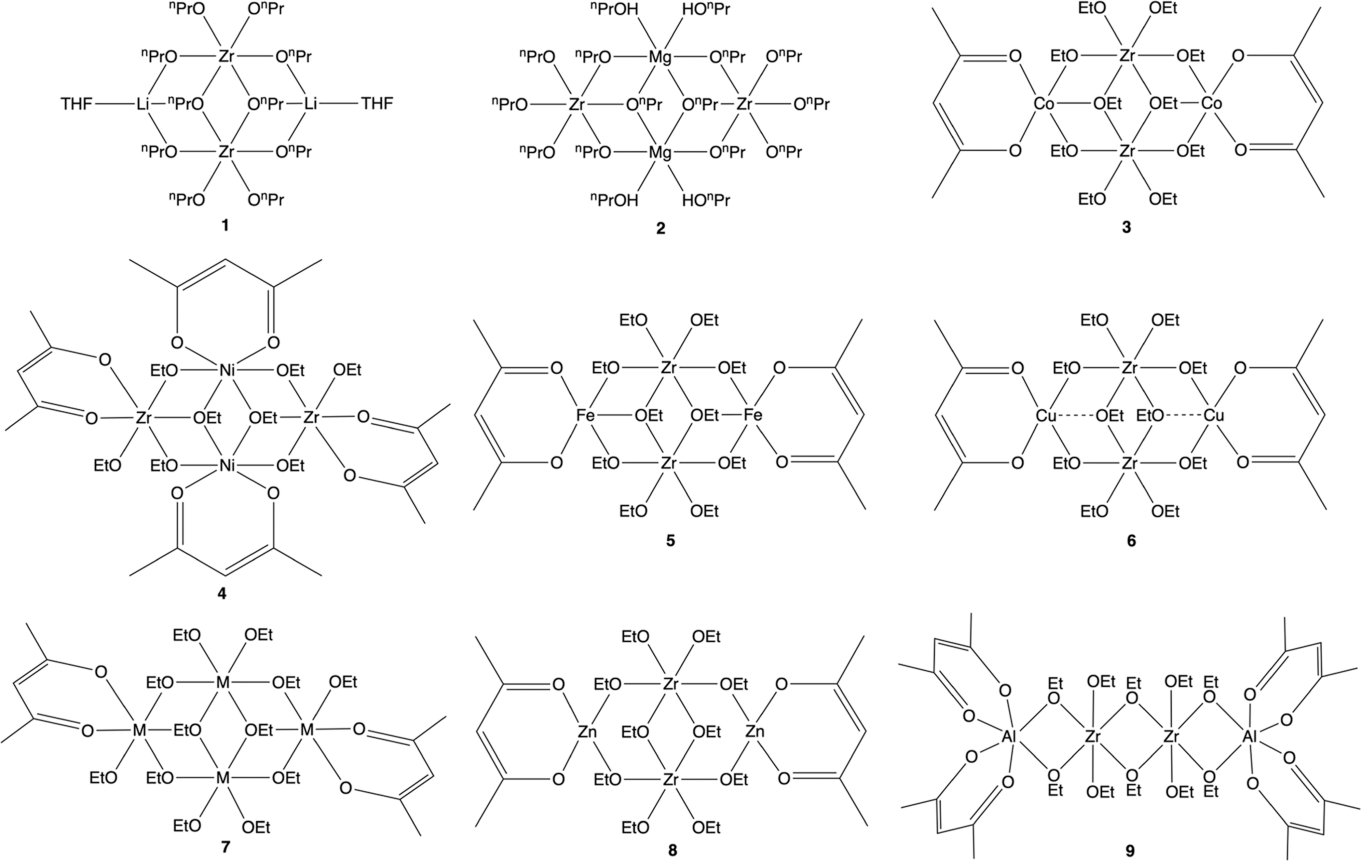
Structures of complexes **1**–**9**. Complex **7** has disorder of Zr and Mn at M sites.

A total of nine new heterometallic zirconium alkoxides were synthesized in the current work (Figure 1). A summary of the synthetic approaches involved is shown in the SI (Scheme S1).

**Figure 2:**
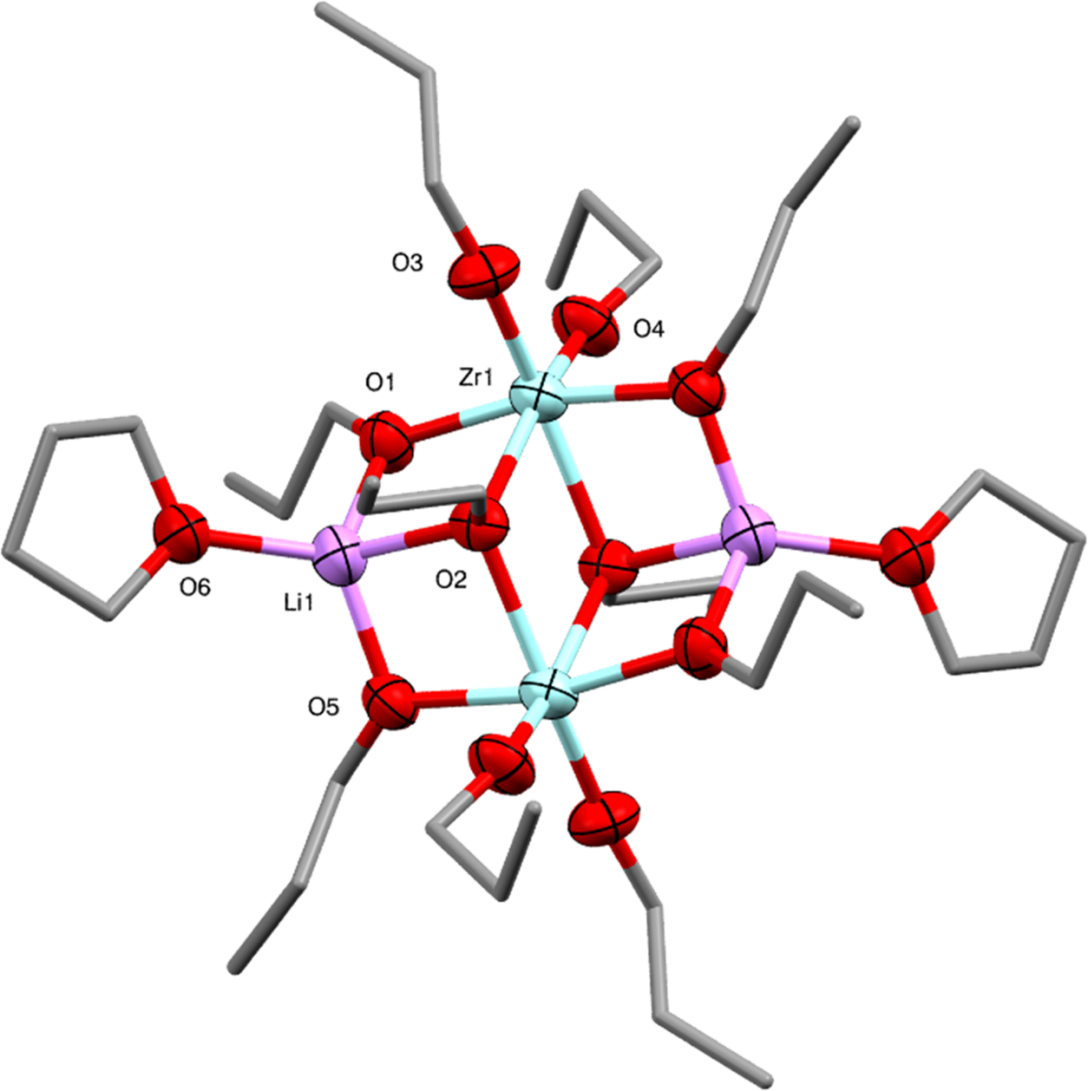
Molecular structure of Li_2_Zr_2_(O^n^Pr)_10_(THF)_2_ (**1**) (ellipsoids at 50% probability) with H atoms and minor disorder of some *n*-propoxide ligands omitted. Selected bond lengths (Å) and angles (°): Li1–O1 1.871(7), Li1–O2 2.087(8), Li1–O5 1.860(7), Li1–O6 1.950(7), Zr1–O1 2.070(3), Zr1–O2 2.247(2), Zr1–O2′ 2.249(2), Zr1–O3 1.940(3), Zr1–O4 1.951(3), Zr1–O5′ 2.084(3), O–Li1–O 88.3(3)-127.6(4), O–Zr1–O 70.37(9)-103.18(12).

Initial studies in this work focused on the synthesis of a lithium-zirconium alkoxide species which could act as a lithium-zirconium oxide SSP. The 1:1 reaction of *^n^*BuLi with Zr(O^n^Pr)_4_ in *n*-propanol and *n*-hexane resulted in the formation of a white solid which was insoluble in *n*-hexane and toluene but proved very soluble in THF. Storage of a THF solution at −20 °C resulted in the formation of the crystalline material Li_2_Zr_2_(O^n^Pr)_10_(THF)_2_ (**1**). The X-ray diffraction structure reveals a centrosymmetric molecule containing two lithium ions and two zirconium ions (Figure 2). From a chemical viewpoint, the structure can be seen as being composed of a [{(^n^PrO)_4_Zr(μ_2_-O^n^Pr)}_2_]^2–^ dianion which coordinates two THF-coordinated Li^+^ cations. This model and similar models discussed in this paper are based on the more covalent/directional nature of the Zr–O bonding, therefore making it the “dominant” fragment in these complexes in terms of its structural influence.^27^ Compound **1** is isostructural with the previously reported complex Li_2_Zr_2_(O^i^Pr)_8_(OMe)_2_(THF)_2_.^28^ The lithium ions have a distorted tetrahedral coordination geometry with one μ_3_-*n*-propoxide, two μ_2_-*n*-propoxides, and one THF. The zirconium ions have a distorted octahedral coordination geometry with two μ_3_-*n*-propoxides, two μ_2_-*n*-propoxides, and two terminal *n*-propoxides. Structurally, it is useful to take a polyhedral view of the coordination environments (see the SI). The central {Zr_2_O_10_} core of the complex comprises a pair of ZrO_6_ octahedra sharing one edge, with idealized point symmetry *D*_2h_. The Li^+^ cations define tetrahedra which attach to the {Zr_2_O_10_} core through one of their triangular faces (actually sharing one edge with each of the ZrO_6_ octahedra, see the SI) so that the point symmetry of the resulting {Zr_2_Li_2_O_12_} unit is reduced to *C*_2h_.

Both NMR spectroscopy and elemental analysis suggested fewer than two equivalents of THF present in the dried crystalline material (1.9 by ^1^H NMR spectroscopy and 1.0 by elemental analysis). It therefore appears that some of the coordinated THF is lost during drying of the crystalline material under vacuum prior to isolation and analysis. The ^1^H NMR spectrum of **1** (Figure S1) reveals a complex pattern of peaks, with ratios that do not correspond to the solid-state structure. Cooling the sample to −50 °C did not result in significant resolution of the ^1^H NMR spectrum (Figure S11), suggesting that in solution, **1** is in complex equilibrium with other species. This is also suggested by the ^7^Li NMR spectrum where two peaks are present (Figure S3).

**Figure 3:**
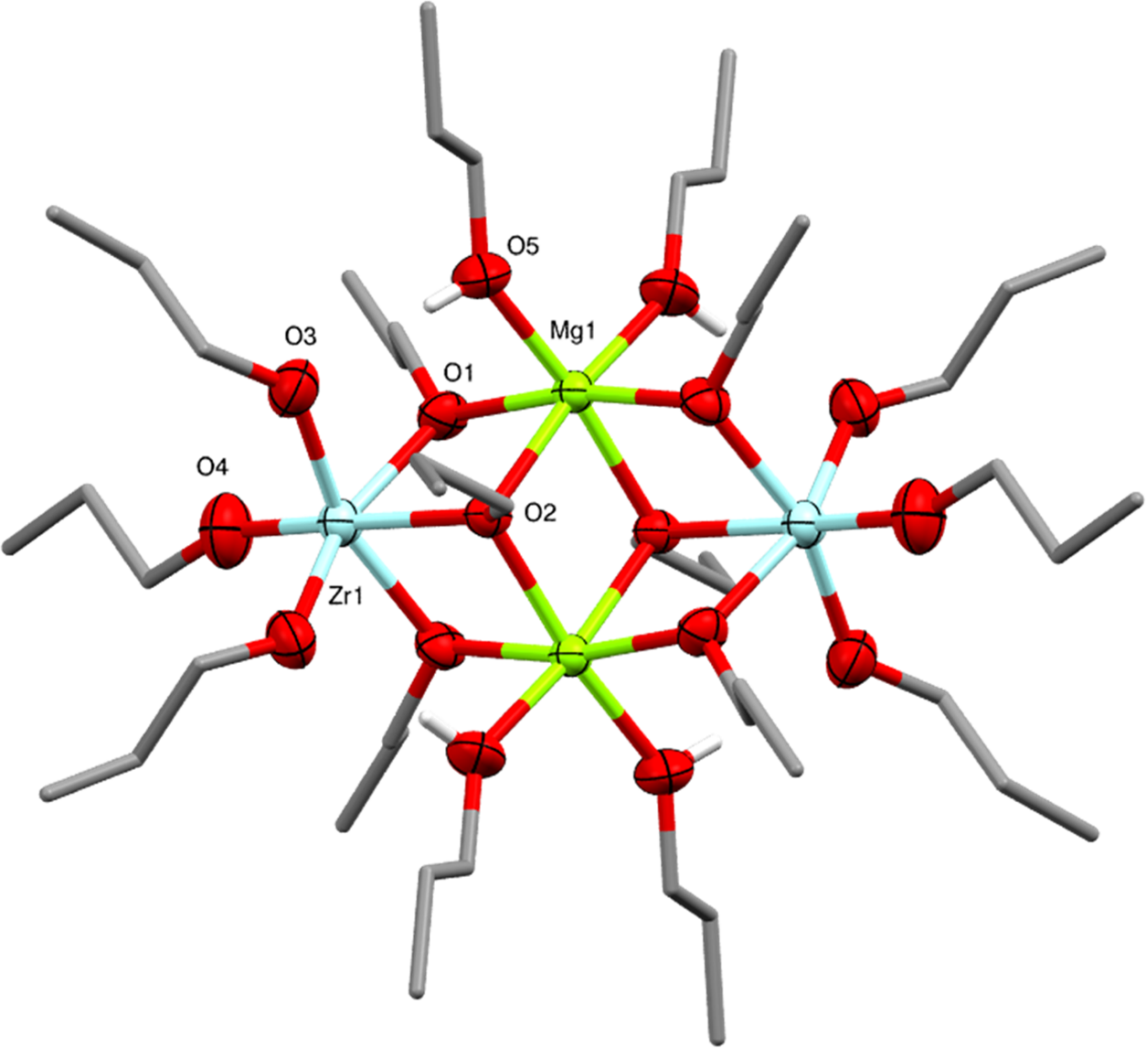
Molecular structure of Mg_2_Zr_2_(O^n^Pr)_12_(^n^PrOH)_4_ (**2**) (ellipsoids at 50% probability) with H atoms and minor disorder of some *n*-propoxide ligands omitted. The O–H hydrogens of coordinating alcohols are shown. Selected bond lengths (Å) and angles (°): Mg1–O1 2.030(5), Mg1–O2 2.183(6), Mg1–O5 2.061(6), Zr1–O1 2.129(6), Zr1–O2 2.201(7), Zr1–O3 2.043(7), Zr1–O4 1.930(9), O–Li1–O 88.3(3)-127.6(4), O–Zr1–O 76.14(19)–99.9(4).

**Figure 4:**
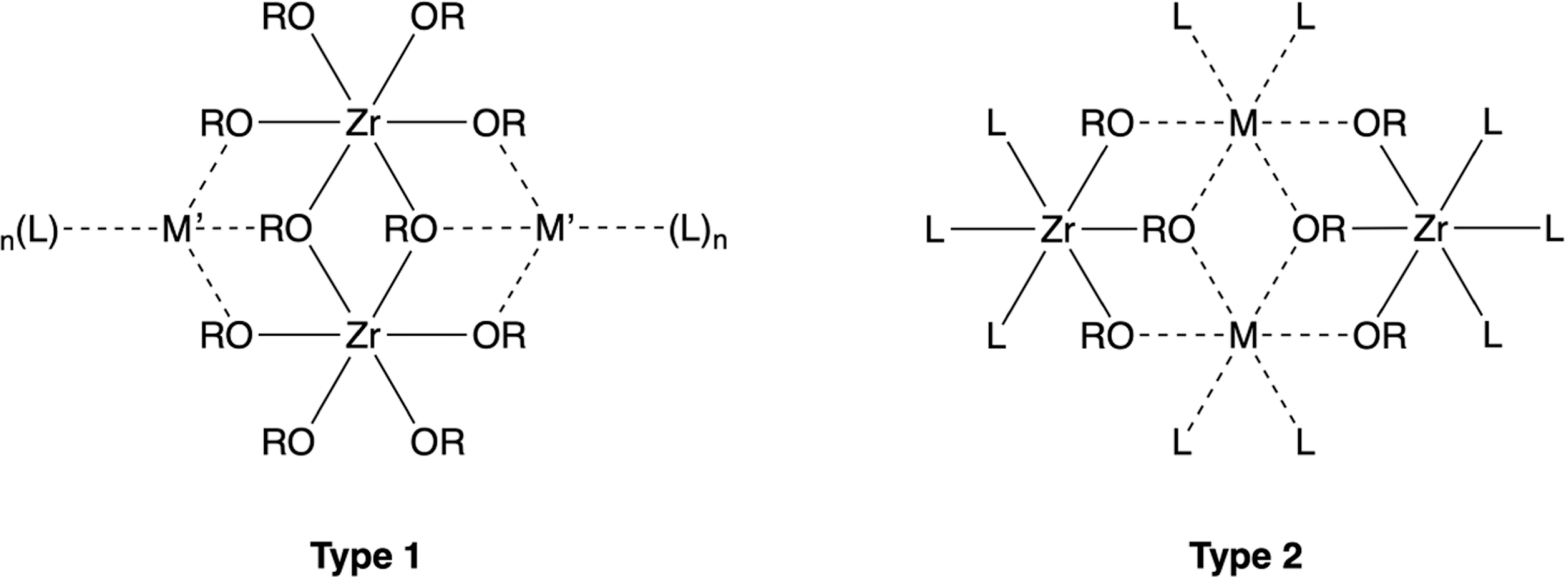
Two observed complex arrangements, type 1 containing a [{(RO)_4_Zr(μ_2_-OR)}_2_]^2–^ dianion and with the coordinated metal ions (M′) at the peripheral sites, and type 2 containing [Zr(OR)_6_]^2–^ or [Zr(OR)_4_(acac)]^−^ anions and with the coordinated metal ions (M) at the center of the complex.

The next target was the synthesis of a magnesium-zirconium alkoxide. This was achieved through the 1:1 reaction of *^n^*Bu_2_Mg with Zr(O^n^Pr)_4_ in *n*-propanol and *n*-hexane, with storage at −20 °C producing crystals of Mg_2_Zr_2_(O^n^Pr)_12_(^n^PrOH)_4_ (**2**). The solid-state structure of **2** (Figure 3) is isostructural with the previously reported ethoxide complex Mg_2_Zr_2_(OEt)_12_(EtOH)_4_^29^ and can be regarded as resulting from the coordination of two bis-^n^PrOH coordinated Mg^2+^ cations by two [Zr(O*^n^*Pr)_6_]^2–^ dianions. The presence of ^n^PrOH groups is confirmed in the IR spectrum of **2** (Figure S15), which shows a weak and broad O–H stretching band at ca. 3100 cm^–1^. Molecules of **2** and previously reported Mg_2_Zr_2_(OEt)_12_(EtOH)_4_ both have a M_4_O_16_ core which is prevalent in group IV alkoxides, such as titanium ethoxide.^30^ From a polyhedral viewpoint, the M_4_O_16_ structure comprises a pair of edge-sharing MgO_6_ octahedra, with two ZrO_6_ octahedra attached through one triangular face in the same manner as the LiO_4_ tetrahedra in **1**. The fact that Zr^4+^ occupies the central octahedral sites (denoted the M sites, see Figure 4) in **1**, but outer octahedral sites (denoted the M′ sites, see Figure 4) in **2** indicate that the coordination requirements and ionic radii of the metal cations (Li^+^ vs Mg^2+^) have a key influence. Quantitative measures of the coordination environments (see Table S2) indicate that the central ZrO_6_ octahedra in **1** are significantly more distorted from regular octahedral geometry compared to the central MgO_6_ octahedra in **2**. The possibility to form more regular octahedral geometry in the central M sites may be a driving force for Mg^2+^ to preferentially occupy these sites (see also the discussion for Ni^2+^ in **4**).

In contrast to the lability of the THF ligands in **1**, NMR spectroscopy and elemental analysis suggest the retention of the *n*-propanol ligands in **2**. However, similarly to **1** the ^1^H NMR spectrum of **2** (Figure S4) is more complex than the solid-state structure, suggesting multiple species or fluxionality in solution. Similar to **1**, cooling to −50 °C did not significantly resolve the ^1^H NMR spectrum (Figure S12).

**Figure 5:**
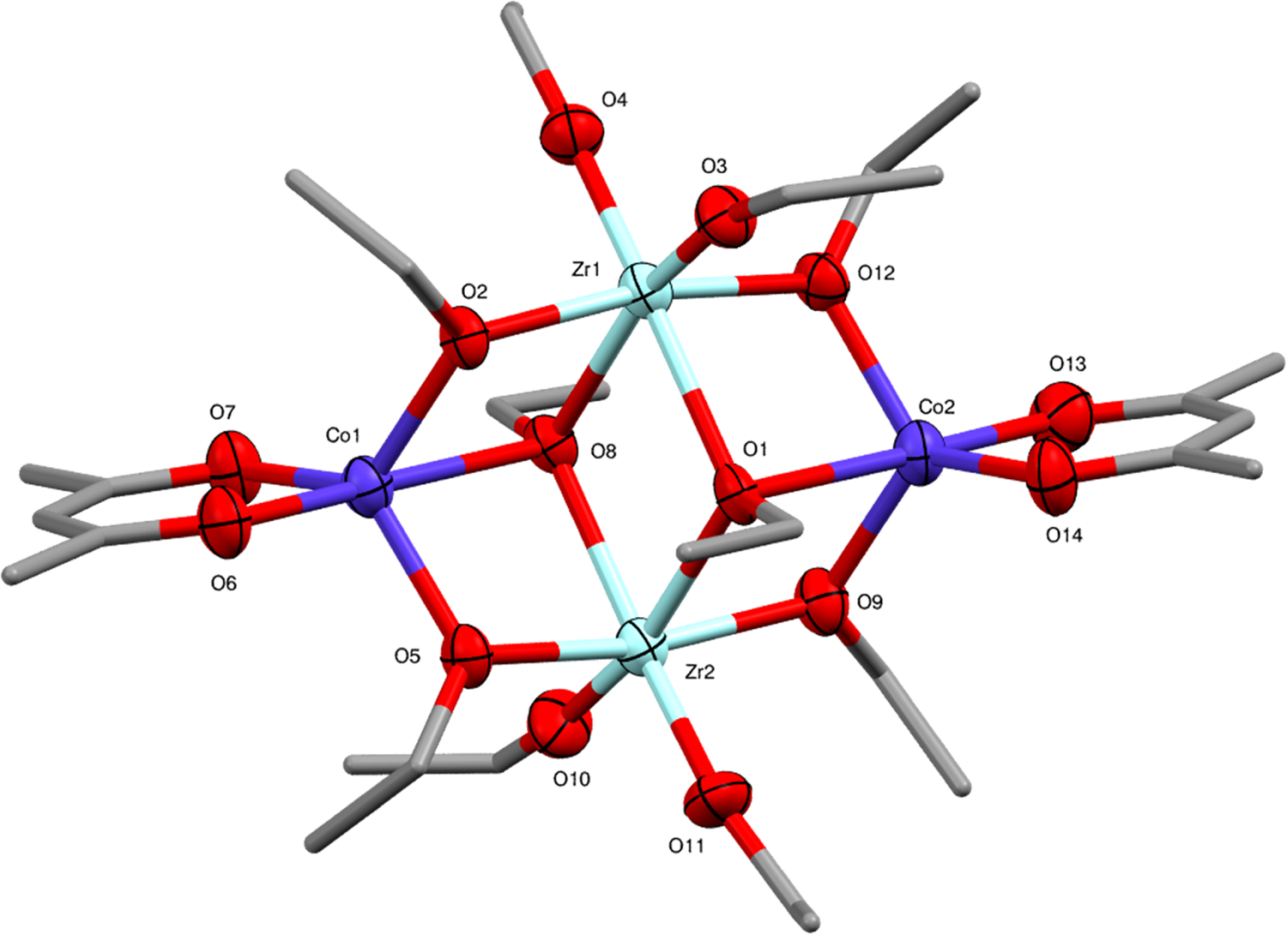
Molecular structure of Co_2_Zr_2_(OEt)_10_(acac)_2_ (**3**) (ellipsoids at 50% probability) with H atoms omitted. One representative molecule from the asymmetric unit is shown. The other molecule has essentially identical conformation. Selected bond lengths (Å) and angles (°): Co1–O2 1.957(6), Co1–O5 1.951(7), Co1–O6 2.015(8), Co1–O7 1.952(7), Co1–O8 2.404(6), Co2–O1 2.405(6), Co2–O9 1.953(7), Co2–O12 1.961(7), Co2–O13 2.001(8), Co2–O14 1.952(8), Zr1–O1 2.247(7), Zr1–O2 2.125(6), Zr1–O3 1.924(6), Zr1–O4 1.935(7), Zr1–O8 2.229(6), Zr1–O12 2.114(7), Zr2–O1 2.223(6), Zr2–O5 2.123(6), Zr2–O8 2.237(7), Zr2–O9 2.116(7), Zr2–O10 1.929(7), Zr2–O11 1.930(7), O–Co1–O 76.4(2)-125.2(3), O–Co2–O 76.5(3)-123.6(3), O–Zr1–O 69.7(2)-103.4(3), O–Zr2–O 69.9(2)–104.3(3).

Further studies using the same synthetic methodology involving reactions of Zr(O^n^Pr)_4_ with a range of transition-metal halides and potassium alkoxides proved unsuccessful, often producing intractable residues and no crystalline materials that could be characterized. Previous studies had, however, shown that the use of metal acetylacetonates as precursors in these reactions might be more successful.^23^ The addition of acetylacetonate ligands can lead to compounds with decreased solubility and greater stability to hydrolysis.^31^ Additionally, it was found that replacing Zr(O^n^Pr)_4_ with Zr(OEt)_4_ resulted in better crystallinity of the isolated compounds, making them amenable to X-ray analysis. The 2:1 stoichiometric reaction of Zr(OEt)_4_ and Co(acac)_2_ in toluene heated to reflux resulted in the formation of Co_2_Zr_2_(OEt)_10_(acac)_2_ (**3**) as a blue crystalline solid. Molecules of **3** are isostructural with the previously reported compounds Co_2_Zr_2_(O^n^Pr)_10_(acac)_2_^32^ and Co_2_Zr_2_(O^i^Pr)_6_(O^n^Pr)_4_(acac)_2_,^23^ which were synthesized in a similar manner to **3**. From the reaction stoichiometry, it is assumed that Zr(OEt)_3_(acac) is also formed as a side-product, which is more soluble in *n*-hexane than **3** and is therefore retained in solution during the crystallization step (Scheme S1). X-ray crystallography reveals two chemically similar, but crystallographically independent molecules in the lattice (one of which is shown in Figure 5). The overall structure of **3** is similar to that of **1**, being composed of a [{(EtO)_4_Zr(μ_2_-OEt)}_2_]^2–^ dianion that coordinates two [Co(acac)]^+^ fragments at the periphery of the Zr_2_Co_2_O_6_ core. The high-spin d^7^ Co^2+^ ions adopt a distorted trigonal-bipyramidal geometry, coordinated by one μ_3_-ethoxide, two μ_2_-ethoxides, and one acetylacetonate ligand. The average bond length to the μ_3_-ethoxide is significantly longer than the other Co–O bonds (2.405 Å, cf. 1.956 Å for the μ_2_-ethoxides). As in **1**, the Zr^4+^ ions are situated in the central M sites and have a distorted octahedral geometry, coordinated by two μ_3_-ethoxides, two μ_2_-ethoxides, and two terminal ethoxides. Quantitative measures (see Table S2) show that the distortion of the ZrO_6_ octahedra from regular geometry is the greatest of any of the compounds in this paper. From the polyhedral viewpoint, the CoO_5_ trigonal bipyramids are again attached to the central core through one of their triangular faces.

**Figure 6:**
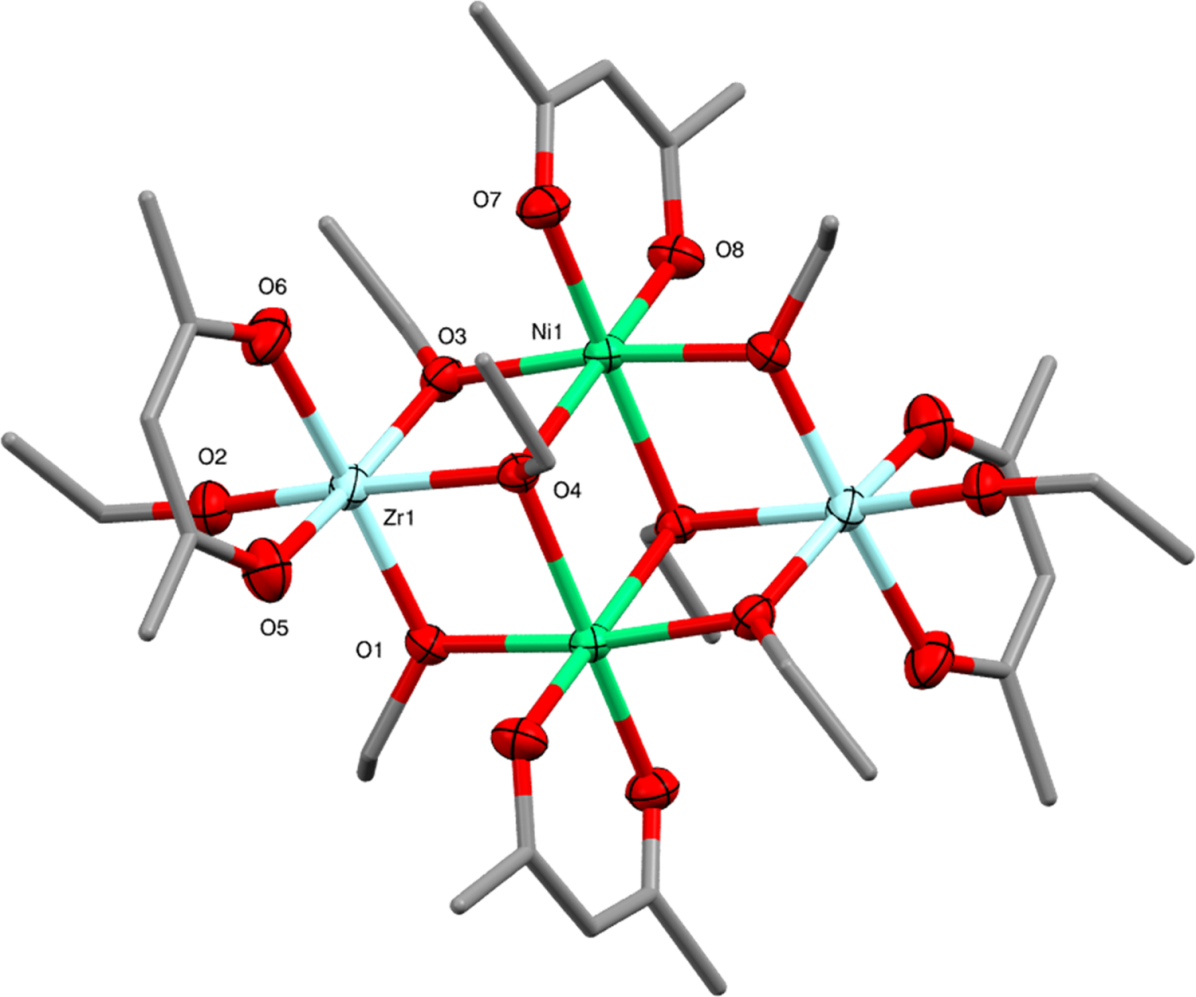
Molecular structure of Ni_2_Zr_2_(OEt)_8_(acac)_4_ (**4**) (ellipsoids at 50% probability) with H atoms omitted. Selected bond lengths (Å) and angles (°): Ni1–O1 2.0527(14), Ni1–O3 2.0698(14), Ni1–O4 2.1600(13), Ni1–O4′ 2.1892(13), Ni1–O7 1.9876(14), Ni1–O8 2.0016(14), Zr1–O1 2.0430(14), Zr1–O2 1.9270(15), Zr1–O3 2.0319(14), Zr1–O4 2.1866(13), Zr1–O5 2.1601(16), Zr1–O6 2.1676(15), O–Ni1–O 79.54(5)–96.12(5), O–Zr1–O 77.53(7)–97.10(6).

The same reaction using Ni(acac)_2_ instead of Co(acac)_2_ gave the complex Ni_2_Zr_2_(OEt)_8_(acac)_4_ (**4**). Interestingly, as shown by X-ray crystallography the centrosymmetric arrangement of this complex resembles that of **2**. Here, however, the [Zr(O^n^Pr)_6_]^2–^ dianions of **2** are replaced by [Zr(OEt)_4_(acac)]^−^ monoanions, in which two of the alkoxide ligands are substituted for a monoanionic acetylacetonate ligand. The [Zr(OEt)_4_(acac)]^−^ anions of **4** coordinate two [Ni(acac)]^+^ fragments at the center of the core (Figure 6). This arrangement gives the high-spin d^8^ Ni^2+^ ions a distorted octahedral geometry (being coordinated by two μ_3_-ethoxides, two μ_2_-ethoxides and chelated by an acetylacetonate ligand) and is isostructural with the previously reported nickel-titanium alkoxide complex Ni_2_Ti_2_(OEt)_8_(acac)_4_, which was obtained from the reaction of Ni(acac)_2_ with Ti(OEt)_4_.^33^ As seen for **2**, the Ni^2+^ ions in the central M sites show the most regular octahedral geometry of any complex in this paper (see Table S2), suggesting again that the occupation of the M vs the M′ sites may be influenced by the possibility for Ni^2+^ to form a more regular octahedral geometry in the M sites.

**Figure 7:**
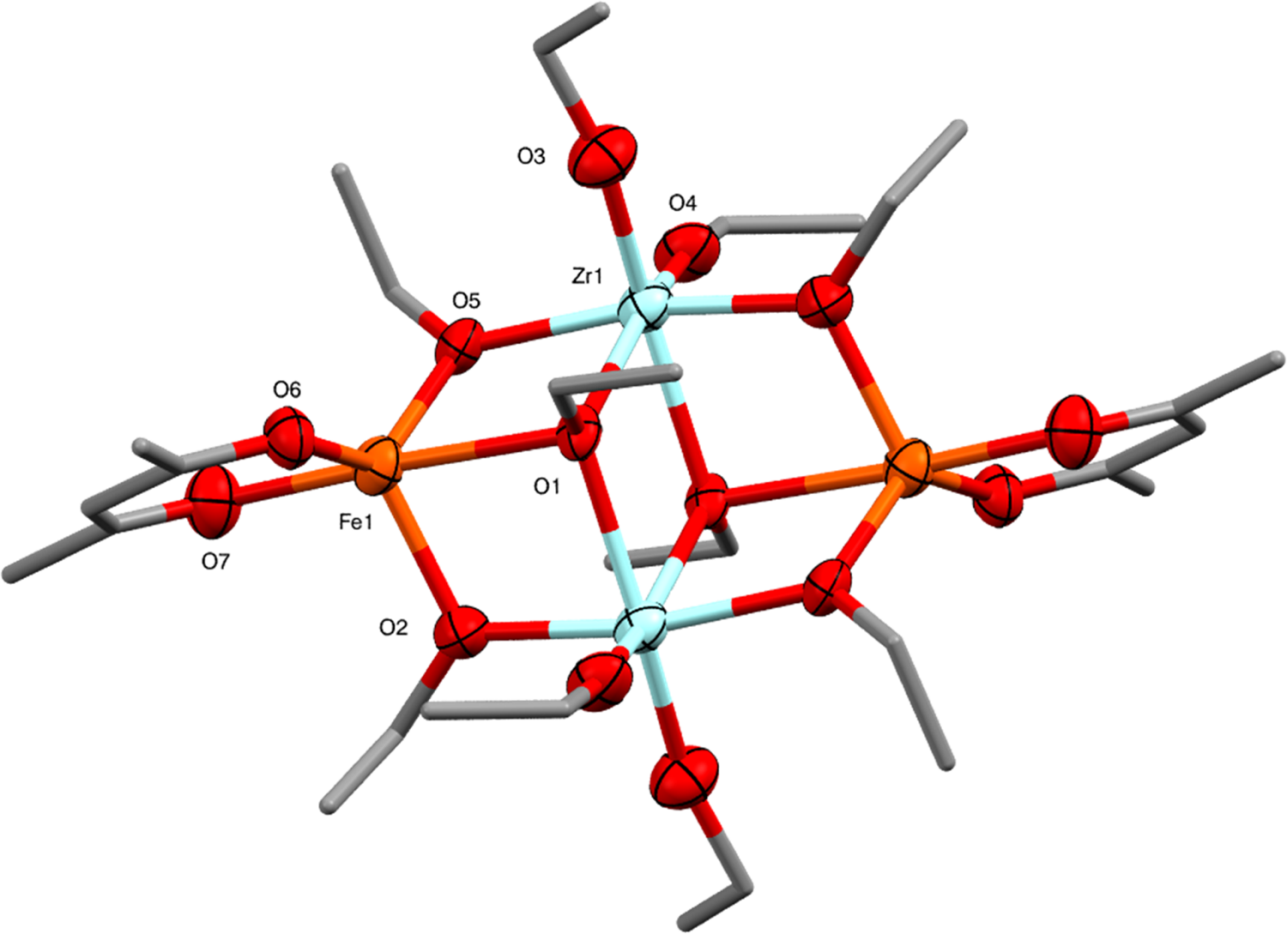
Molecular structure of Fe_2_Zr_2_(OEt)_10_(acac)_2_ (**5**) (ellipsoids at 50% probability) with H atoms omitted. One representative molecule from the asymmetric unit is shown. The other molecule has essentially identical conformation. Selected bond lengths (Å) and angles (°): Fe1–O1 2.419(7), Fe1–O2 2.030(9), Fe1–O5 1.989(8), Fe1–O6 1.945(9), Fe1–O7 2.029(10), Zr1–O1 2.250(8), Zr1–O2 2.098(9), Zr1–O3 1.926(8), Zr1–O4 1.915(8), Zr1–O5 2.129(8), O–Fe1–O 75.9(3)-130.3(4), O–Zr1–O 70.4(3)–103.9(4).

Rather than using the preformed metal acetylacetonate precursors themselves, it was found that the acetylacetonate ligands could also be incorporated into the mixed-metal complexes using an in situ approach. Heating a 1:1:2 stoichiometric mixture of Zr(OEt)_4_, FeCl_2_, and KOEt to reflux in toluene and ethanol, followed by the addition of one equivalent of acetylacetone (acacH) produced crystals of Fe_2_Zr_2_(OEt)_10_(acac)_2_ (**5**) after workup. If no acetylacetone is added to the reaction an intractable red residue is formed, presumably of Fe_2_Zr_2_(OEt)_12_. The addition of acetylacetone results in ligand substitution to form **5**. Molecules of **5** are based on the [{(EtO)_4_Zr(μ_2_-OEt)}_2_]^2–^ dianion and are isostructural with the Co^2+^ molecule **3**, with a distorted trigonal-bipyramidal coordination environment for the high-spin d^6^ Fe^2+^ ions and a distorted octahedral geometry for the zirconium ions (Figure 7). As for **3**, the presence of the outer trigonal-bipyramidal FeO_5_ units results in the greatest degree of distortion from regular octahedral geometry for the central ZrO_6_ sites. There have been a number of previous reports of iron-zirconium alkoxides, with the most similar being FeZr_3_O(O^n^Pr)_10_(acac)_3_.^34^

**Figure 8:**
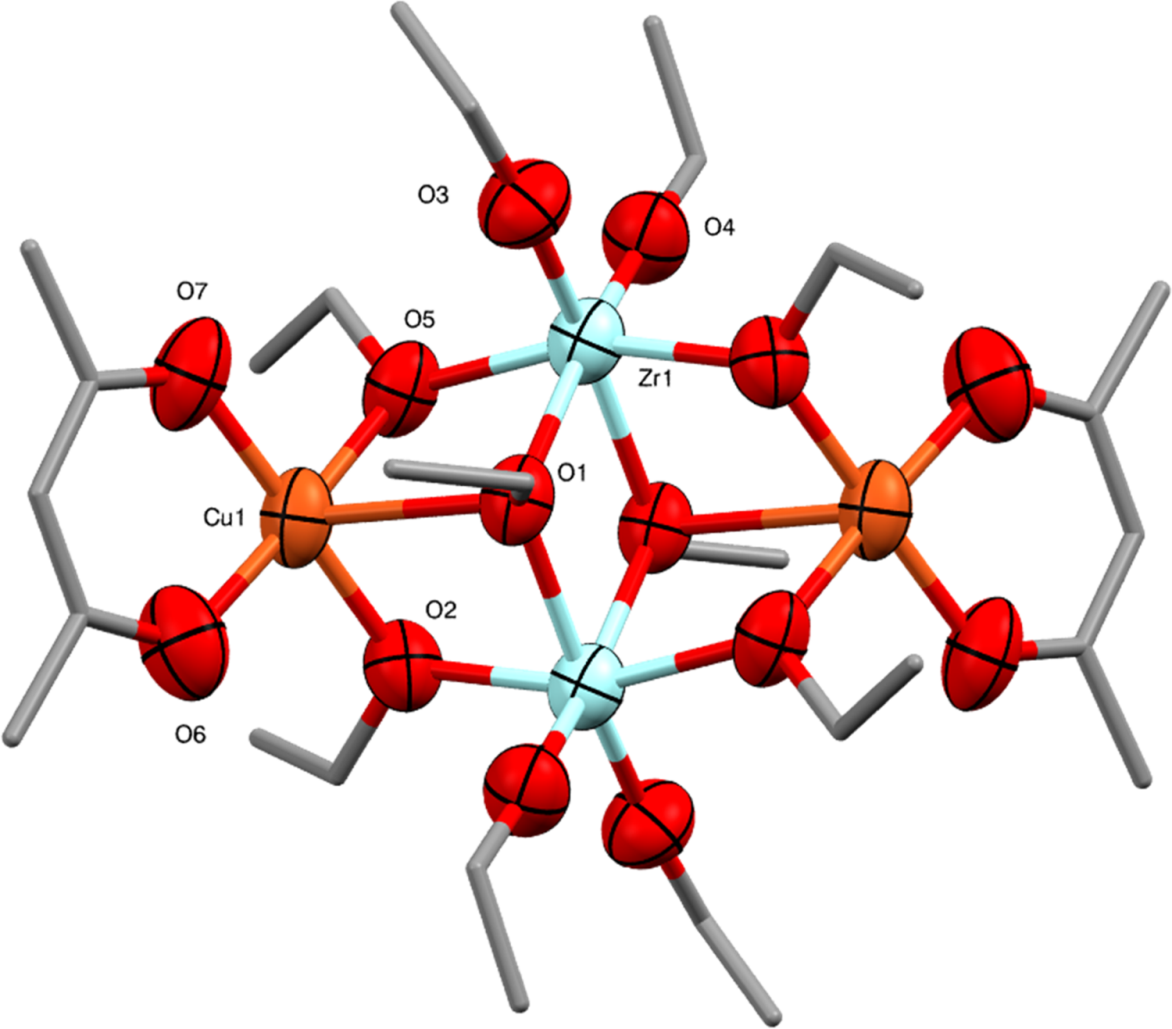
Molecular structure of Cu_2_Zr_2_(OEt)_10_(acac)_2_ (**6**) (ellipsoids at 50% probability) with H atoms and minor disorder of one ethoxide ligand omitted. Selected bond lengths (Å) and angles (°): Cu1–O1 2.543(3), Cu1–O2 1.959(4), Cu1–O5 1.964(4), Cu1–O6 1.914(5), Cu1–O7 1.918(4), Zr1–O1 2.212(4), Zr1–O1′ 2.205(4), Zr1–O2 2.116(4), Zr1–O3 1.936(4), Zr1–O4 1.933(4), Zr1–O5 2.131(4), O–Cu1–O 74.4(2)-111.3(2), O–Zr1–O 73.62(13)-101.0(2).

A very similar arrangement is also seen in the Cu^2+^ complex Cu_2_Zr_2_(OEt)_10_(acac)_2_ (**6**) (Figure 8), which was obtained from the reaction of Zr(OEt)_4_, Cu(acac)_2_, CuCl_2_, and KOEt in a 2:1:1:2 stoichiometry heated to reflux in toluene and ethanol. While **6** (like **3** and **5**) contains the [{(EtO)_4_Zr(μ_2_-OEt)}_2_]^2–^ dianion, here the d^9^ Cu^2+^ ions have a distorted square-based pyramidal geometry, with the base formed from coordination by two μ_2_-ethoxides and one chelating acetylacetonate ligand. In addition to these bonds, there is a long Cu···O interaction with a μ_3_-ethoxide (2.543(3) Å, cf. 1.959(4) and 1.964(4) Å for μ_2_-ethoxides). From the polyhedral perspective, each outer CuO_5_ square-based pyramid joins the central pair of octahedra, again through one triangular face. The adoption of a distorted square pyramidal geometry for Cu^2+^ containing weaker axial interactions has been seen previously in a number of other Cu^2+^ complexes.^35,36^

The adoption of two structural types, both composed of similar M_2_Zr_2_O_6_ cores but with the coordinated metal ions (M^n+^) in different peripheral (**1, 3**, **5**, and **6**) or central (**2** and **4**) positions (type 1 and type 2, Figure 4), suggests that a combination of factors, such as ionic radii, ionic charge, and crystal field stabilization, influences the observed molecular framework. For example, while there is only a small difference in ionic radii between Li^+^ (in **1**) and Mg^2+^ (in **2**) (both ≈ 90 pm for octahedral coordination),^37^ the key difference is the charge and charge density of the ions. The result is a preference for Li^+^ to adopt a four-coordinate pseudo-tetrahedral geometry while octahedral geometries are far more common for Mg^2+^ (i.e., maximizing bond enthalpy). An additional effect underlying the behavior of the transition-metal ions is the difference between crystal field stabilization energies (CFSE) for the various potential coordination environments. It is clear from a consideration of CFSE geometries for the ions (see Table 1) that in the presence of weak-field ligands such as alkoxides, Ni^2+^ has the greatest preference for octahedral geometry (and is found in the central octahedral M site), while Cu^2+^ will prefer square pyramidal. For Fe^2+^ and Co^2+^, while square pyramidal has the largest CFSE, the trigonal-bipyramidal geometry is observed. This presumably reflects the geometrical match between the alternative coordination polyhedra and the central edge-sharing octahedra.

**Table 1:** Calculated Crystal Field Stabilization Energies (CFSE) for Octahedral, Tetrahedral, Square Pyramidal, and Trigonal-Bipyramidal Geometries of the High-Spin Metal Ions, and the Observed Structure and Ionic Radius of the Ion

	CFSE					
	octahedral	tetrahedral	square pyramidal	trigonal-bipyramidal	observed structure	ionic radius (pm)
**1** Li^+^ (d^0^)	0	0	0	0	type 1 (tet)	90
**2** Mg^2+^ (d^0^)	0	0	0	0	type 2 (oct)	86
**3** Co^2+^ (d^7^)	–0.8Δ_o_	–0.53Δ_o_	–0.91Δ_o_	–0.54Δ_o_	type 1 (tbp)	88.5
**4** Ni^2+^ (d^8^)	–1.2Δ_o_	–0.36Δ_o_	–1.00Δ_o_	–0.63Δ_o_	type 2 (oct)	83
**5** Fe^2+^ (d^6^)	–0.4Δ_o_	–0.27Δ_o_	–0.46Δ_o_	–0.27Δ_o_	type 1 (tbp)	92
**6** Cu^2+^ (d^9^)	–0.6Δ_o_	–0.18Δ_o_	–0.91Δ_o_	–0.71Δ_o_	type 1 (sbp)	87
**7** Mn^2+^ (d^5^)	0	0	0	0	mixed-metal sites	97
**8** Zn^2+^ (d^10^)	0	0	0	0	type 1 (tet)	88

**Figure 9:**
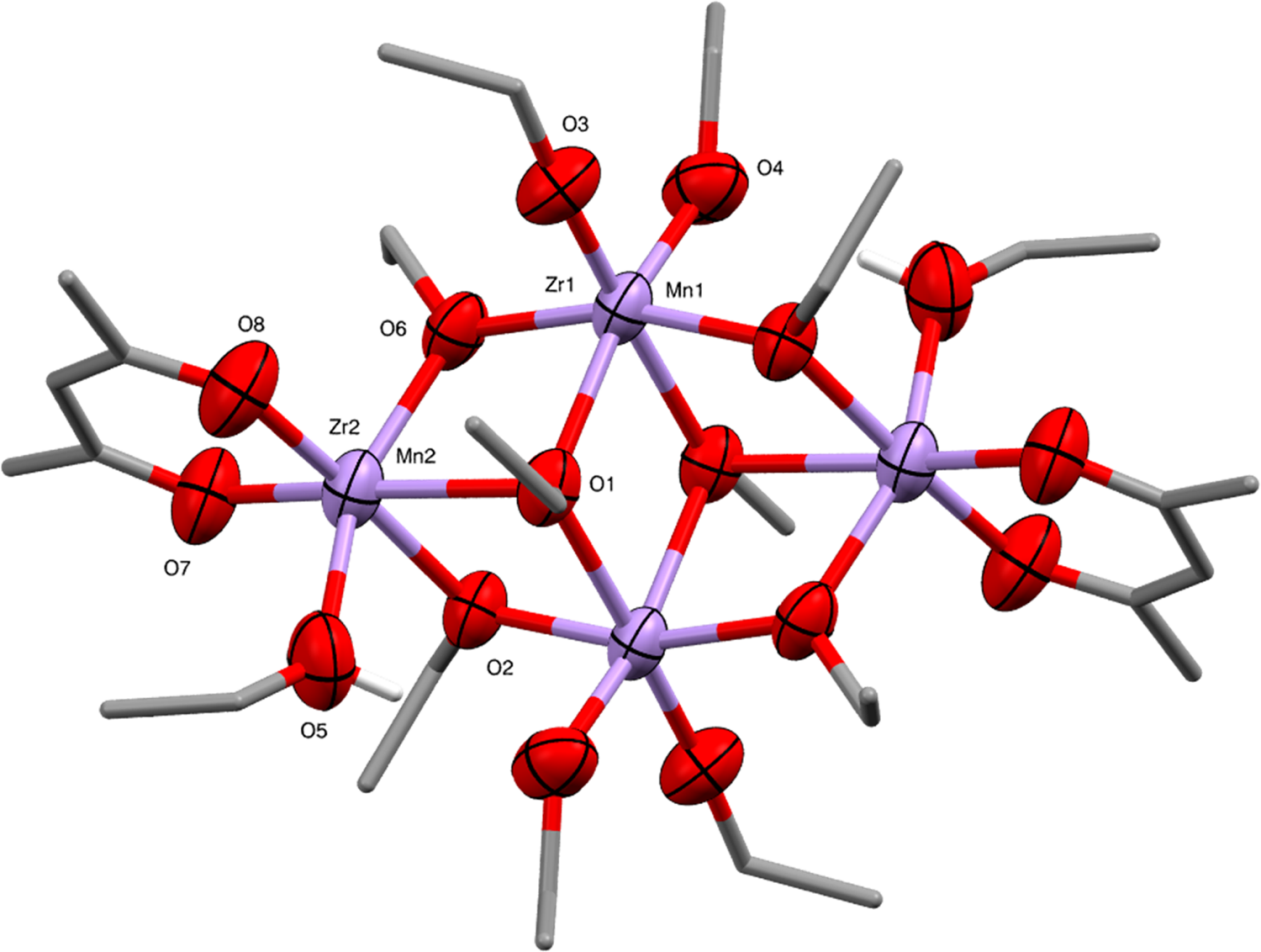
Molecular structure of Mn_1.67_Zr_2.33_(OEt)_10.66_(acac)_2_(EtOH)_1.34_ (**7**) (ellipsoids at 50% probability) with H atoms and minor disorder of one ethoxide ligand omitted. The O–H hydrogens of coordinating alcohols are shown. In the crystal examined, the metal position M1 has 9% Mn^2+^ occupancy and 91% Zr^4+^ occupancy, and the metal position M2 has 74% Mn^2+^ occupancy and 26% Zr^4+^ occupancy. Selected bond lengths (Å) and angles (°): M1–O1 2.262(3), M1–O1′ 2.290(3), M1–O2 2.087(3), M1–O3 1.924(4), M1–O4 2.014(4), M1–O6 2.085(4), M2–O1 2.407(3), M2–O2 2.172(3), M2–O5 2.199(5), M2–O6 2.163(4), M2–O7 2.088(4), M2–O8 2.046(4), O–M1–O 74.76(13)–101.96(15), O–M2–O 73.62(12)–101.77(18).

An indication of the importance of both ionic radius and CFSE is seen in the crystal structure of the Mn^2+^ complex Mn_1.67_Zr_2.33_(OEt)_10.66_(acac)_2_(EtOH)_1.34_ (**7**), obtained from the 1:1:2 stoichiometric reaction of Zr(OEt)_4_, MnCl_2_, and KOEt heated to reflux in *n*-hexane and ethanol, followed by the addition of one equivalent of acetylacetone. Complex **7** could not be obtained from the reaction of Zr(OEt)_4_ with Mn(acac)_2_ alone. The crystal structure of **7** (Figure 9) indicates that the Mn^2+^ and Zr^4+^ ions show site disorder with the peripheral metal positions having 74% Mn^2+^ occupancy and 26% Zr^4+^ occupancy, and the central metal positions have 9% Mn^2+^ occupancy and 91% Zr^4+^ occupancy in the crystal examined. The disorder of the metals in **7** was investigated using ICP-OES to measure the Mn and Zr content. For the formula Mn_1.67_Zr_2.33_(OEt)_10.66_(acac)_2_(EtOH)_1.34_, determined from X-ray analysis, the calculated values are 8.8% Mn and 20.4% Zr. The values found experimentally by ICP-OES are 10.4% Mn and 20.0% Zr, showing a greater amount of Mn in the bulk than in the crystallographic model (on a single crystal). The partial occupancy of manganese and zirconium also results in a mixture of ethoxide and ethanol ligands being present in the crystal structure for charge balance. In effect, the complex is part way between the type 1 and 2 arrangements, and this is a reflection of the zero CFSE of the high-spin d^5^ electronic configuration of Mn^2+^ in an octahedral (or indeed any) coordination geometry (see Figure 4). Quantitative measures (see Table S2) show that both the M and M′ octahedra in **7** show polyhedral volumes larger than any octahedra in the other complexes, and the M′ site shows the greatest degree of distortion from regular octahedral geometry. This particularly distorted geometry of **7** coincides with the greatest mismatch in ionic radius for Mn^2+^ (97 pm) and Zr^4+^ (86 pm).^37^ There are few examples of structurally characterized manganese-zirconium alkoxide, with only two previous examples, and these contained either chloride or nitrogen donor ligands.^38,39^ Similar to **2**, the presence of alcohol ligands was investigated using IR spectroscopy. The IR spectrum (Figure S16) has a weak broad signal at about 3100 cm^–1^ attributed to the coordinating alcohol ligands.

**Figure 10:**
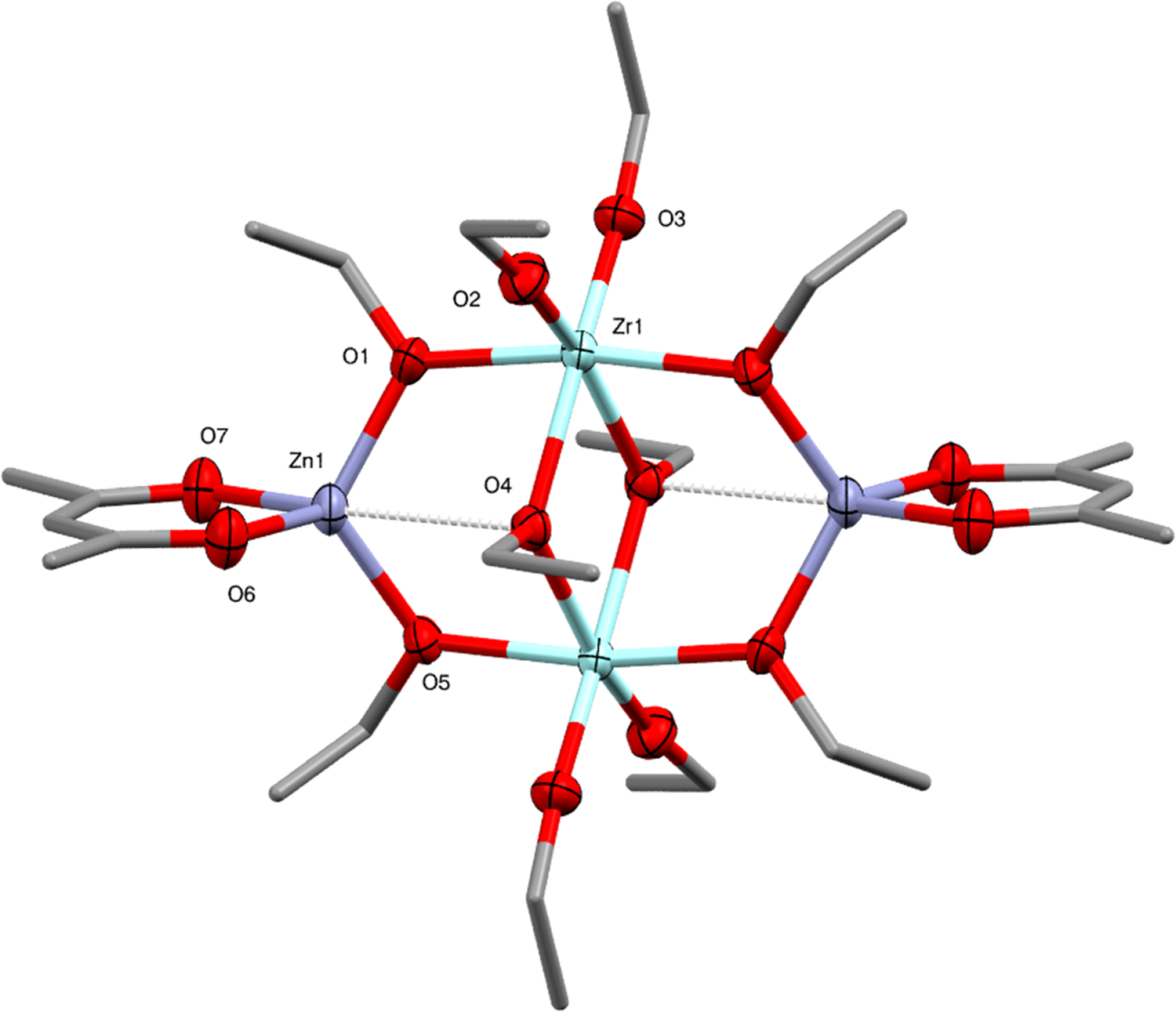
Molecular structure of Zn_2_Zr_2_(OEt)_10_(acac)_2_ (**8**) (ellipsoids at 50% probability) with H atoms omitted. Selected bond lengths (Å) and angles (°): Zn1–O1 1.9300(13), Zn1–O4 2.8013(12), Zn1–O5 1.9354(12), Zn1–O6 1.9596(13), Zn1–O7 1.9851(13), Zr1–O1 2.1401(12), Zr1–O2 1.9358(13), Zr1–O3 1.9396(13), Zr1–O4 2.2001(11), Zr1–O4′ 2.1950(12), Zr1–O5 2.1374(12), O–Zn1–O 95.32(6)-116.59(6), O–Zr1–O 69.73(5)-101.78(6).

In the Zn^2+^complex Zn_2_Zr_2_(OEt)_10_(acac)_2_ (**8**) (obtained in a similar synthesis to **7**), steric effects and the relatively small ionic radius of Zn^2+^ are the main structure-directing influences as the d^10^ configuration has no CFSE (see Table 1). Like complexes **1**, **3**, **5**, and **6**, complex **8** is probably best formulated as a [{(EtO)_4_Zr(μ_2_-OEt)}_2_]^2–^ dianion which (in this case) coordinates two [Zn(acac)]^+^ fragments at the periphery (i.e., type 1) (Figure 10). Although the coordination geometry of Zn^2+^ appears to be typical four-coordinate, pseudo-tetrahedral, the geometry of **8** actually resembles most closely that of **3** and **5**, which show trigonal-bipyramidal coordination for the M′ site. The additional “axial” contact to atom O4 (2.8013(12) Å, Figure 10) in **8** is long, but it corresponds to bonds seen in **3**, **5**, and **1** (the orientation of the apparent tetrahedron in **8** is quite different from that in **1**). This is the first example of a structurally characterized zinc-zirconium alkoxide not containing Zr–C or Zr–Cl bonds, which are both very air sensitive. In contrast to the other diamagnetic species (**1** and **2**), the room-temperature ^1^H NMR spectrum of **8** (Figure S6) matches well to the solid-state structure: there are three distinct ethoxide environments in a 2:2:1 ratio and a single acetylacetonate environment.

**Figure 11:**
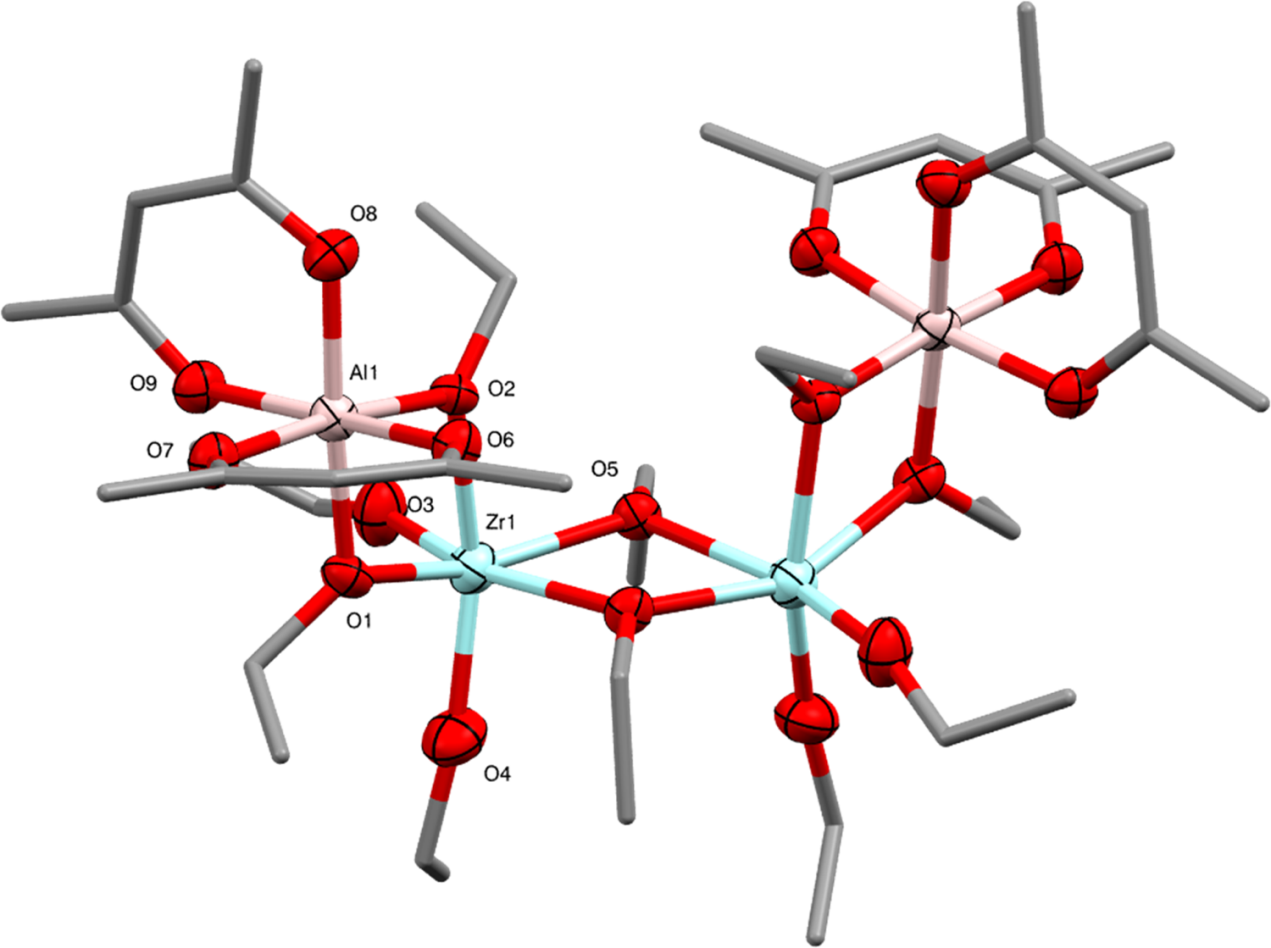
Molecular structure of Al_2_Zr_2_(OEt)_10_(acac)_4_ (**9**) (ellipsoids at 50% probability) with H atoms omitted. Selected bond lengths (Å) and angles (°): Al1–O1 1.885(4), Al1–O2 1.864(4), Al1–O6 1.915(4), Al1–O7 1.906(4), Al1–O8 1.874(5), Al1–O9 1.906(4), Zr1–O1 2.109(4), Zr1–O2 2.161(3), Zr1–O3 1.901(4), Zr1–O4 1.905(5), Zr1–O5 2.105(4), Zr1–O5′ 2.192(4), O–Al1–O 79.10(17)–96.02(18), O–Zr1–O 67.99(15)–105.3(2).

Finally, moving into the p-block, the Al^3+^ complex Al_2_Zr_2_(OEt)_10_(acac)_4_ (**9**) was synthesized by the reaction of Zr(OEt)_4_, Al(OEt)_3_ and acetylacetone in a 1:1:2 stoichiometric ratio. X-ray crystallography revealed a significantly different arrangement to the previous examples (Figure 11). Its spirocyclic structure is composed of a central [Zr(μ-OEt)_2_Zr] ring connected to two terminal [Al(μ-OEt)_2_Zr] rings, and is probably best regarded as being composed of a [{(EtO)_4_Zr(μ_2_-OEt)}_2_]^2–^ dianion (similar to those in complexes **1**, **3**, **5**, **6**, and **8**) which coordinates two terminal [Al(acac)_2_]^+^ cation fragments. This gives the Zr^4+^ and Al^3+^ ions six-coordinate octahedral geometries. The greater distortion of the Zr^4+^ environment appears to result from the chelation of two of the ethoxide groups of the [{(EtO)_4_Zr(μ_2_-OEt)}_2_]^2–^ dianion on each of the Zr^4+^ ions to the two Al^3+^ ions. The ^1^H NMR spectrum of **9** (Figure S8) is very complex with a large number of signals, suggesting a number of species in solution at room temperature in dynamic equilibrium. The ^27^Al NMR spectrum (Figure S10) has two signals (ignoring the background signal) at δ 34.3 and 5.0 ppm, corresponding to five- and six-coordinate aluminum environments, respectively.^40^ The ^1^H NMR spectrum at −50 °C (Figure S13) was little changed from that at room temperature, suggesting that the dynamic processes occurring in solution have low activation energy.

Although Al_2_Zr(O^i^Pr)_10_ was reported previously, being characterized by mass spectrometry and elemental analysis, attempts to crystallize this only resulted in the formation of Al(O^i^Pr)_3_.^41^ However, the structures of Al_2_Ti(O^i^Pr)_10_ and Al_2_Hf(O^i^Pr)_10_ have been determined by X-ray crystallography.^41,42^ In addition, the aluminum-barium-zirconium alkoxide AlBaZr_2_(O^i^Pr)_13_(^i^PrOH) has also been reported.^43^ To the best of our knowledge, **9** is the first structurally characterized aluminum–zirconium complex containing only oxygen-based ligands. The absence of reactive Al–C or Al–Cl bonds in **9** which most of the previous examples of aluminum–zirconium complexes have contained makes it a potentially more easily handled SSP for aluminum–zirconium oxide phases.

UV–visible spectroscopy was employed to provide support for the assignment of the oxidation states of the complexes containing paramagnetic transition-metal ions (for which NMR spectroscopy was more challenging). This, however, proved to be relatively uninformative. Complexes **3**, **4**, and **6** all showed d–d transitions in their spectra corresponding to transitions of the M^2+^ ions (Figures S17, S18, and S20). For complex **5** there are no apparent peaks from d–d transitions (Figure S19), which is unexpected for a high-spin d^6^ complex. However, similar behavior has been observed before in comparable iron-titanium alkoxides.^44^ The UV–visible spectrum of **7** (Figure S21) does not have any d–d transitions as the Mn^2+^ is high-spin d^5^. All of the acac complexes have strong absorption at 400 nm which is assigned to a d to π transition from the metal to the acetylacetonate.^45^

### Thermal Decomposition Studies

**Figure 12:**
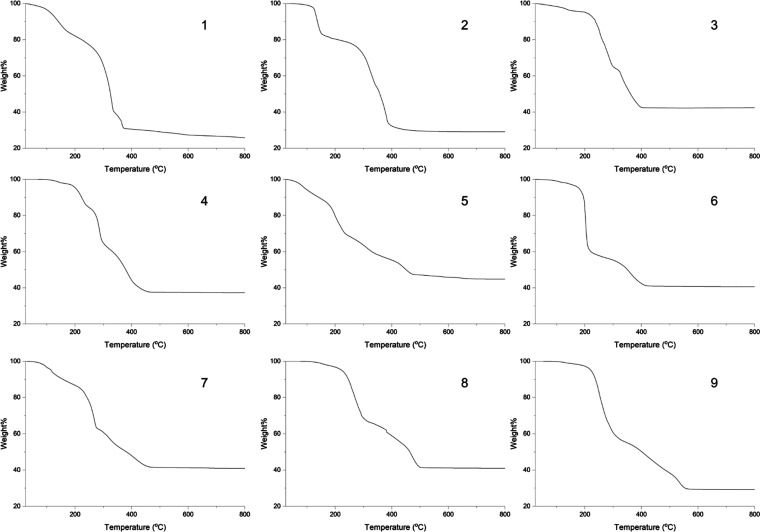
TGA plots of complexes **1**–**9** heated from 25 to 800 °C in air.

The thermal decomposition pathways of all of the new compounds were initially tested with thermogravimetric analysis (TGA). For TGA, the samples were heated to 800 °C in air and the weight of the samples was recorded throughout. The TGA traces are shown in Figure 12. Table 2 summarizes the experimental and predicted weight losses.

**Table 2:** Experimental and Predicted Weights from the Thermal Decomposition of Complexes **1**–**9** at 800 °C and the Predicted Products

	remaining weight (%)		
complex	experimental	predicted	predicted product
**1**	25.8	29.7	Li_2_Zr_2_O_5_
**2**	29.0	27.7	Mg_2_Zr_2_O_6_
**3**	42.4	41.8	Co_2_Zr_2_O_6_
**4**	37.3	37.5	Ni_2_Zr_2_O_6_
**5**	44.8	41.4	Fe_2_Zr_2_O_6_
**6**	40.6	42.3	Cu_2_Zr_2_O_6_
**7**	40.9	38.8	Mn_1.67_Zr_2.33_O_6.33_
**8**	41.0	42.5	Zn_2_Zr_2_O_6_
**9**	29.2	32.2	Al_2_Zr_2_O_7_

The thermal decomposition of **1** occurs in two steps with an initial weight loss occurring at 100–200 °C, attributed to the loss of the THF ligands, followed by a second weight loss event at 200–380 °C ascribed to the loss of the *n*-propoxide ligands to leave Li_2_Zr_2_O_5_ (experimental remaining weight 30.8%, predicted remaining weight 29.7%). However, continued heating of the material results in a further gradual loss of material, with a final weight of 25.8%. This additional loss is tentatively attributed to the loss of lithium oxide at higher temperatures due to its volatility, resulting in the formation of ZrO_2_ (predicted remaining weight 26.5%).^46,47^ The behavior of **2** is similar, with a first weight loss of 20% at 120–180 °C, attributed to the loss of four *n*-propanol ligands to give Mg_2_Zr_2_(O^n^Pr)_12_, followed by a second weight loss event at 200–450 °C which is due to the loss of the remaining organic ligands to leave Mg_2_Zr_2_O_6_ (experimental remaining weight 29.0%, predicted remaining weight 27.7%).

The thermal decomposition of **3** occurs in one combined step with the main loss occurring from 200 to 400 °C. The overall 57.6% weight loss is attributed to the loss of the organic ligands and the formation of Co_2_Zr_2_O_6_ (experimental remaining weight 42.4%, predicted remaining weight 41.8%). The TGA trace of **4** has a complicated shape, with multiple overlapping weight loss events. Full decomposition is achieved at 470 °C, producing Ni_2_Zr_2_O_6_ (experimental remaining weight 37.3%, predicted remaining weight 37.5%).

The thermal decomposition of **5** is also complicated. The TGA trace shows at least four weight loss events, with a gradual decrease in weight being observed from room temperature to 700 °C. During loading of the sample, it was noted that **5**, which is red, rapidly turned green upon air exposure, which was attributed to the oxidation of the Fe^2+^ ions to Fe^3+^. This poor air stability may be partially responsible for the complicated TGA trace. The resulting final weight matches Fe_2_Zr_2_O_7_, which corroborates oxidation of Fe^2+^ to Fe^3+^ (experimental remaining weight 44.8%, predicted remaining weight Fe_2_Zr_2_O_6_ 41.4%, predicted remaining weight for Fe_2_Zr_2_O_7_ 43.1%).

Like **1** and **2**, the thermal decomposition of **6** occurs in two distinct stages. First, a very sharp weight loss of 42% at 200–220 °C, which is attributed to the loss of the ethoxide ligands, and a second stage occurring at 250–420 °C due to the removal of the acetylacetonate ligands to give Cu_2_Zr_2_O_6_ (experimental remaining weight 40.6%, predicted remaining weight 42.3%).

A similar picture to **5** is seen in the TGA trace **7**, which is attributed to the more complicated mixed-ligand set and fractional metal composition, with multiple weight loss events occurring between 100 and 470 °C. Based on the formula obtained by X-ray crystallography (Mn_1.67_Zr_2.33_(OEt)_10.66_(acac)_2_(EtOH)_1.34_) the expected decomposition product would be Mn_1.67_Zr_2.33_O_6.33_ (experimental remaining weight 40.9%, predicted remaining weight 38.8%). The difference between the predicted and observed remaining weights is probably due to variation in the exact Mn:Zr ratio in **7** (i.e., between different crystals in the bulk sample). This discrepancy was confirmed by performing Mn and Zr ICP-OES on **7**.

A two-stage weight loss is observed for **8**. The first weight loss occurs at 150–320 °C and the second at 350–500 °C. These two stages are again attributed to the loss of ethoxide and acetylacetonate ligands, respectively. The final decomposition product has the formula Zn_2_Zr_2_O_6_ (experimental remaining weight 41.0%, predicted remaining weight 42.5%).

The thermal decomposition of **9** also has a two-step TGA profile. The first step occurs at 200–320 °C, with the second step occurring at 320–570 °C. Again, these two steps can be linked to the loss of ethoxide and acetylacetonate ligands. This leads to a final composition of Al_2_Zr_2_O_7_ (experimental remaining weight 29.2%, predicted remaining weight 32.2%).

### Analysis of Decomposition Products

To characterize the products from thermal decomposition, we heated each of the complexes in air at 800 °C for 4 h and performed synchrotron powder X-ray diffraction (PXRD) measurements at the I11 beamline at Diamond Light Source. Detailed composition on analysis was undertaken using Rietveld refinements. The complexes variously phase-segregate into ZrO_2_ and the relevant metal oxide and form a mixed-metal phase or a combination of both. The results are summarized in Table 3; refined lattice parameters and phase weight percentages are included in the SI (Table S4 and Figures S22–S30).

**Table 3:** Summary of the Weight% and Errors of Phases Present from PXRD Rietveld Refinements for Each Decomposition Product after Heating at 800 °C for 4 h in Air (Complex **9** Was Heated to 1000 °C for 4 h in Air)^a^

	phase composition (weight%)				
complex	monoclinic ZrO_2_	tetragonal M_*x*_Zr_1–*x*_O_2−δ_	cubic M_*x*_Zr_1–*x*_O_2−δ_	M phase	other mixed phase
**1**	52.7(3)				47.3(3) Li_2_ZrO_3_
**2**		54.7(14)	26.1(13)	19.2(6) MgO	
**3**	18.28(13)	45.0(3)		36.75(19) Co_3_O_4_	
**4**	10.15(19)	45.9(3)		43.9(2) NiO	
**5**	12.6(8)	69.5(18)		17.9(11) Fe_2_O_3_	
**6**	57.8(7)	1.2(11)		41.0(5) CuO	
**7**		a	a	aMn_3_O_4_	
**8**	60.20(10)			39.80(10) ZnO	
**9**		87.9(16)		12.1(16) Al_2_O_3_	

a For complex 7, tetragonal zirconia, cubic zirconia, and Mn_3_O_4_ are observed, but the composition and structure are unsolved.

Zirconia (ZrO_2_) is thermodynamically stable in its monoclinic form (space group **P**2_1_/**c**) below 1170 °C. It is tetragonal (**P**4_2_/**nmc**) from 1170 to 2300 °C and cubic (*Fm*3*®m*) above this temperature; zirconium exists in an oxidation state of +4 and forms strong bonds to oxygen, which favors a coordination number of seven or eight. The tetragonal and cubic phases can instead be stabilized at lower temperatures by doping with lower valent ions, which also introduces oxygen vacancies for charge-balancing; yttrium-stabilized cubic zirconia is perhaps the most well known of these compounds.^48−53^ Alternatively, when crystallites are below a critical size (found by Shukla et al. to be 30 nm) the high surface area to volume ratio means that oxygen loss at the surface can lead to a sufficient oxygen vacancy concentration to stabilize the tetragonal zirconia phase.^54−56^ By choosing the right counterion, we can therefore carefully control the nature of the oxide phases and tailor the lattice parameters and/or oxygen vacancy content to control, e.g., strain/ionic conductivity.

The decomposition products of the Cu (**6**) and Zn (**8**) complexes phase-segregate into monoclinic ZrO_2_ and oxide phases of CuO and ZnO, respectively. The preferred coordination is a Jahn–Teller distorted octahedron for d^9^ Cu^2+^ and tetrahedral for small d^10^ Zn^2+^ so we can attribute the lack of metal doping in these cases to the very different coordination preferences of the Cu^2+^ and Zn^2+^ ions relative to Zr^4+^.

**Figure 13:**
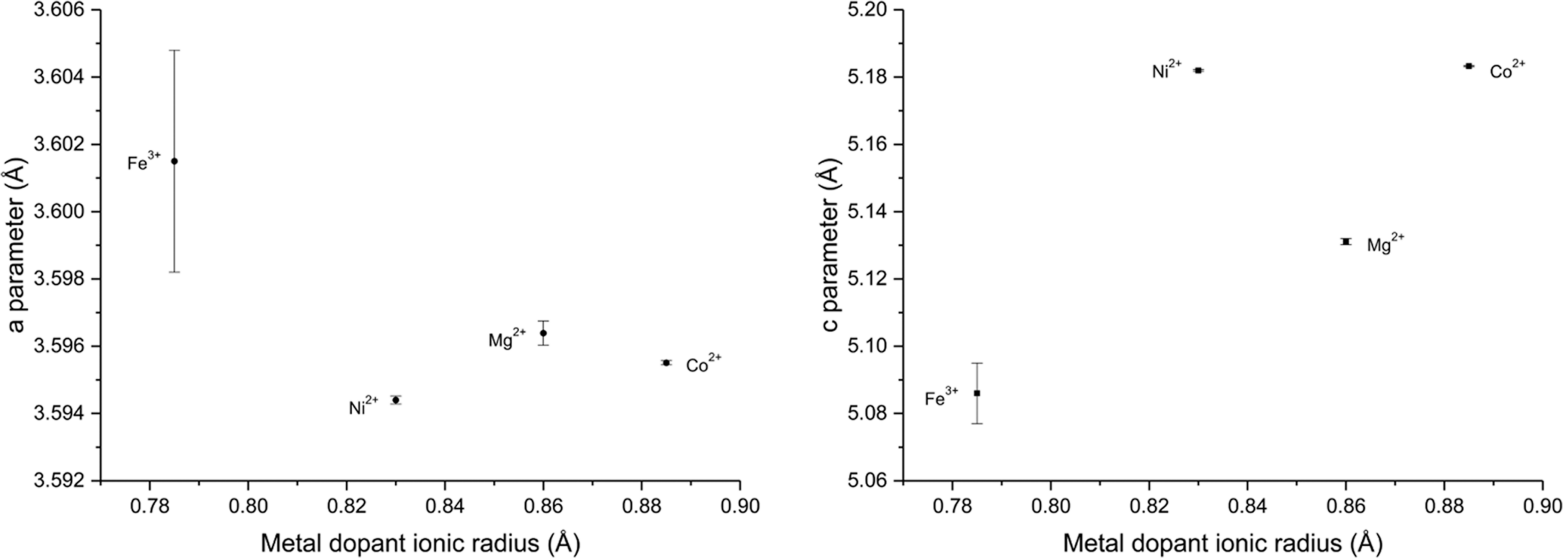
*a* and *c* Parameters with errors for the tetragonal zirconia phases calculated from Rietveld refinement of the PXRD data from the decomposition of the complexes at 800 °C in air for 4 h plotted against the metal dopant ionic radius.

The Co (**3**), Ni (**4**), and Fe (**5**) complexes form a doped tetragonal phase with additional monoclinic ZrO_2_ and their respective oxide phases Co_3_O_4_, NiO and Fe_2_O_3_. This corroborates the TGA data, with some of the Co^2+^ and Fe^2+^ ions being oxidized during heating. The unit cell parameters were correlated with the ionic radii of the metal dopant; the variations of the refined *a* and *c* lattice parameters are shown in Figure 13. There is little change in the *a* lattice parameter for the different metal dopants (0.0071 Å). However, the *c* parameter has a much larger variation (0.09725 Å) and there is a weak positive correlation between the metal dopant ionic radius and the *c* parameter, suggesting doping does affect the *c* parameter. It must be noted that the amount of transition-metal dopant in the tetragonal zirconia does vary for the different dopants, so the absolute change in lattice parameters should only be tentatively analyzed. However, controlling the lattice parameter via doping could be an effective strategy for strain-matching between coatings and electrode materials in any future applications in the coating of battery material.

The Mg complex (**2**) forms a mixture of cubic and tetragonal Mg-doped ZrO_2_ and MgO. Due to the similar ionic radii of Mg^2+^ and Zr^4+^ (both ≈ 0.86 Å) and lack of CFSE for both ions, Mg^2+^ ions can easily dope into the zirconia phase, providing significant stabilization for both the cubic and tetragonal phases.^57^ The cubic zirconia also receives additional stabilization from forming a solid solution with MgO.^58,59^ ZrO_2_-MgO nanocomposites are of interest for their high melting points and excellent mechanical properties.^60^

The Mn complex (**7**) forms a mixture of Mn-doped tetragonal ZrO_2_, small amounts of tetragonal Mn_3_O_4_ (**I**4_1_/*amd*), and an additional cubic phase. This cubic phase could be fitted using numerous structural models which do not correspond to known phases containing only Mn and/or Zr with oxygen, so we refine this phase using a Pawley fit with space group *Fm*3̅*m*. There are also some additional unidentified peaks in the diffraction pattern for which further analysis is required. For the scope of this study, we note simply that the lack of CFSE for Mn^2+^ and Zr^4+^ allows for cation mixing in the decomposition product, analogous to that seen for the molecular structure of **7**.

The refinement of the PXRD data for complex **1** reveals 58% monoclinic Li_2_ZrO_3_ and 42% monoclinic ZrO_2_. This is the only compound to form a non-ZrO_2_ based mixed-metal phase.

The thermal decomposition of the Al-containing complex (**9**) at 800 °C resulted in an amorphous product, confirmed by the absence of sharp reflections in the XRD data. This was confirmed by magic-angle spinning ^27^Al solid-state NMR spectroscopy (Figure S14). The spectrum reveals four-, five-, and six-coordinate aluminum environments similar to that seen for amorphous alumina.^61^ This is consistent with previous reactions with alumina-zirconia ceramics, with amorphous samples observed upon heating at 600 °C, but γ-alumina and tetragonal zirconia are observed at 875 °C.^62^ Heating to higher temperatures would be required to observe crystalline phases; the increase in the crystallization temperature for zirconia with increasing alumina content has previously been studied.^63^ This was evidenced by heating **9** to 1000 °C for 4 h in air, resulting in a mixture of Al_2_O_3_ and tetragonal ZrO_2_ (Figure S31).

The decomposition products of the SSPs were further examined using scanning electron microscopy (SEM) and energy-dispersive X-ray spectroscopy (EDS) to investigate the particle morphology and elemental distribution of the materials heated at 800 °C in air for 4 h. The SEM images reveal a range of different morphologies for the different decomposition products, with most of them forming chunks of material. The EDS maps reveal a uniform distribution of elements in all of the decomposition products, suggesting good mixing of the different phases on the SEM length scale (see Figure S32). This is likely to be due to indistinguishable nanometer-size crystallites, supported by the PXRD refinements, which found crystallite sizes for all of the phases to be in the range of 30–150 nm. This uniform mixing is an important characteristic of SSPs.

## Conclusions

Using a set of similar synthetic methodologies, we have prepared a range of mixed-metal zirconium alkoxide compounds that incorporate metal ions and span the s-, d-, and p-blocks. The study of their solid-state structures has emphasized that the characteristics and coordination preferences of the incorporated metal ions in these zirconium “host” arrangements have a large structure-directing influence. While this conclusion is not new, because of the breadth of these studies we are able to trace the influences of the combined effects of ionic radii, ionic charge, and crystal field stabilization energy across the periodic table. By obtaining a series of these mixed-metal compounds this study provides a range of SSPs for the deposition of a number of metal-zirconium oxide, metal oxide, and zirconium oxide phases. The additional metal ion has a large effect on the zirconia phases formed, being able to stabilize the less thermodynamically stable tetragonal and cubic phases at moderate annealing temperatures. This knowledge may allow the future design of SSPs for specific purposes, where particular zirconia phases are desired. These species have the potential to be used in coatings, in particular the coating of battery electrodes to increase cycle lifetime by slowing or preventing degradation. We are continuing these studies by exploring the applications of these and the developing range of zirconium-based SSPs as protective coatings for state-of-the-art cathode materials.
