# Supplementary material for: Synthesis of Heterometallic Zirconium Alkoxide Single-Source Precursors for Bimetallic Oxide Deposition
Source: Inorg Chem. 2022 Nov 16;61(48):19203–19. doi: 10.1021/acs.inorgchem.2c02852 (PMC9727732; doi:10.1021/acs.inorgchem.2c02852)
Supplement: Supplementary file 1 — ic2c02852_si_001.pdf [file ic2c02852_si_001.pdf]

Supplementary Information

The synthesis of heterometallic zirconium alkoxide single-source precursors for bimetallic oxide deposition

Jonathan Slaughter,[1,2] Chloe Coates,[1,2] George Phillips,[1] Dipanjana Choudhury,[1] Andrew D. Bond,[1] Clare P. Grey*,[1,2] and Dominic S. Wright*,[1,2]

[1] Yusuf Hamied Department of Chemistry, University of Cambridge, Lensfield Road, Cambridge, CB2 1EW, United Kingdom

[2] The Faraday Institution, Quad One, Harwell Science and Innovation Campus, Didcot, OX11 0RA, United Kingdom

corresponding author's email address: dsw1000@cam.ac.uk

Contents

1. Synthesis 2
2. Crystallography Data 3
3. NMR Spectroscopy 8
4. IR Spectroscopy 15
5. UV-visible Spectroscopy 17
6. PXRD 22
7. SEM 28

1. Synthesis

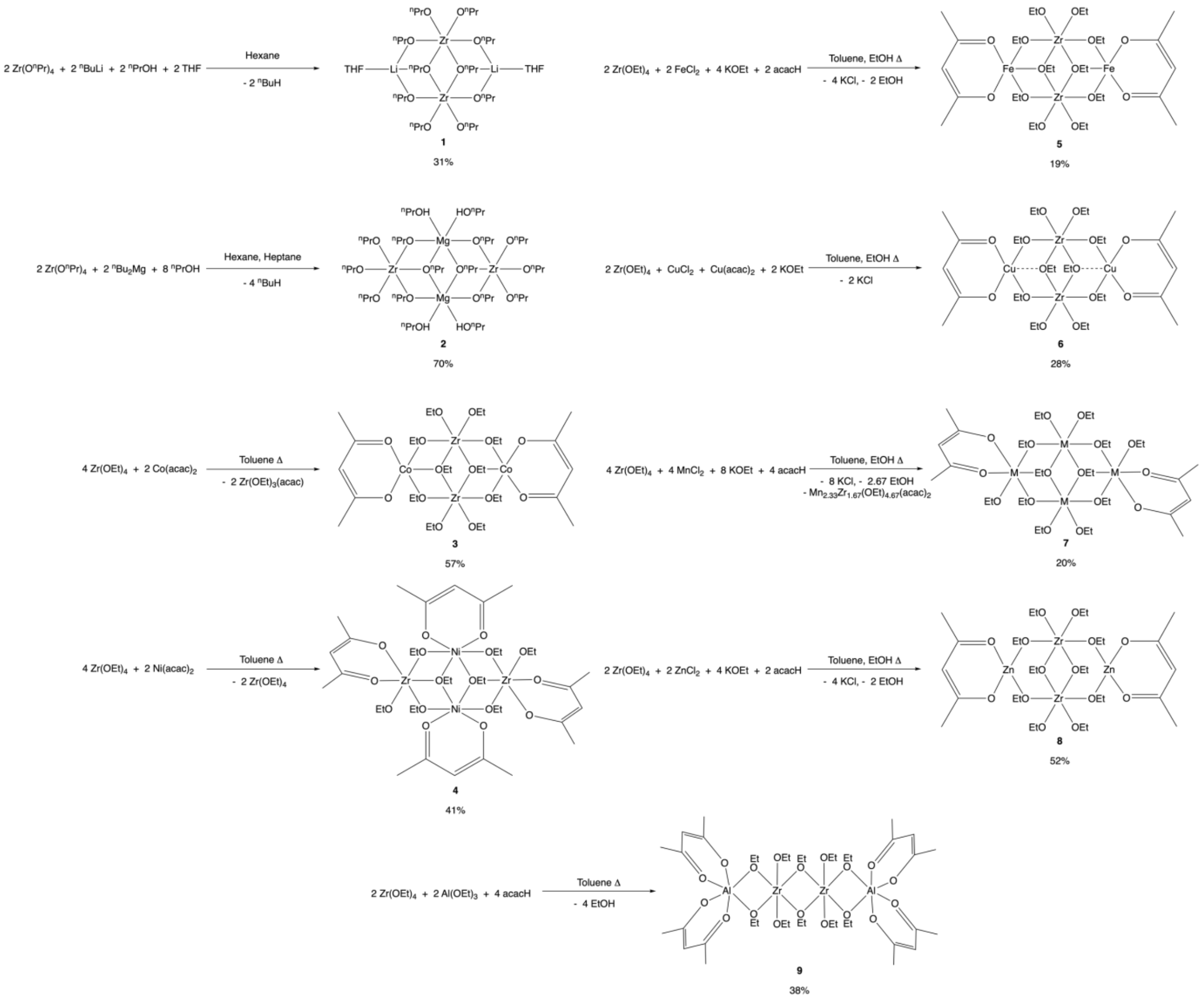

Scheme S1: The synthesis of complexes **1**-**9**.

2. Crystallography Data

Table S1: X-ray data for complexes **1**-**9**.

	**1**	**2**	**3**	**4**	**5**
CCDC number	2185397	2185400	2185402	2185404	2185401
Cambridge data number	DW_K1_0033	DW_B1_0369	DW_B1_0404	DW_B2_0346	DW_B1_0405
Chemical formula	$C_{38}H_{86}Li_2O_{12}Zr_2$	$C_{48}H_{116}Mg_2O_{16}Zr_2$	$C_{30}H_{64}Co_2O_{14}Zr_2$	$C_{36}H_{68}Ni_2O_{16}Zr_2$	$C_{30}H_{64}Fe_2O_{14}Zr_2$
Formula weight	931.38	1180.46	949.11	1056.76	942.95
Temperature / K	180(2)	180(2)	180(2)	180(2)	180(2)
Crystal system	triclinic	orthorhombic	monoclinic	triclinic	triclinic
Space group	P -1	C m c a	P 21/c	P -1	P -1
a / Å	10.5368(2)	21.4340(6)	39.0205(11)	9.3348(4)	8.9792(5)
b / Å	11.9008(4)	14.3039(4)	8.8776(3)	11.3803(4)	11.4919(6)
c / Å	11.9957(4)	21.4961(7)	24.9673(7)	12.0068(5)	21.4254(11)
alpha / °	100.9973(14)	90	90	100.8055(13)	89.484(3)
beta / °	110.6464(15)	90	99.355(2)	104.4765(13)	84.857(3)
gamma / °	106.8803(15)	90	90	91.6192(14)	75.941(3)
Unit-cell volume / $Å^3$	1272.74(7)	6590.5(3)	8533.9(4)	1209.33(8)	2135.8(2)
Z	1	4	8	1	2
Calc. density / g cm^{-3}	1.215	1.190	1.477	1.451	1.466
F(000)	496	2544	3920	548	976
Radiation type	MoKα	CuKα	CuKα	CuKα	CuKα
Absorption coefficient / mm^{-1}	0.458	3.221	10.349	4.818	9.701
Crystal size / mm^3	0.22 x 0.22 x 0.06	0.10 x 0.10 x 0.10	0.30 x 0.04 x 0.01	0.20 x 0.15 x 0.05	0.20 x 0.09 x 0.05
2-Theta range / °	7.06-50.69	8.23-133.25	2.29-133.76	7.93-132.95	4.14-133.98
Completeness to max 2-theta	0.985	0.998	0.996	0.990	0.960
No. of reflections measured	10529	3099	161504	16862	22712
No. of independent reflections	4600	3099	15142	4223	7312
Rint	0.0418	?	0.1933	0.0296	0.0894
No. parameters / restraints	289 / 183	261 / 466	894 / 420	261 / 0	448 / 210
Final R1 values (I > 2σ(I))	0.0449	0.1045	0.0875	0.0229	0.0982
Final wR(F^2) values (all data)	0.1127	0.2922	0.1930	0.0612	0.2337
Goodness-of-fit on F^2	0.973	1.035	1.099	1.050	1.168
Largest difference peak & hole / e $Å^{-3}$	0.740, -0.412	1.607, -2.097	1.629, -0.947	0.333, -0.372	2.108, -1.636

	**6**	**7**	**8**	**9**
CCDC number	2185398	2185405	2185406	2185403
Cambridge data number	DW_B2_0308	DW_B2_0341	DW_B2_0331	DW_B1_0421
Chemical formula	$C_{30}H_{64}Cu_2O_{14}Zr_2$	$C_{34}H_{75.33}Mn_{1.67}O_{16}Zr_{2.33}$	$C_{30}H_{64}O_{14}Zn_2Zr_2$	$C_{40}H_{78}Al_2O_{18}Zr_2$
Formula weight	958.33	1044.57	961.99	1083.42
Temperature / K	180(2)	180(2)	180(2)	180(2)
Crystal system	triclinic	triclinic	triclinic	tetragonal
Space group	P -1	P -1	P -1	P 41 21 2
a / Å	9.6359(5)	9.5518(7)	9.0482(7)	13.8442(3)
b / Å	11.5075(6)	11.5838(8)	11.8709(9)	13.8442(3)
c / Å	12.2022(6)	12.3534(8)	12.0327(9)	27.5791(8)
alpha / °	65.744(2)	73.536(3)	106.896(2)	90
beta / °	67.199(2)	81.850(3)	103.685(2)	90
gamma / °	72.073(2)	70.454(3)	109.617(2)	90
Unit-cell volume / $Å^3$	1119.28(10)	1233.61(15)	1081.85(14)	5285.9(3)
Z	1	1	1	4
Calc. density / g cm^{-3}	1.422	1.406	1.477	1.361
F(000)	494	542	496	2272
Radiation type	CuK\a	CuK\a	CuK\a	CuK\a
Absorption coefficient / mm^{-1}	5.230	7.811	5.562	4.098
Crystal size / mm^3	0.16 x 0.16 x 0.12	0.14 x 0.14 x 0.08	0.24 x 0.22 x 0.06	0.20 x 0.08 x 0.06
2-Theta range / °	8.33-134.92	12.05-133.31	8.65-133.16	7.14-133.27
Completeness to max 2-theta	0.983	0.986	0.984	0.999
No. of reflections measured	15999	17990	15485	59980
No. of independent reflections	3971	4307	3763	4691
Rint	0.0498	0.0521	0.0286	0.0544
No. parameters / restraints	237 / 12	275 / 46	224 / 0	289 / 15
Final R1 values (I > 2σ(I))	0.0528	0.0529	0.0196	0.0454
Final wR(F^2) values (all data)	0.1553	0.1594	0.0511	0.1370
Goodness-of-fit on F^2	1.091	1.046	1.062	1.089
Largest difference peak & hole / e $Å^{-3}$	0.657, -0.964	0.740, -0.841	0.317, -0.471	0.896, -0.921
Flack parameter	—	—	—	0.013(5)

Geometrical analysis for 1–8

The central {M_2O_{10}} core of each complex comprises a pair of MO_6 octahedra sharing one edge. The idealised point symmetry of this unit is D_{2h}.

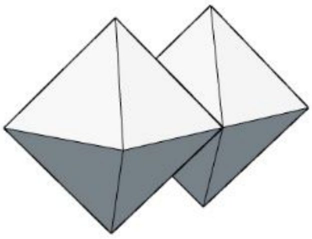

The M′ atoms define a second type of coordination polyhedron with idealised shapes: **1** = MO_4 tetrahedron; **3**,**5**,**8** = MO_5 trigonal bipyramid; **6** = MO_5 square-based pyramid. These attach to the {M_2O_{10}} core through one triangular face. The point symmetry of the resulting {$M_2M'_2O_x$} core is reduced to C_{2h} (retaining the inversion centre and vertical mirror plane).

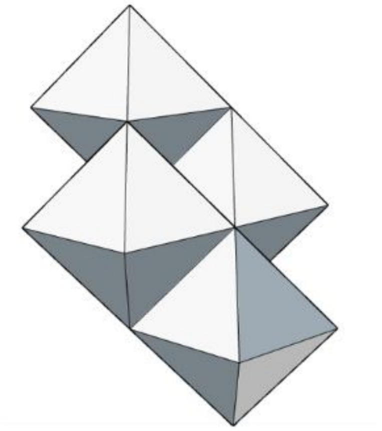

2, 4, 7: {$M_2M'_2O_{16}$} core. Four edge-sharing octahedra.

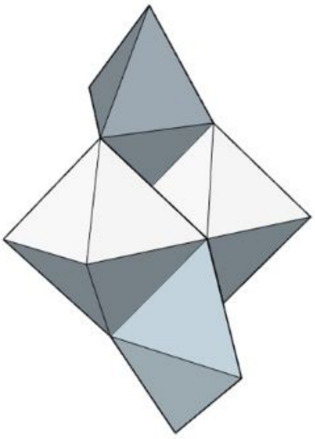

3, 5, 8: {$M_2M'_2O_{14}$} core. 2 x Trigonal bipyramid attached to the central core.

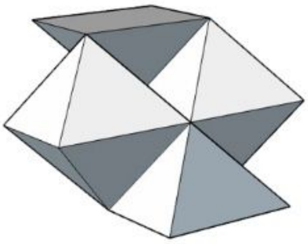

6: {$M_2M'_2O_{14}$} core. 2 x Square-based pyramid attached to the central core.

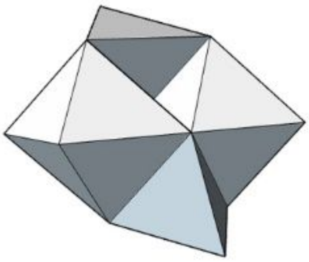

1: {$M_2M'_2O_{12}$} core. 2 x Tetrahedron attached to the central core.

Compounds **2**, **4** and **7**, comprising all octahedral M/M′ coordination sites, represent a section from the 2-D CdI_2 structure type.

Table S2: Quantitative measures of the coordination environments.

(1) Distortion from octahedral geometry for the 6-coordinate M/M′ sites is quantified as described in: Du, Tu, Zhang, Han, Liu, Zauscher, Mitzi, Inorg. Chem. (2017). **56**, 9291–9302.

Mean octahedral quadratic elongation:

$$\langle \lambda_{oct} \rangle = \frac{1}{6}\sum_{i=1}^{6}\left(\frac{d_i}{d_0}\right)^2$$

Bond angle variance:

$$\sigma^2 = \frac{1}{11}\sum_{i=1}^{12}(\theta_i - 90°)^2$$

Where d_i are the M–O bond lengths, d_0 is the M–O bond length for a regular octahedron with equivalent volume and θ_i are the *cis* O–M–O bond angles. $<\lambda_{oct}> = 1$ (dimensionless) and $\sigma^2 = 0$ (degrees^2) describes a perfect octahedron. $<\lambda_{oct}> > 1$ reflect the degree of bond length distortion and $\sigma^2 > 0$ reflect the degree of bond angle distortion.

(2) The continuous shape measure (CShM) quantifies the degree of distortion relative to an idealised scaled shape (either an octahedron, tetrahedron or trigonal bipyramid (with vertices equidistant from the centre)). CShM calculations were carried out using: https://csm.ouproj.org.il/molecule (Tuvi-Arad, G. Alon and D. Avnir).

(3) For 5-coordinate coordination environments: the Addison parameter, τ_5, was also calculated at: http://kchn.pg.gda.pl/geom (Andrzej Okuniewski). $\tau_5 = 1$ describes a regular trigonal bipyramid and $\tau_5 = 0$ describes a regular square-based pyramid.

(4) The polyhedral volume was calculated using: https://www.codeproject.com/Articles/13666/Polyhedra-Volume-Calculations-A-JavaScript-Implementation (@dszarkow)

	M	Coord. No.	Shape	Polyhedral Vol (Å^3)	$<\lambda_{oct}>$	σ^2 (deg^2)	CShM (O_h)
**1**	Zr	6	Octahedral	11.70	1.029	82.8	1.09
**2**	Mg	6	Octahedral	11.83	1.022	71.7	0.66
**3 (mol 1)**	Zr	6	Octahedral	11.75	1.033	93.0	1.41
**3 (mol 2)**	Zr	6	Octahedral	11.76	1.033	92.5	1.40
**4**	Ni	6	Octahedral	11.73	1.013	38.5	0.58
**5 (mol 1)**	Zr	6	Octahedral	11.77	1.032	88.2	1.25
**5 (mol 2)**	Zr	6	Octahedral	11.84	1.031	87.3	1.20
**6**	Zr	6	Octahedral	11.76	1.025	76.6	0.67
**7**	Zr/Mn	6	Octahedral	12.12	1.026	76.4	0.88
**8**	Zr	6	Octahedral	11.80	1.025	71.9	0.98

	M′	Coord No.	Shape	Polyhedral Vol (Å^3)	$<\lambda_{oct}>$	σ^2 (deg^2)	τ_5	CShM
**1**	Li	4	Tetrahedron	3.32	—	—	—	3.47
**2**	Zr	6	Octahedron	11.59	1.025	81.8	—	0.87
**3 (mol 1)**	Co	5	Trig. Bipyr.	7.08	—	—	0.84	1.12
**3 (mol 2)**	Co	5	Trig. Bipyr.	7.06	—	—	0.85	1.15
**4**	Zr	6	Octahedron	11.85	1.016	46.9	—	0.89
**5 (mol 1)**	Fe	5	Trig. Bipyr.	7.28	—	—	0.78	37.9

5 (mol 2)	Fe	5	Trig. Bipyr.	7.29	—	—	0.70	56.2
**6**	Cu	5	Square pyr.	5.71	—	—	0.07	8.16
**7**	Zr/Mn	6	Octahedron	13.23	1.032	98.4	—	1.48
**8**	Zn	5**	Trig. Bipyr.	7.29	—	—	0.92	2.89

** Including the long Zn1-O4 contact in the coordination environment.

Table S3: Bond valence sums. R_0 and B parameters for M^{n+}–O^{2-} taken from: Gagne & Hawthorne (2015) *Acta Cryst.* **B71**, 561-578.

	M	Coord. No.	Assumed valence	R_0	B	Bond-valence sum
**1**	Zr	6	4+	1.913	0.406	4.08
**2**	Mg	6	2+	1.608	0.443	2.04
**3 (mol 1)**	Zr	6	4+	1.913	0.406	4.02
**3 (mol 2)**	Zr	6	4+	1.913	0.406	4.04
**4**	Ni	6	2+	1.689	0.347	2.01
**5 (mol 1)**	Zr	6	4+	1.913	0.406	4.06
**5 (mol 2)**	Zr	6	4+	1.913	0.406	3.97
**6**	Zr	6	4+	1.913	0.406	4.05
**7****	Zr	6	4+	1.913	0.406	3.97
**7****	Mn	6	2+	1.740	0.417	2.65
**8**	Zr	6	4+	1.913	0.406	4.02

	M’	Coord No.	Assumed valence	R_0	B	Bond-valence sum
**1**	Li	4	+1	1.062	0.642	1.02
**2**	Zr	6	+4	1.913	0.406	4.08
**3 (mol 1)**	Co	5	+2	1.698	0.376)	2.10
**3 (mol 2)**	Co	5	+2	1.698	0.376	2.11
**4**	Zr	6	+4	1.913	0.406	4.03
**5 (mol 1)**	Fe	5	+2	1.658	0.447	2.06
**5 (mol 2)**	Fe	5	+2	1.658	0.447	2.02
**6**	Cu	5	+2	1.687	0.355	2.06
**7****	Mn	6	+2	1.740	0.417	2.17
**7****	Zr	6	+4	1.913	0.406	3.23
**8**	Zn	5	+2	1.684	0.383	2.04

** For **7**, both sites show separate calculations assuming Zr^{4+} only or Mn^{2+} only. Site 1 (M) is consistent with Zr^{4+}, while site 2 (M’) is closer to Mn^{2+}. This is consistent with the refined site occupancies: Zr(1):Mn(1) = 0.908(2):0.092(2), Zr(2):Mn(2) = 0.258(2):0.742(2).

3. **NMR Spectroscopy**

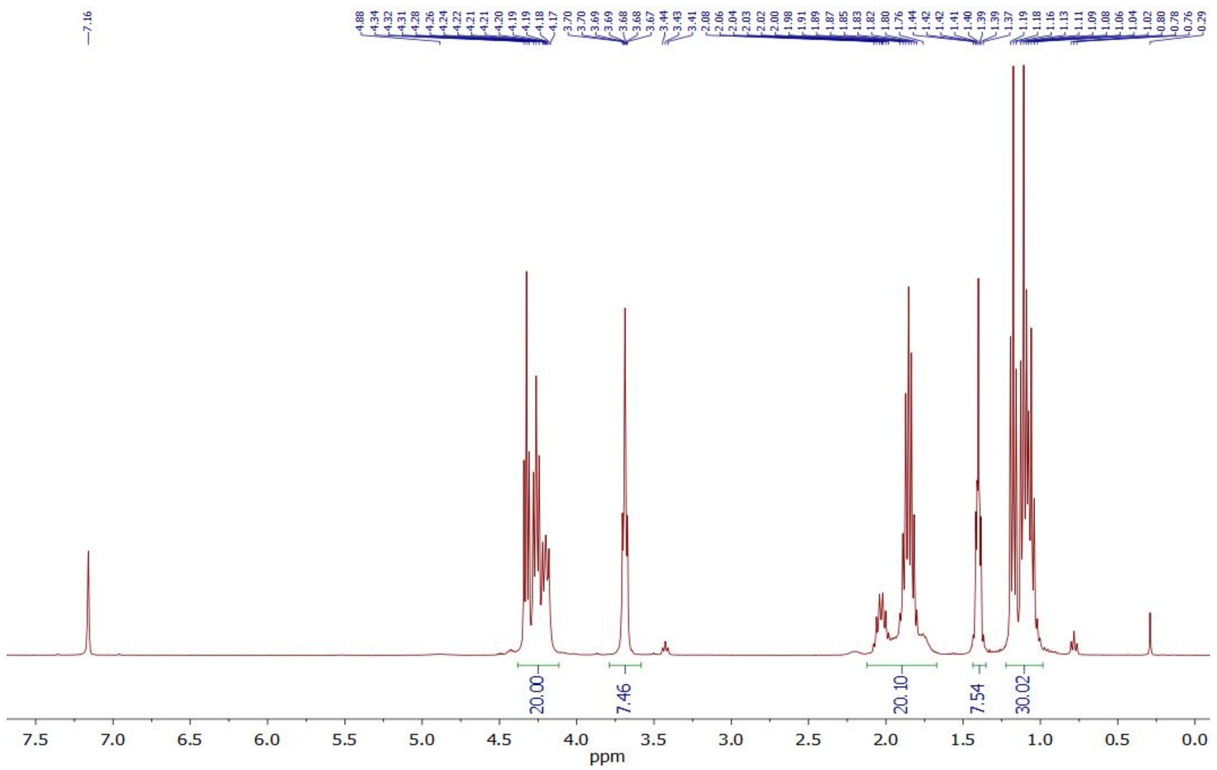

Figure S1: The ^{1}H NMR spectrum of **1** in C_6D_6.

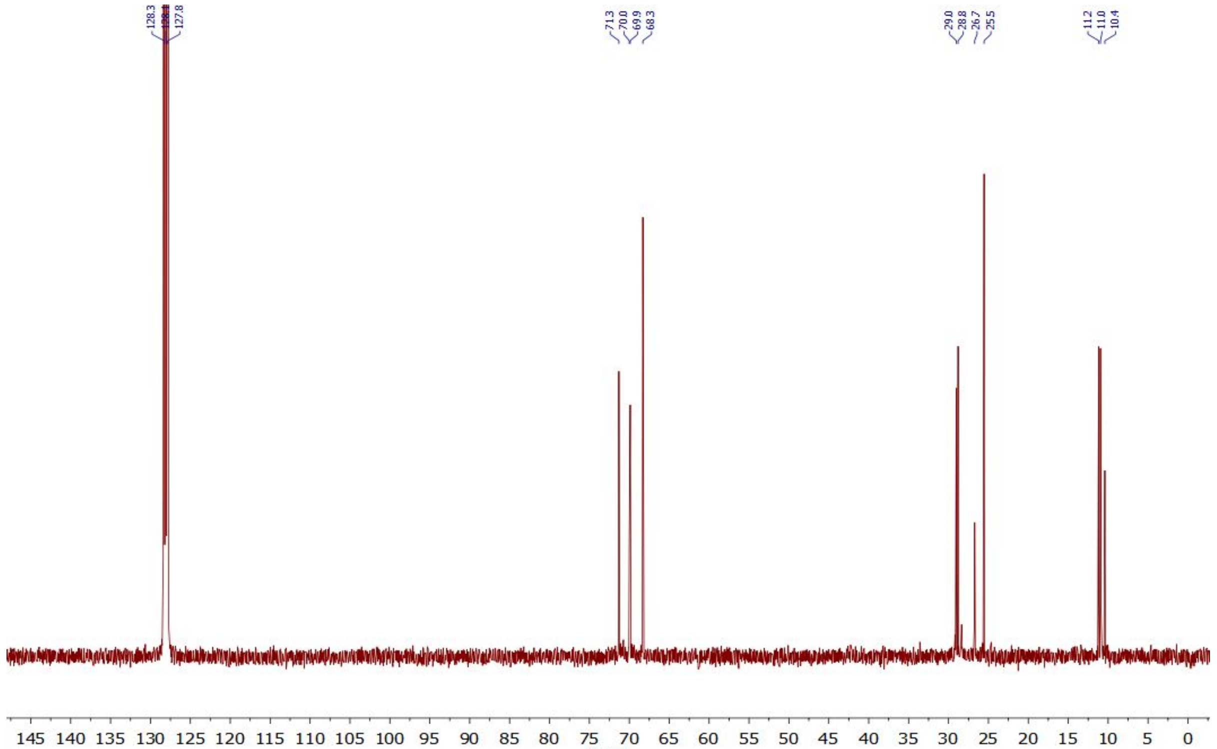

Figure S2: The ^{13}C NMR spectrum of **1** in C_6D_6.

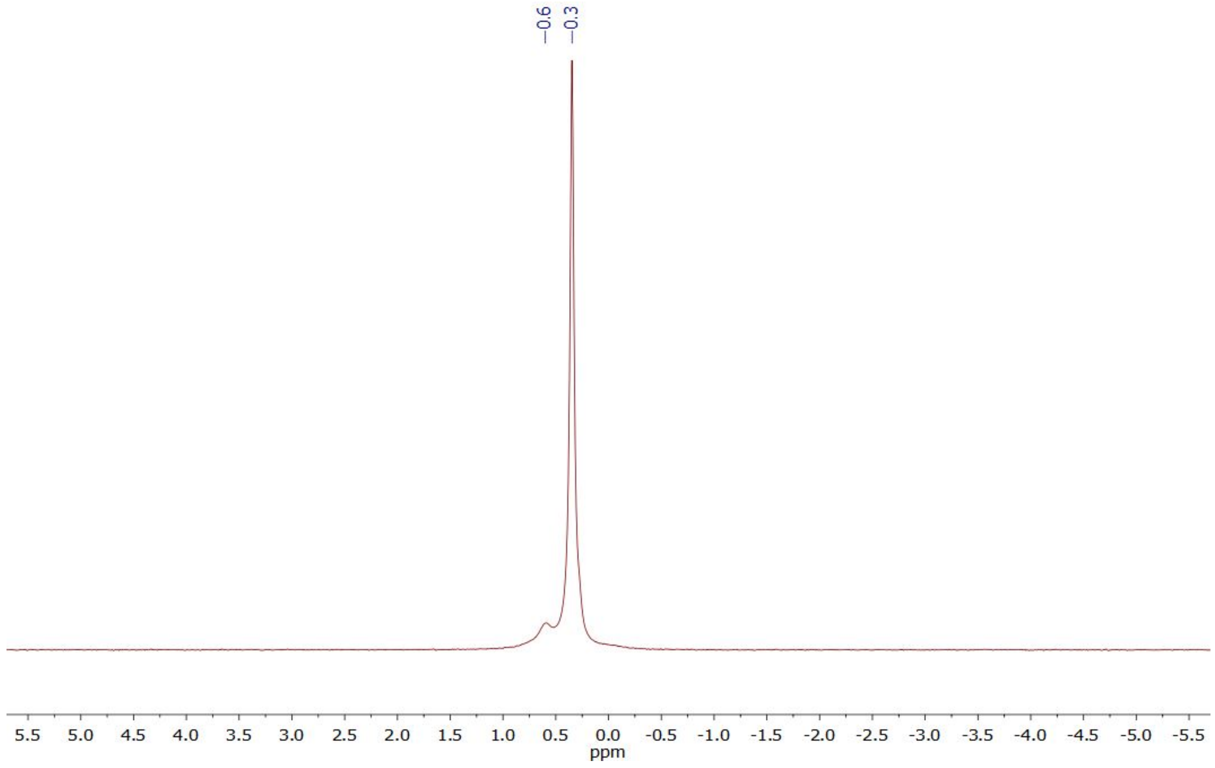

Figure S3: The ^{7}Li NMR spectrum of **1** in C_6D_6.

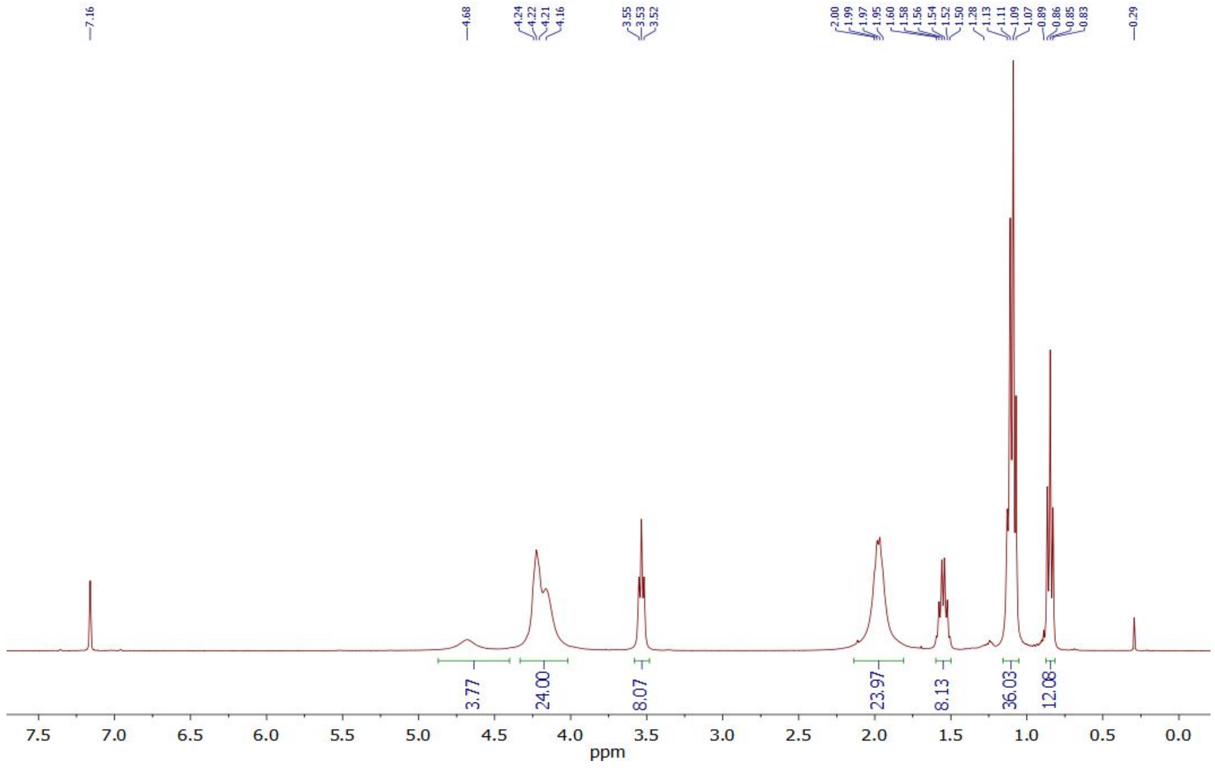

Figure S4: The ^{1}H NMR spectrum of **2** in C_6D_6.

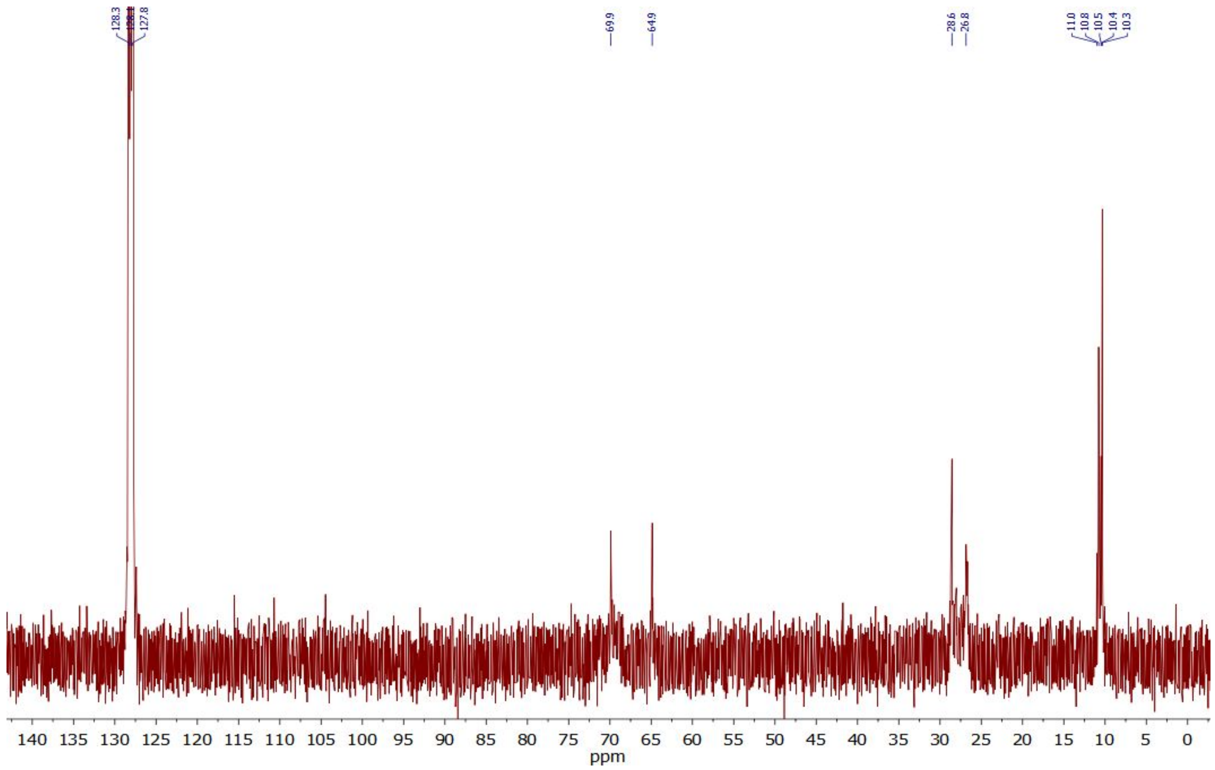

Figure S5: The ^{13}C NMR spectrum of **2** in C_6D_6.

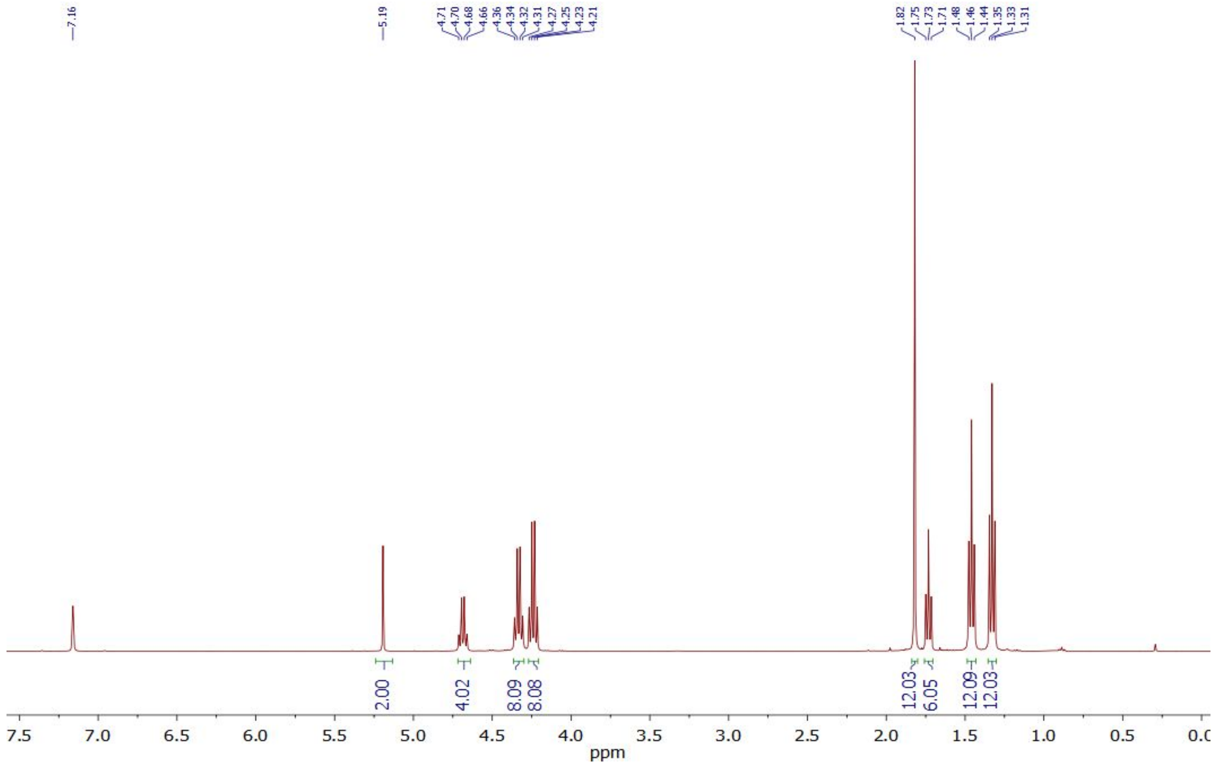

Figure S6: The ^{1}H NMR spectrum of **8** in C_6D_6.

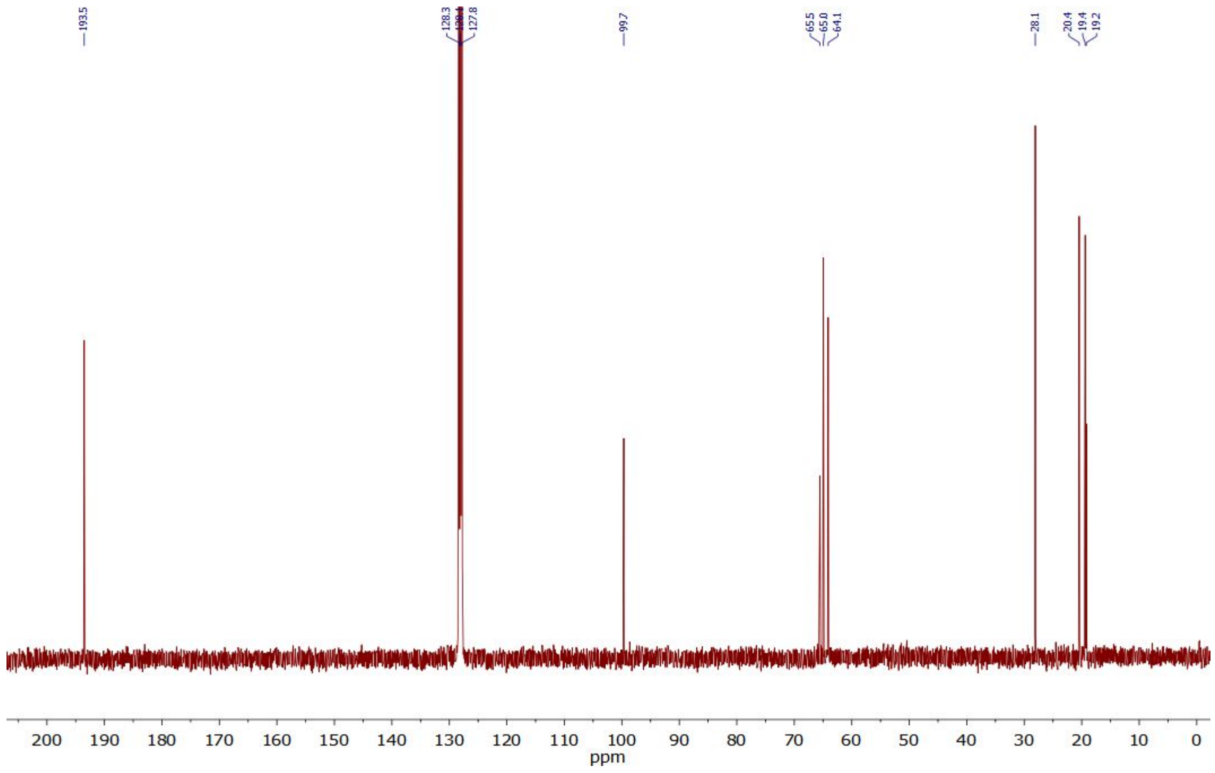

Figure S7: The ^{13}C NMR spectrum of **8** in C_6D_6.

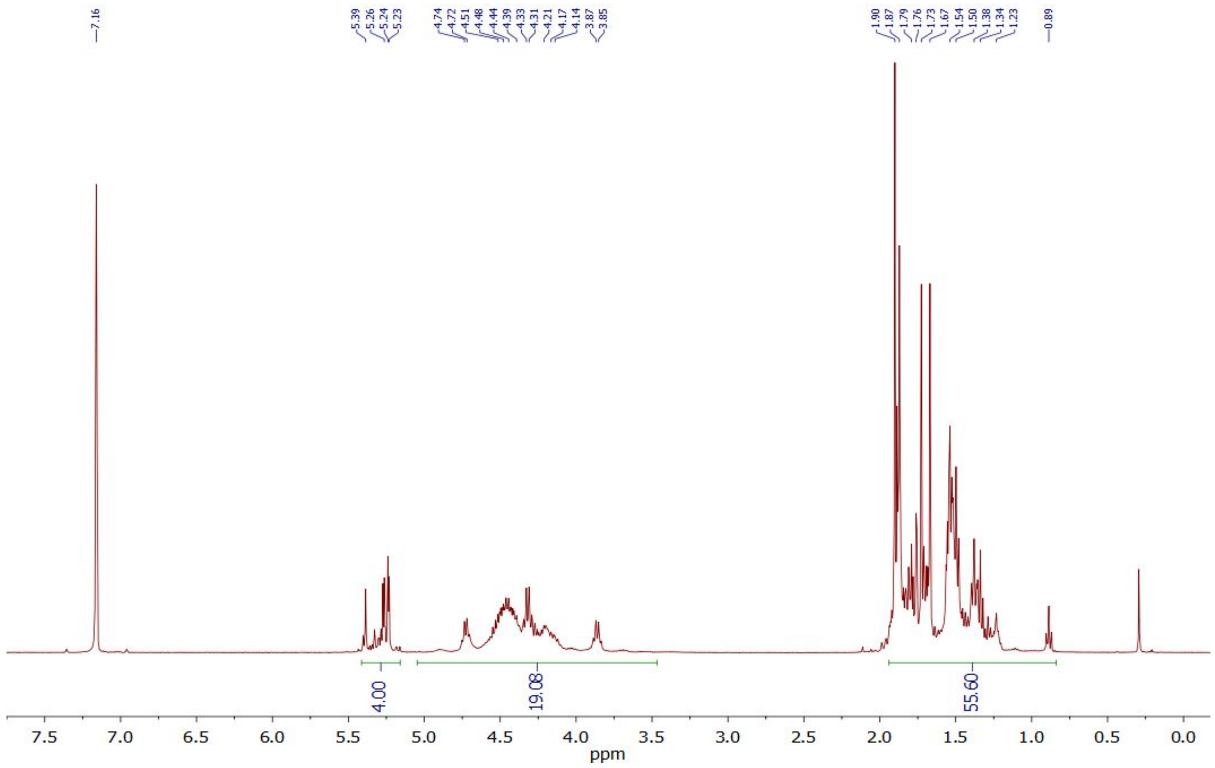

Figure S8: The ^{1}H NMR spectrum of **9** in C_6D_6.

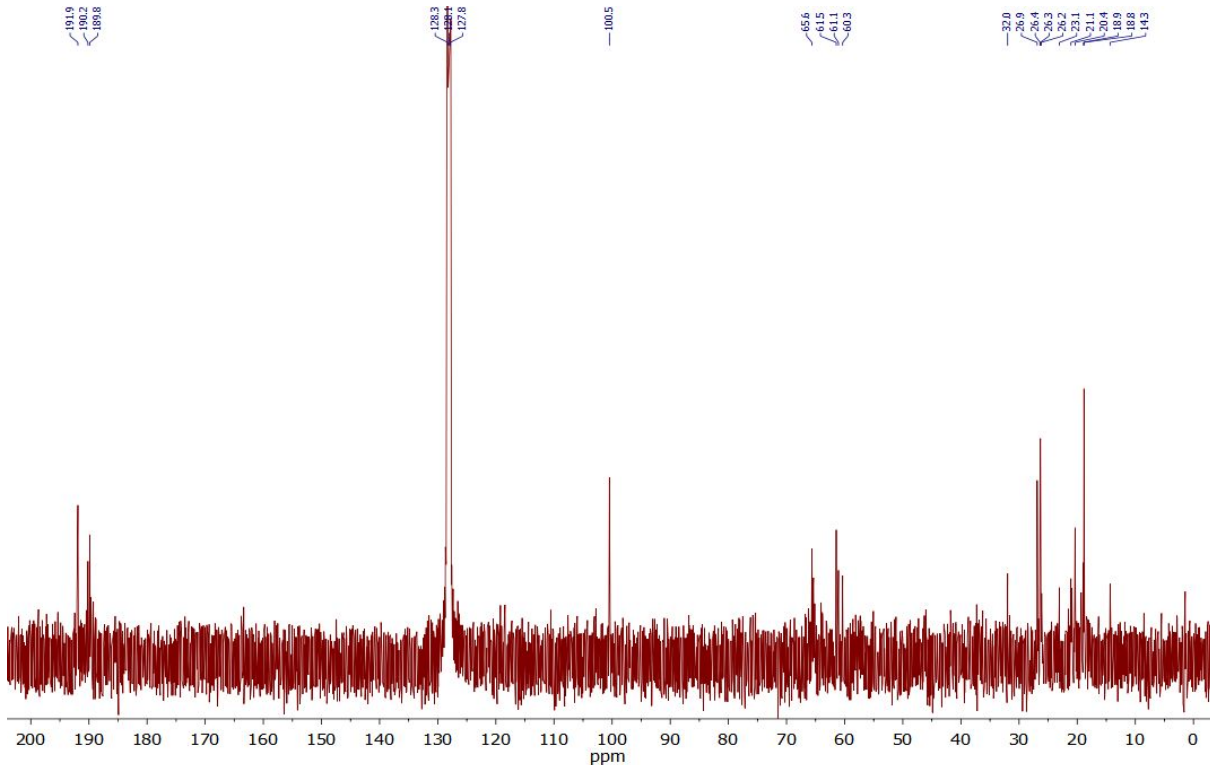

Figure S9: The ^{13}C NMR spectrum of **9** in C_6D_6.

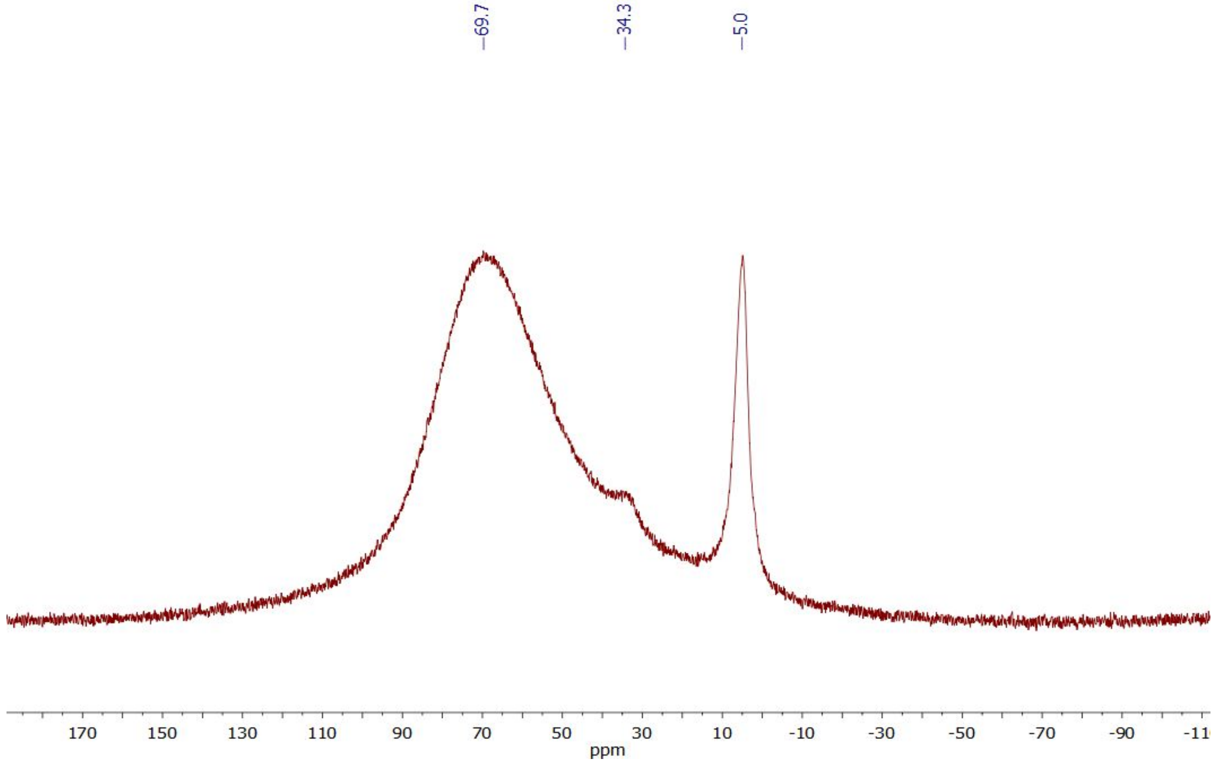

Figure S10: The ^{27}Al NMR spectrum of **9** in C_6D_6.

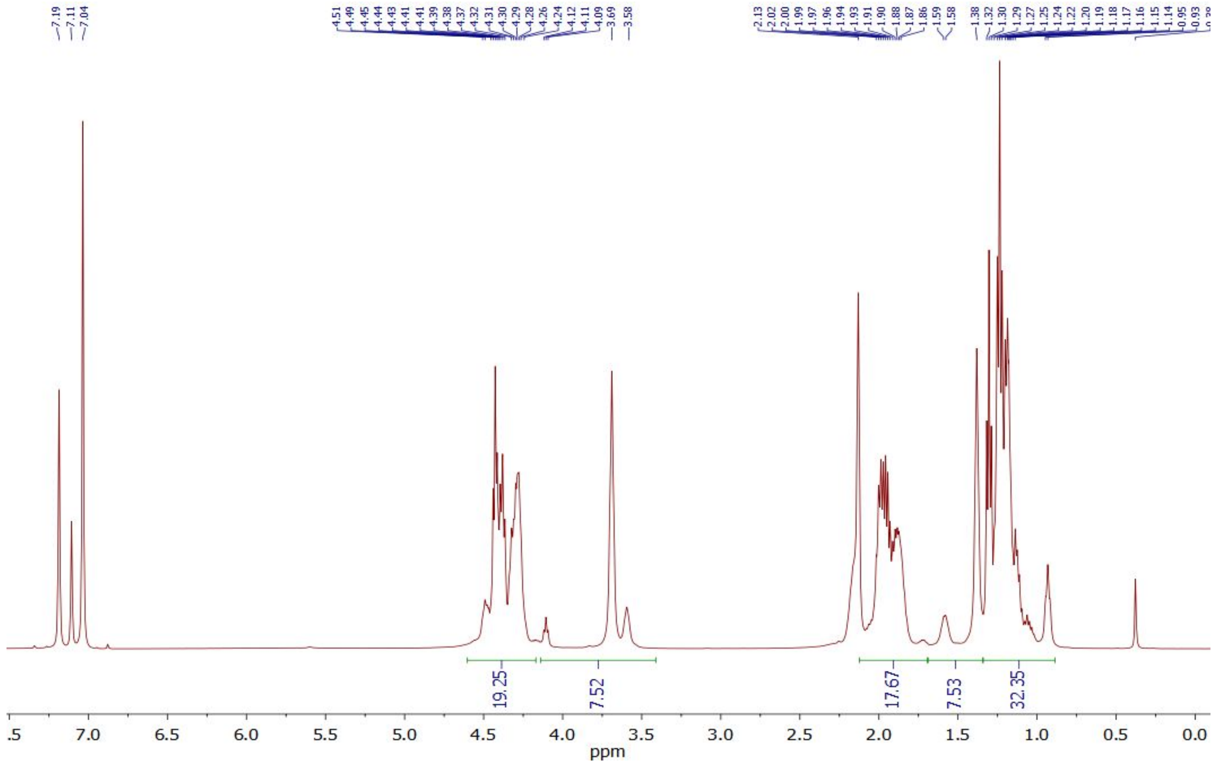

Figure S11: The ^{1}H NMR spectrum of **1** in $C_6D_5CD_3$ at –50 °C.

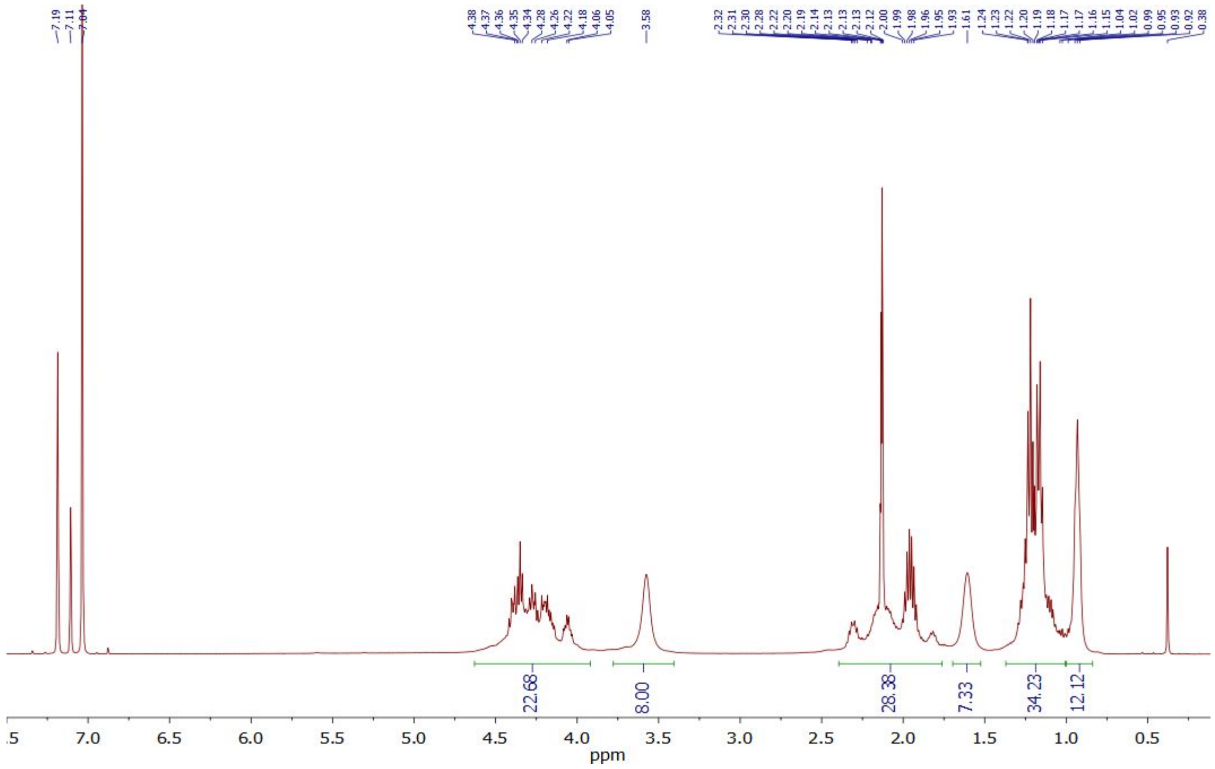

Figure S12: The ^{1}H NMR spectrum of **2** in $C_6D_5CD_3$ at –50 °C.

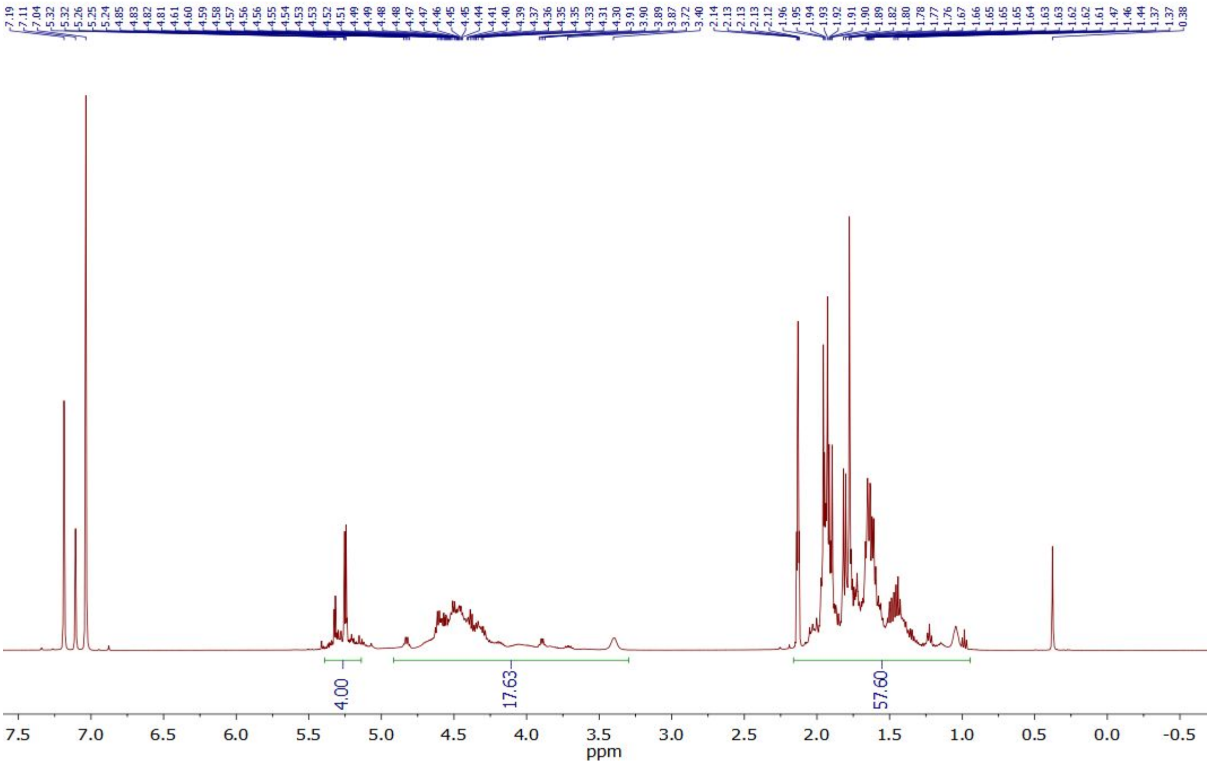

Figure S13: The ^{1}H NMR spectrum of **9** in $C_6D_5CD_3$ at –50 °C.

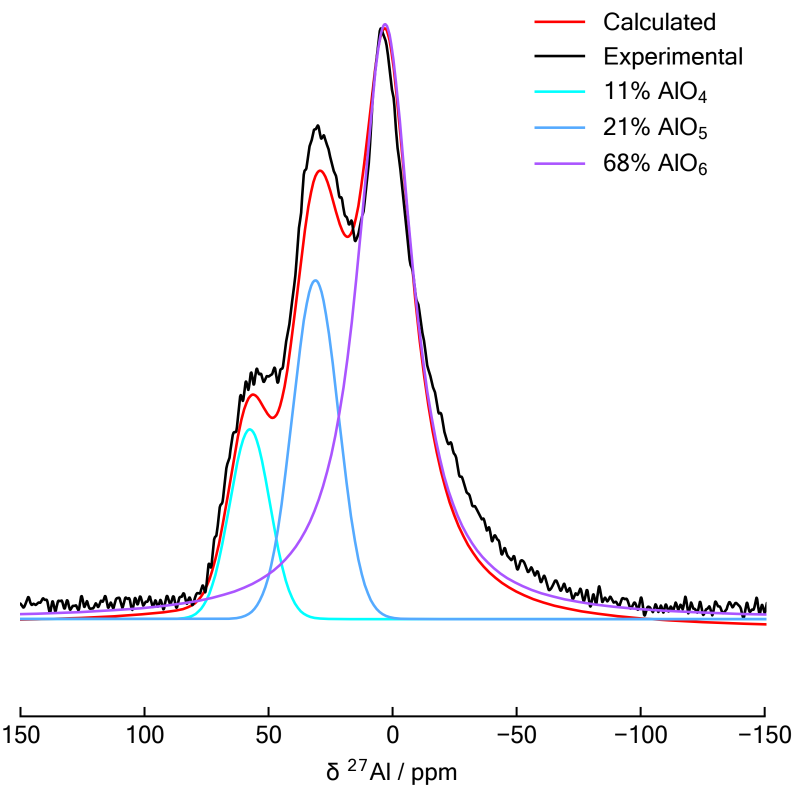

Figure S14: The solid-state ^{27}Al NMR spectrum of the products from heating complex **9** at 800 °C for 4 hours.

4. IR Spectroscopy

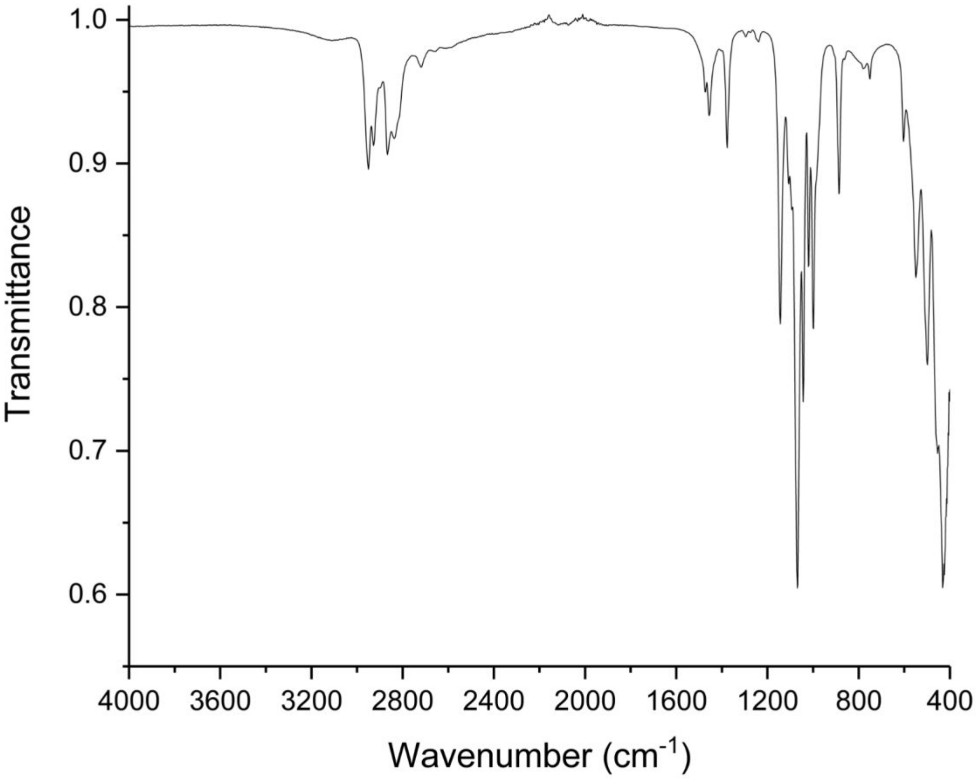

Figure S15: The IR spectrum of **2**.

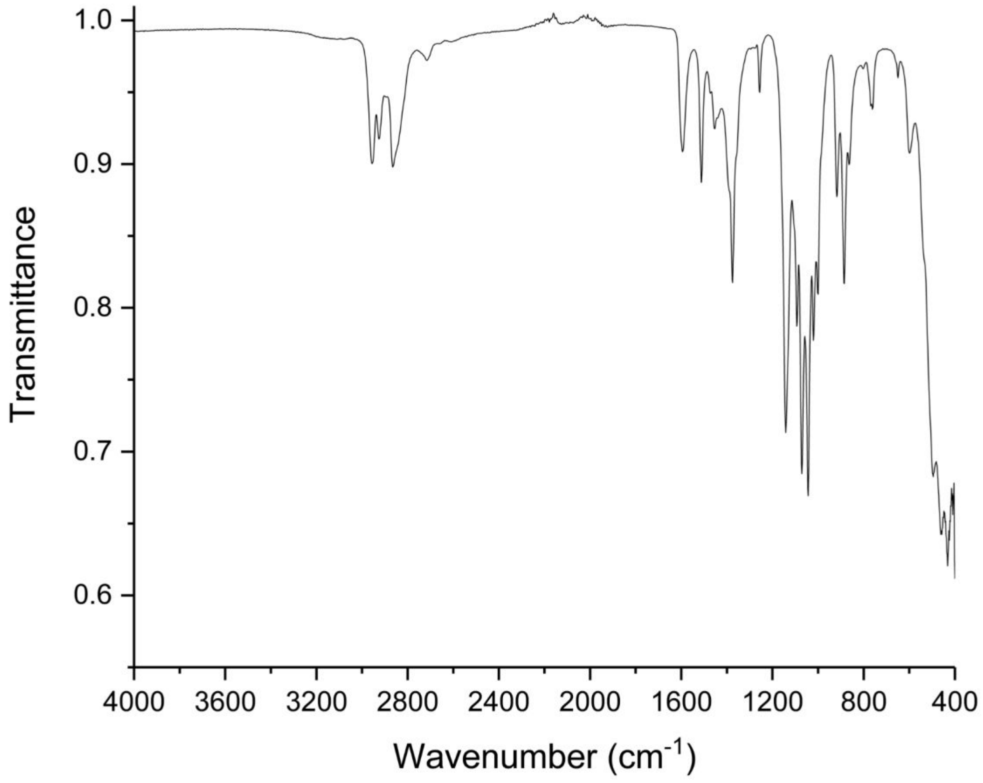

Figure S16: The IR spectrum of **7**.

5. **UV-visible Spectroscopy**

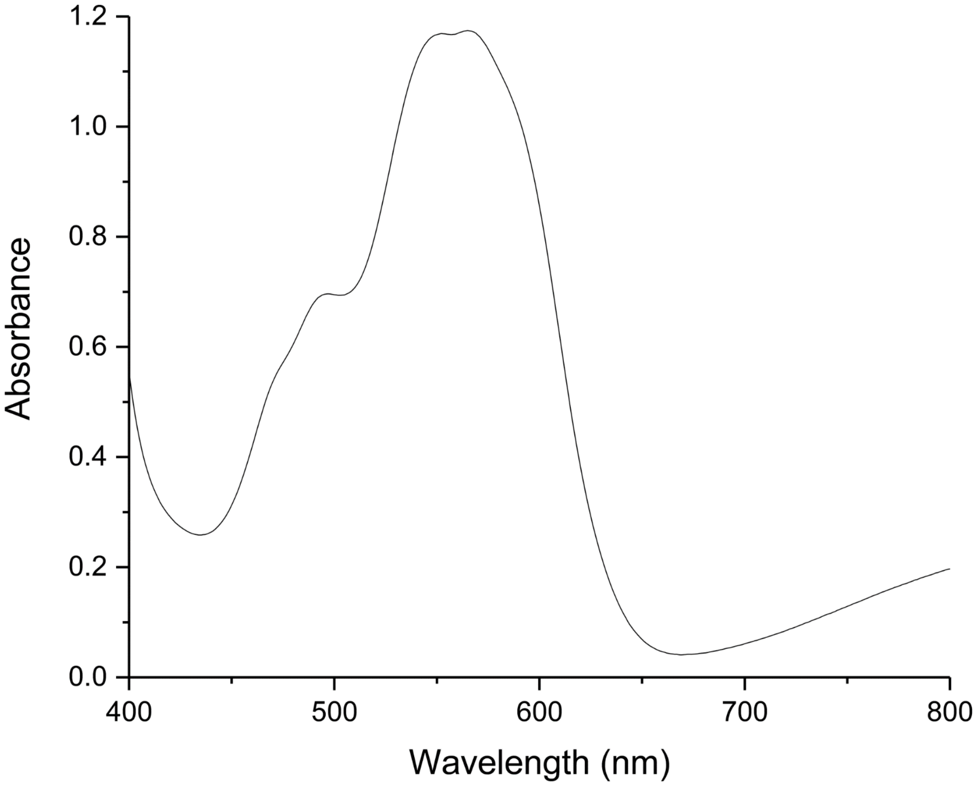

Figure S17: The UV-visible spectrum of **3** in n-hexane.

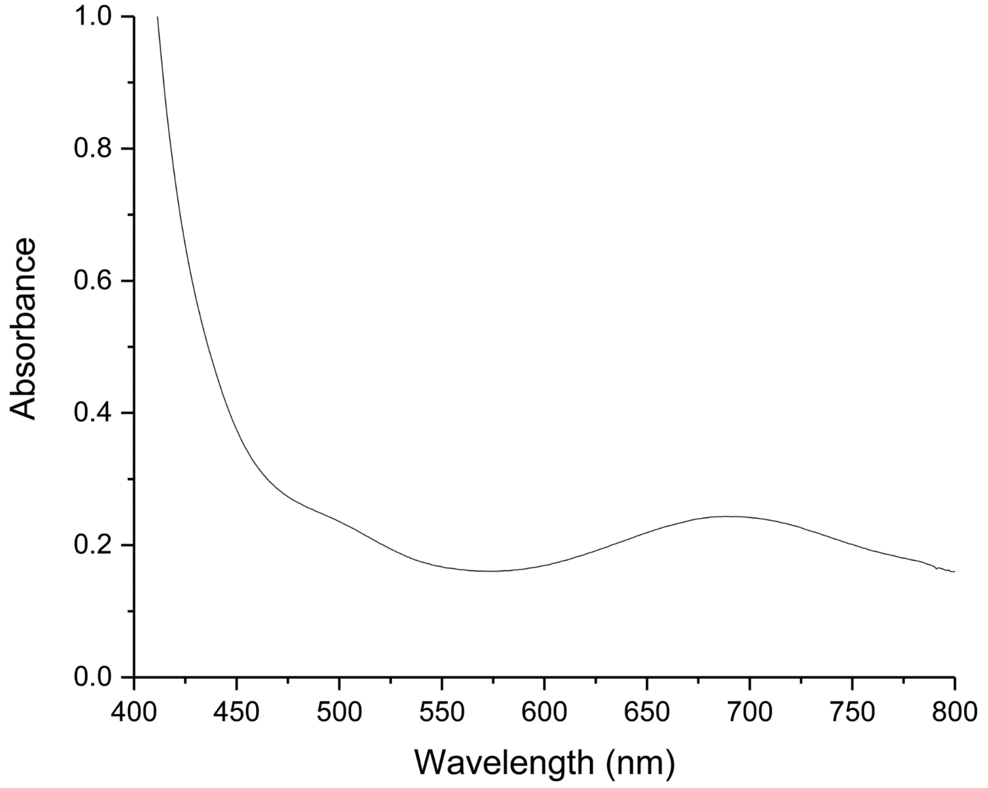

Figure S18: The UV-visible spectrum of **4** in n-hexane.

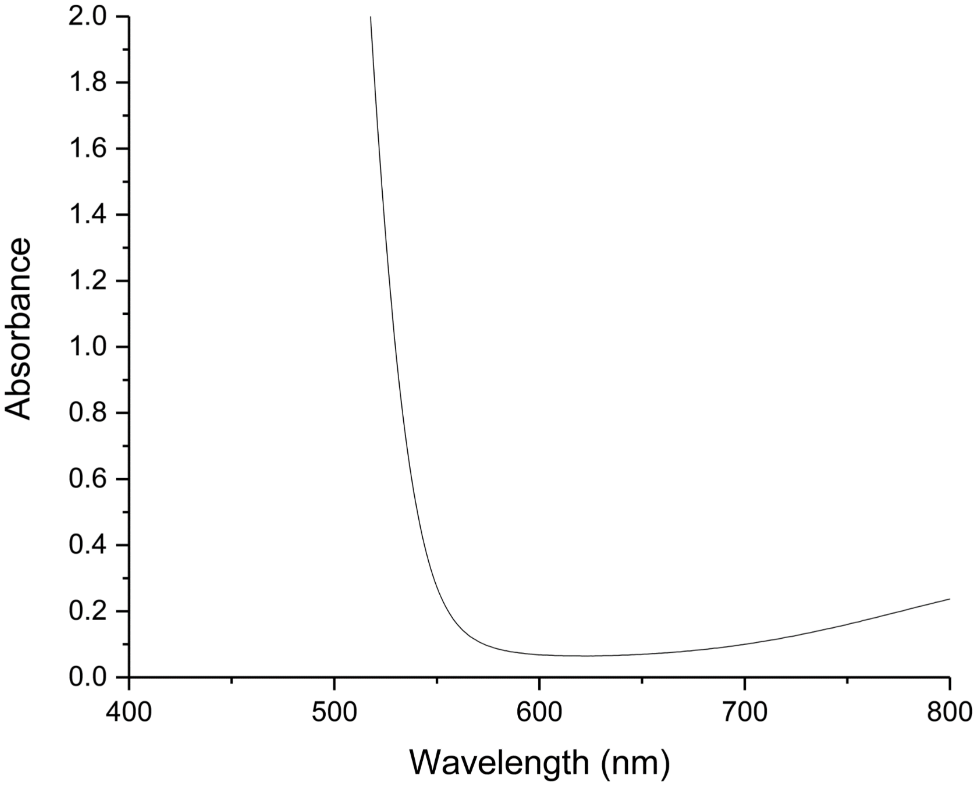

Figure S19: The UV-visible spectrum of **5** in n-hexane.

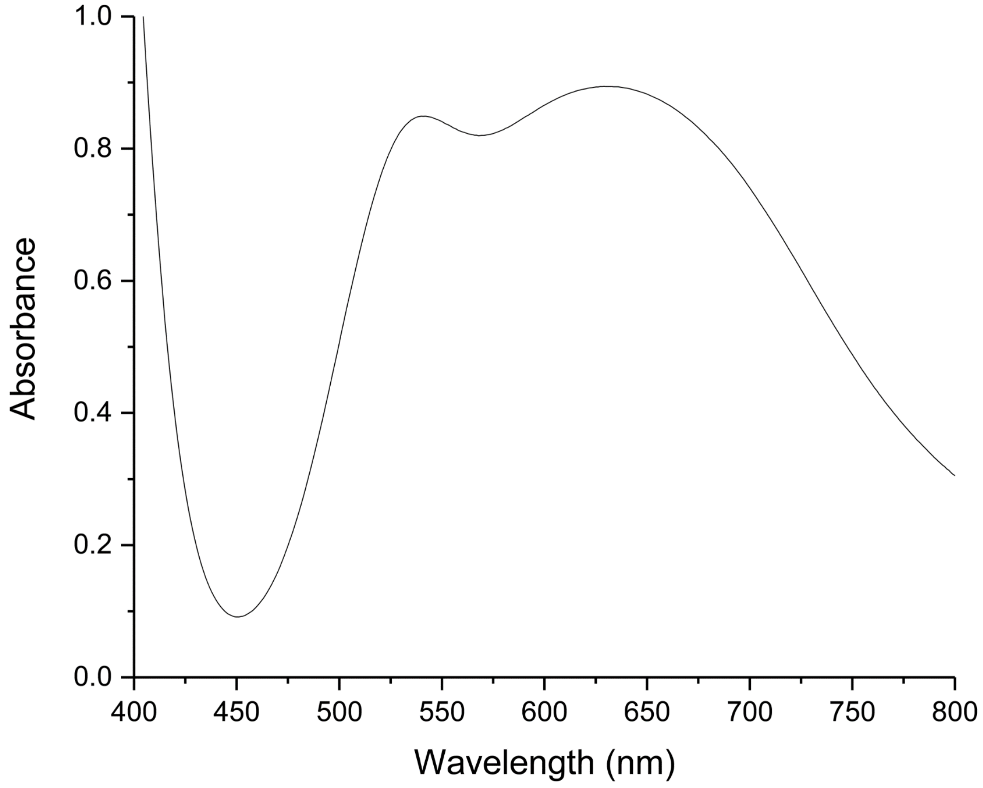

Figure S20: The UV-visible spectrum of **6** in n-hexane.

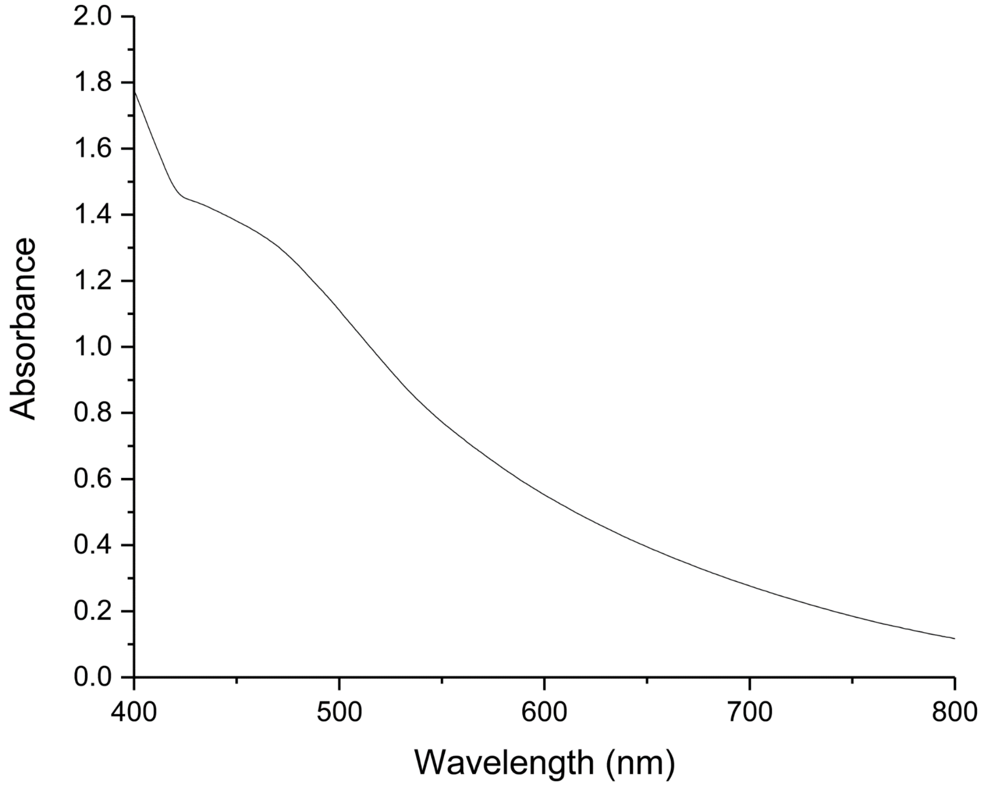

Figure S21: The UV-visible spectrum of **7** in n-hexane.

6. PXRD

Table S4: Phases and lattice parameters identified for the decomposition products of each of the complexes after heating at 800 °C (1000 °C for **9**), as obtained from Rietveld refinements.

**Complex**	**Empirical Formula**	R_{wp} **/ %**	**λ / Å**	**Refined phases**	**Space group**	**Wt%**	***a*** **/ Å**	***b*** **/ Å**	***c*** **/ Å**	**β / °**
**1**	$Li_{0.84}ZrO_{2.42}$	2.07	0.82697	ZrO_2 (M)<br>Li_2ZrO_3	$P2_1/c$<br>Cc	52.7(3)<br>47.3(3)	5.14651(4)<br>5.4313(19)	5.20986(5)<br>9.0287(6)	5.31487(5)<br>5.4323(19)	99.2068(7)<br>112.697(3)
**2**	$Mg_{0.96}ZrO_{3.54}$	3.84	0.82697	$Zr_{0.36}Mg_{0.64}O_{2-\delta}$ (C)<br>ZrO_2 (T)<br>MgO	$Fm{-}3m$<br>$P4_2/nmc$<br>$Fm{-}3m$	26.1(13)<br>54.7(14)<br>19.2(6)	5.08732(6)<br>3.59639(12)<br>4.21481(11)	-<br>-<br>-	-<br>5.1311(3)<br>-	-<br>-<br>-
**3**	$Co_{1.22}ZrO_{3.55}$	2.50	0.82697	$Zr_{0.75}Co_{0.25}O_{2-\delta}$ (T)<br>Co_3O_4<br>ZrO_2 (M)	$P4_2/nmc$<br>$F{-}43m$<br>$P2_1/c$	45.0(3)<br>36.75(19)<br>18.28(13)	3.59551(2)<br>8.08441(3)<br>5.1487(3)	-<br>-<br>5.2014(3)	5.18325(6)<br>-<br>5.3183(3)	-<br>-<br>99.025(4)
**4**	$Ni_{1.5}ZrO_{3.7}$	1.90	0.82601	NiO<br>$Zr_{0.76}Ni_{0.24}O_{2-\delta}$ (T)<br>ZrO_2 (M)	$Fm{-}3m$<br>$P4_2/nmc$<br>$P2_1/c$	43.9(2)<br>45.9(3)<br>10.15(19)	4.18026(5)<br>3.59440(4)<br>5.1562(11)	-<br>-<br>5.1936(12)	-<br>5.18197(7)<br>5.3131(11)	-<br>-<br>98.883(15)
**5**	$Fe_{0.51}ZrO_{2.77}$	2.03	0.82718	Fe_2O_3<br>$Zr_{0.85}Fe_{0.15}O_{2-\delta}$ (T)<br>ZrO_2 (M)	$R{-}3c$<br>$P4_2/nmc$<br>$P2_1/c$	17.9(11)<br>69.5(18)<br>12.6(8)	5.04012(17)<br>3.6015(11)<br>5.1192(16)	-<br>-<br>5.253(2)	13.7662(7)<br>5.086(3)<br>5.2846(19)	-<br>-<br>98.679(16)
**6**	$Cu_{1.07}ZrO_{3.07}$	4.43	0.82697	CuO<br>ZrO_2 (T)<br>ZrO_2 (M)	Cc<br>$P4_2/nmc$<br>$P2_1/c$	41.0(5)<br>1.2(11)<br>57.8(7)	4.68983(8)<br>3.5954(14)<br>5.14784(6)	3.42223(6)<br>-<br>5.20253(6)	5.13437(10)<br>5.192(4)<br>5.32284(4)	99.2910(15)<br>-<br>99.1428(9)
**7**	$Mn_xZr_yO_z$	3.59	0.82601	$Zr_{0.9}Mn_{0.1}O_{2-\delta}$ (T)<br>Pawley phase<br>Mn_3O_4	$P4_2/nmc$<br>$Fm{-}3m$<br>$I4_1/amd$	n/a	3.59007(12)<br>4.96152(19)<br>5.7632(3)	-<br>-<br>-	5.1762(3)<br>-<br>9.4619(9)	-<br>-<br>-
**8**	$Zn_{1.03}ZrO_{2.99}$	2.53	0.82718	ZnO<br>ZrO_2 (M)	$P6_3mc$<br>$P2_1/c$	39.80(10)<br>60.20(10)	3.25093(3)<br>5.14876(6)	3.25093(3)<br>5.20553(6)	5.20404(8)<br>5.32042(6)	-<br>99.1647(8)
**9** (1000 °C)	$Al_{0.33}ZrO_{2.50}$	2.997	1.54060	ZrO_2 (T)<br>Al_2O_3	$P4_2/nmc$<br>$Fd{-}3m$	87.9(16)<br>12.1(16)	3.5939(11)<br>7.912(3)	-<br>-	5.1909(16)<br>-	-<br>-

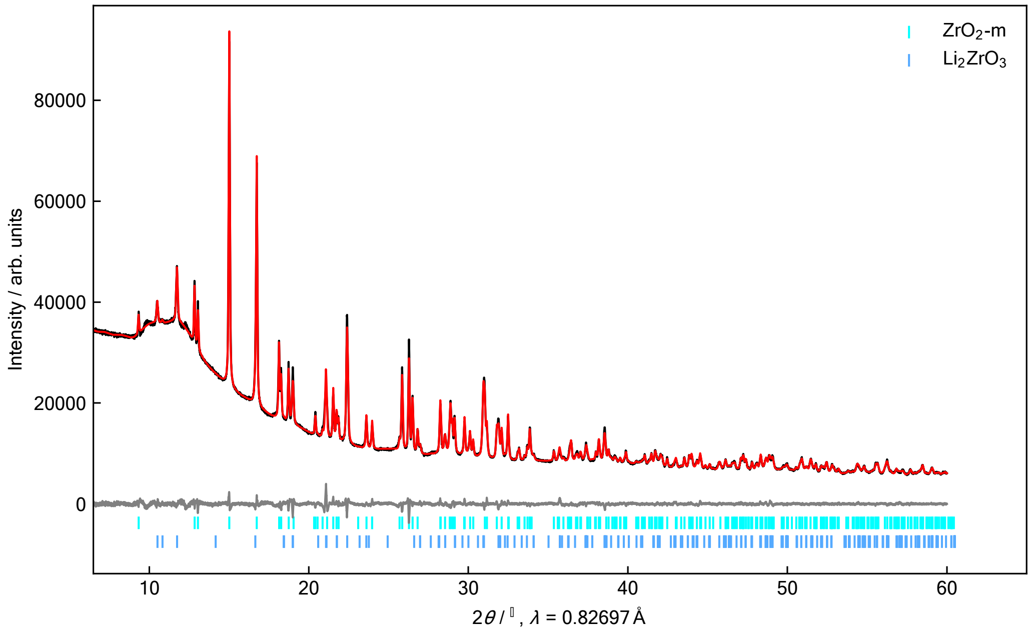

Figure S22: The PXRD and Rietveld refinement for the decomposition products from heating complex **1** at 800 °C for 4 hours in air.

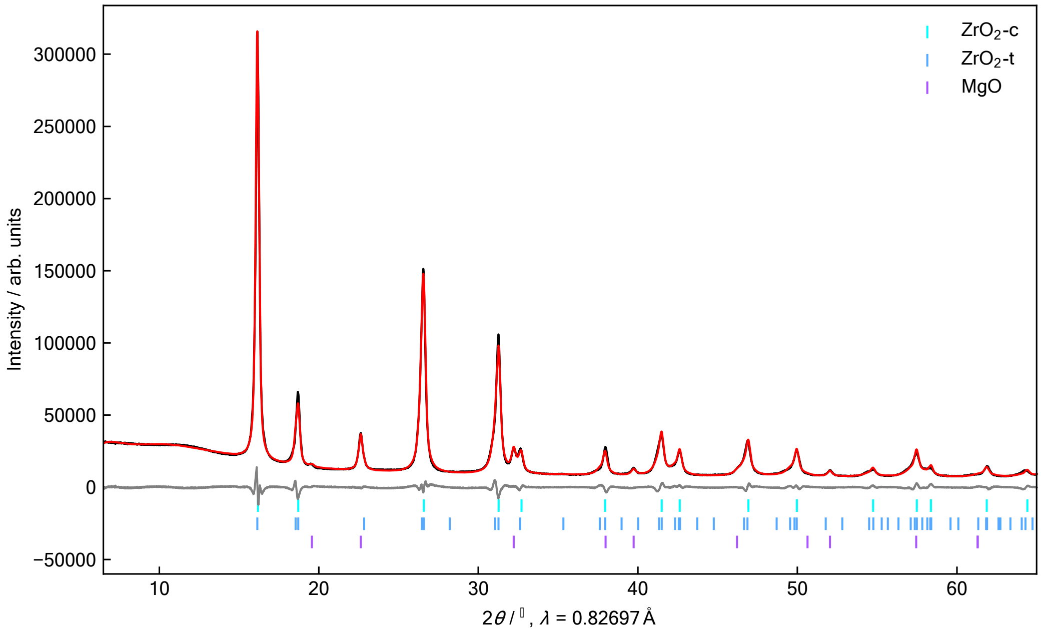

Figure S23: The PXRD and Rietveld refinement for the decomposition products from heating complex **2** at 800 °C for 4 hours in air.

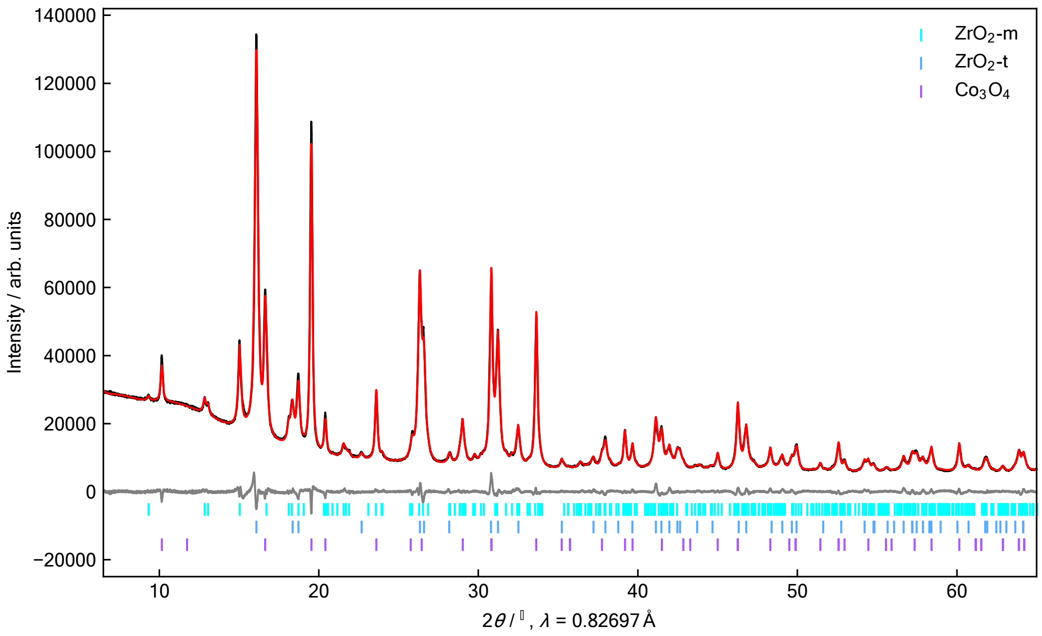

Figure S24: The PXRD and Rietveld refinement for the decomposition products from heating complex **3** at 800 °C for 4 hours in air.

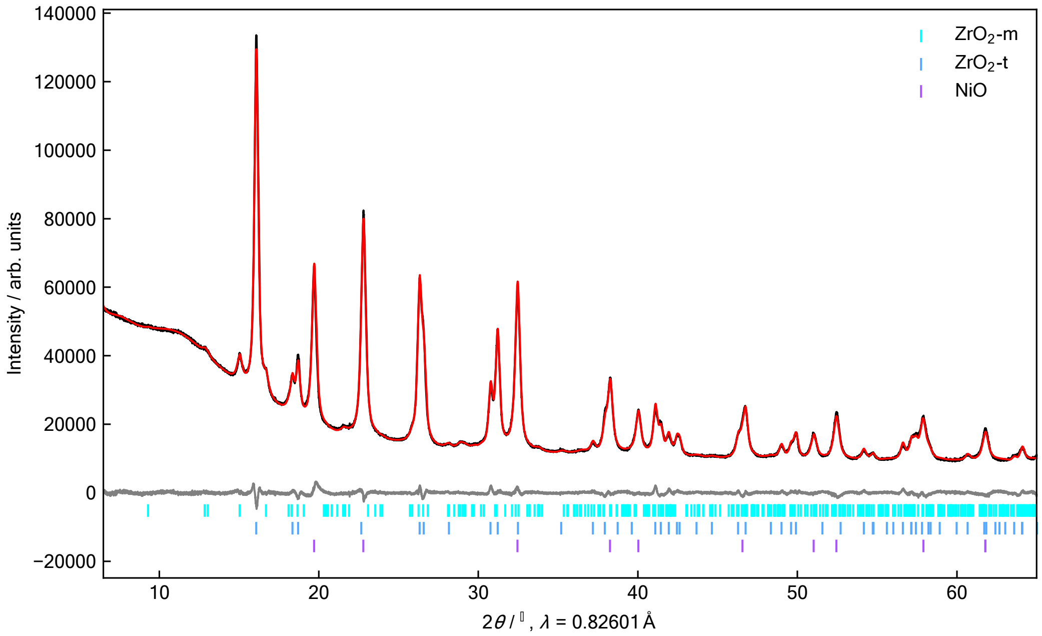

Figure S25: The PXRD and Rietveld refinement for the decomposition products from heating complex **4** at 800 °C for 4 hours in air.

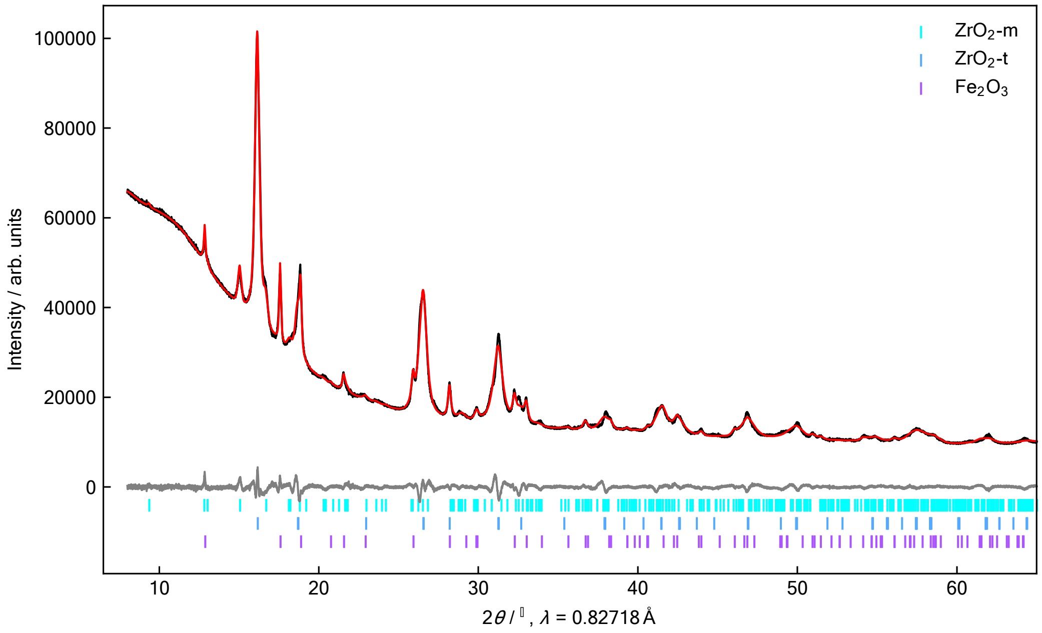

Figure S26: The PXRD and Rietveld refinement for the decomposition products from heating complex **5** at 800 °C for 4 hours in air.

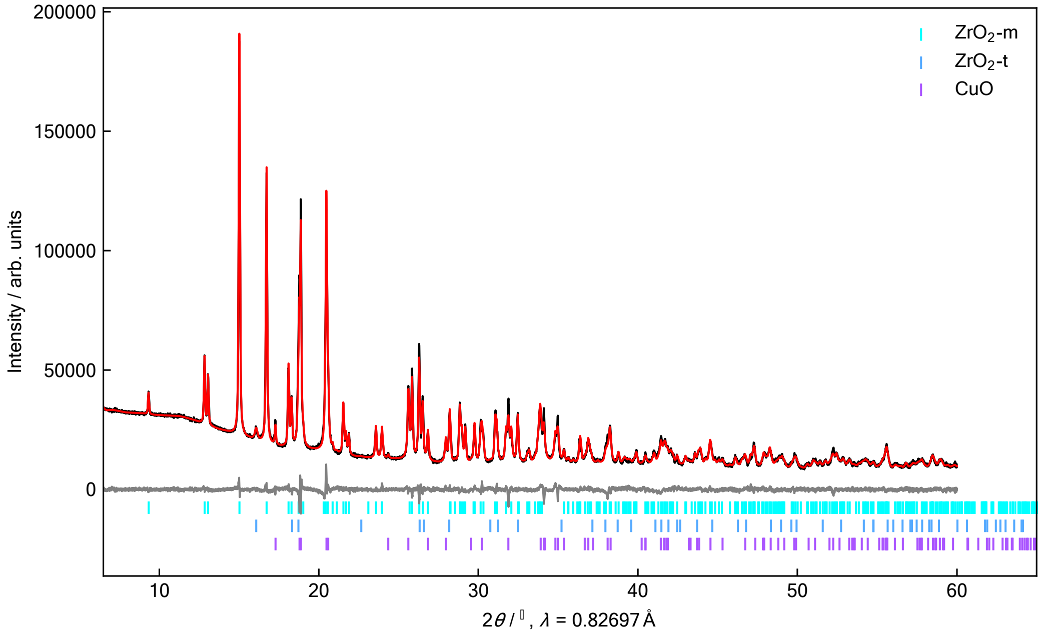

Figure S27: The PXRD and Rietveld refinement for the decomposition products from heating complex **6** at 800 °C for 4 hours in air.

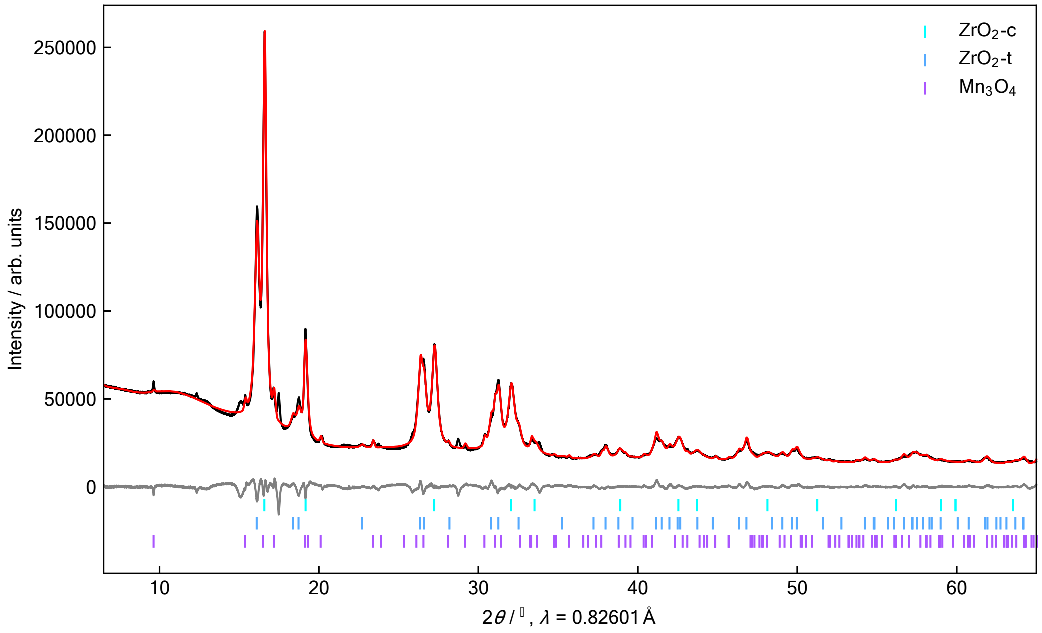

Figure S28: The PXRD and Rietveld refinement for the decomposition products from heating complex **7** at 800 °C for 4 hours in air.

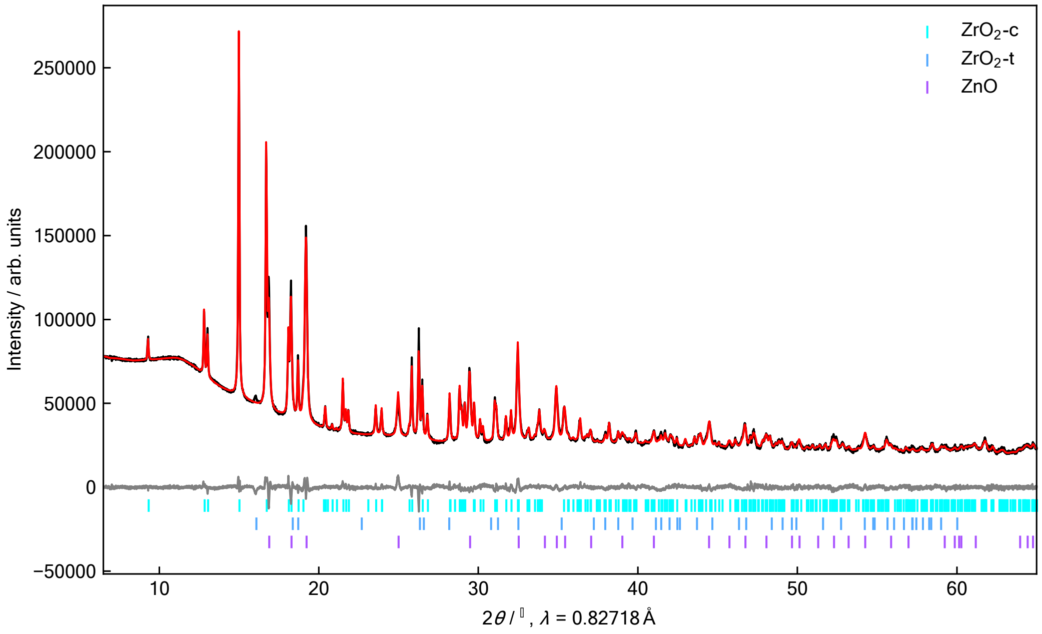

Figure S29: The PXRD and Rietveld refinement for the decomposition products from heating complex **8** at 800 °C for 4 hours in air.

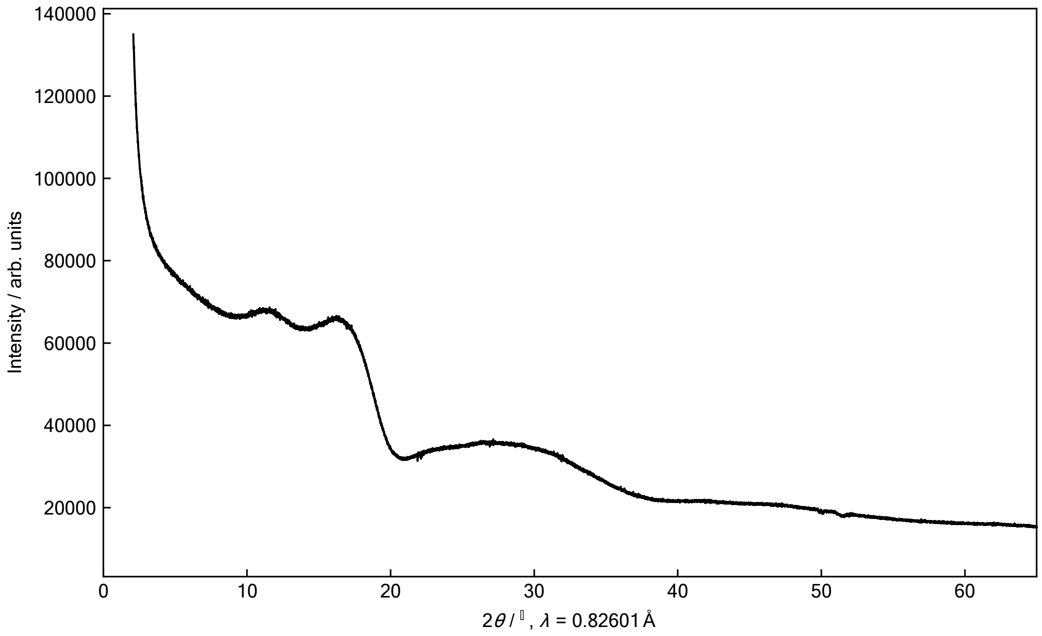

Figure S30: The PXRD for the decomposition products from heating complex **9** at 800 °C for 4 hours in air.

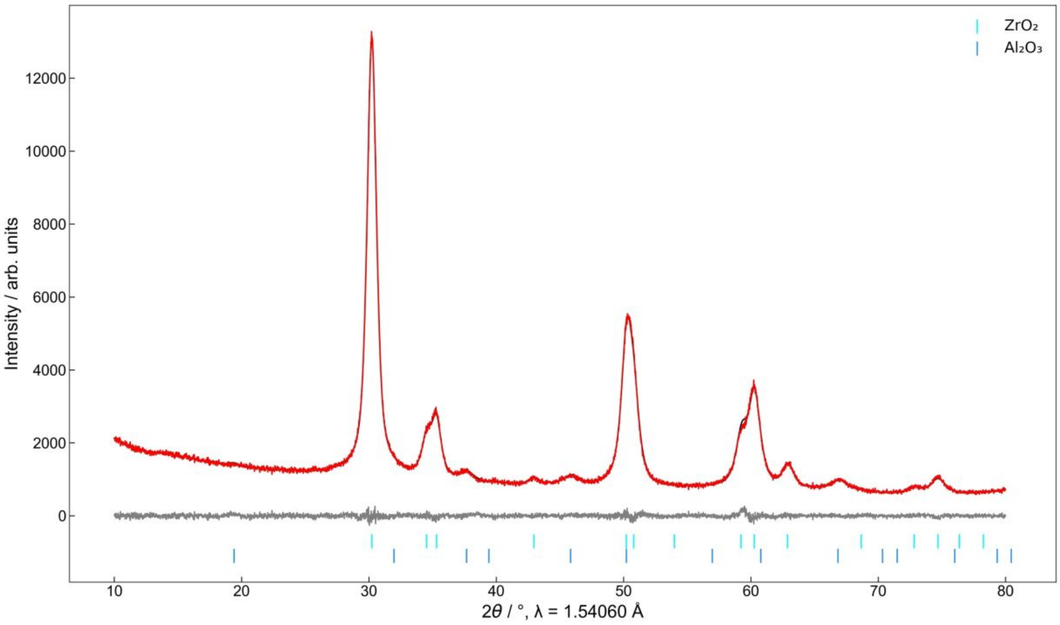

Figure S31: The PXRD and Rietveld refinement for the decomposition products from heating complex **9** at 1000 °C for 4 hours in air.

7. SEM

1

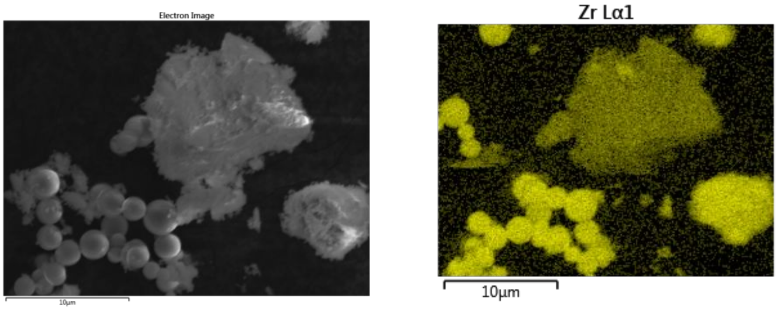
Electron Image
Zr Lα1
10μm

2

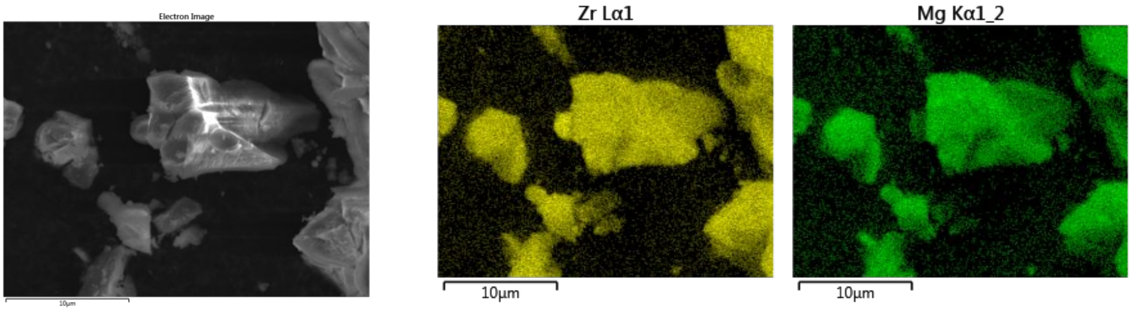
Electron Image
Zr Lα1
Mg Kα1_2
10μm
10μm

3

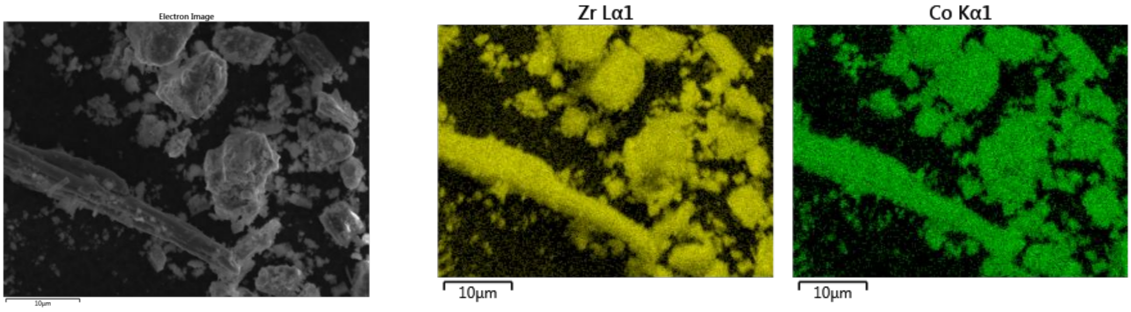
Electron Image
Zr Lα1
Co Kα1
10μm
10μm

4

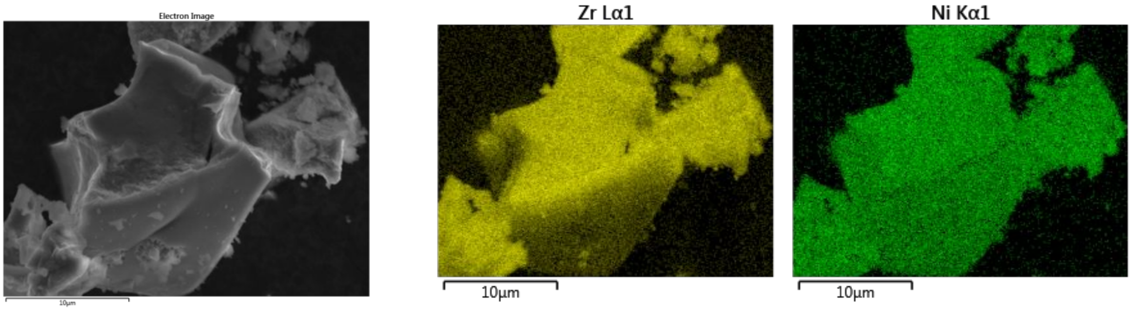
Electron Image
Zr Lα1
Ni Kα1
10μm
10μm

5

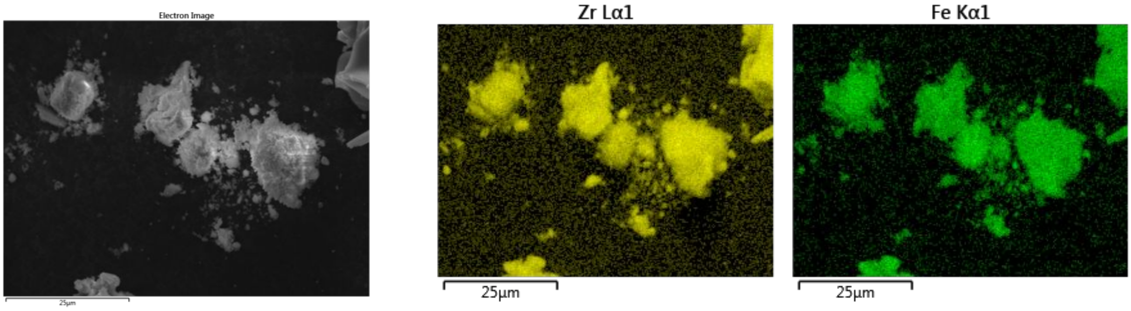
Electron Image
Zr Lα1
Fe Kα1
25µm
25µm

6

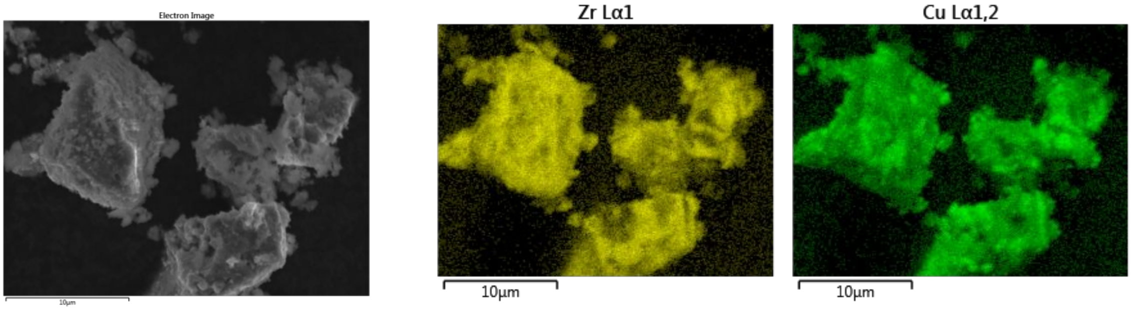
Electron Image
Zr Lα1
Cu Lα1,2
10µm
10µm

7

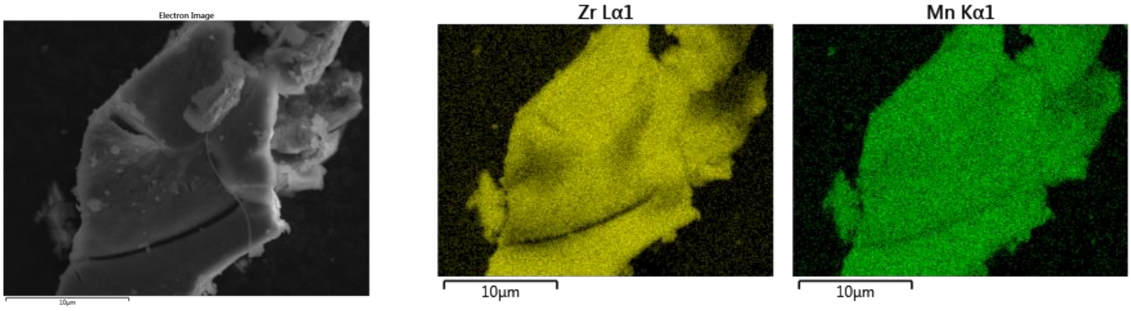
Electron Image
Zr Lα1
Mn Kα1
10µm
10µm

8

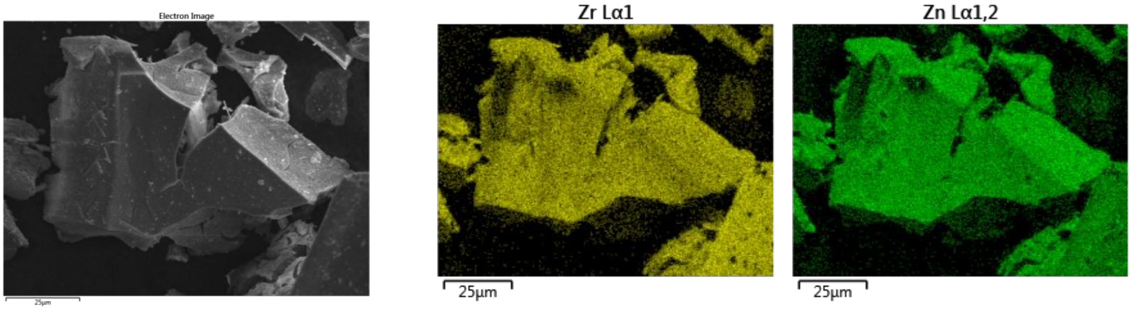
Electron Image
Zr Lα1
Zn Lα1,2
25µm
25µm

9

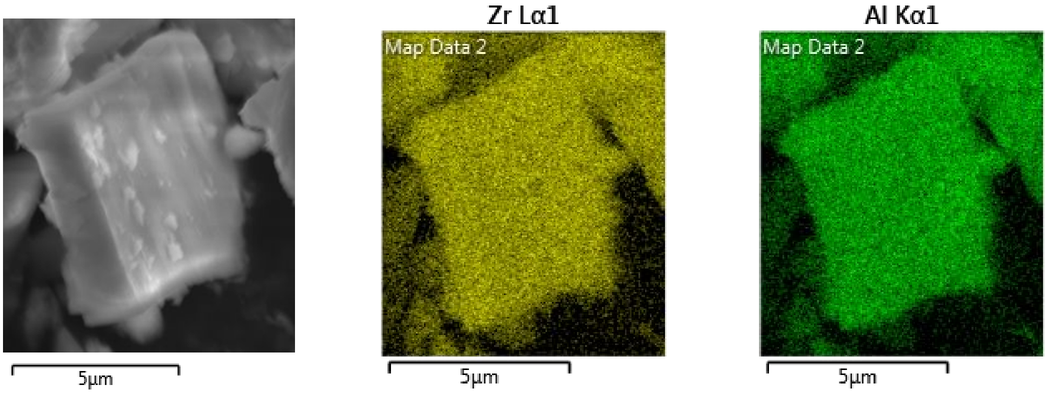

Figure S32: SEM/EDS images of the complexes **1**-**9** heated at 800 °C in air for 4 hours. The first column contains electron images of the samples taken with an electron energy of 20 keV. The second column shows EDS element maps of Zr and the third column shows maps of the other metal.
